# High-Resolution Digital Panorama of Multiple Structures in Whole Brain of Alzheimer's Disease Mice

**DOI:** 10.3389/fnins.2022.870520

**Published:** 2022-04-19

**Authors:** Xianzhen Yin, Xiaochuan Zhang, Jingjing Zhang, Weicheng Yang, Xian Sun, Haiyan Zhang, Zhaobing Gao, Hualiang Jiang

**Affiliations:** ^1^Center for MOST and Image Fusion Analysis, Shanghai Institute of Materia Medica, Chinese Academy of Sciences, Shanghai, China; ^2^CAS Key Laboratory of Receptor Research, Shanghai Institute of Materia Medica, Chinese Academy of Sciences, Shanghai, China; ^3^Lingang Laboratory, Shanghai, China; ^4^School of Chinese Materia Medica, Nanjing University of Chinese Medicine, Nanjing, China; ^5^Zhongshan Institute of Drug Discovery, Institution for Drug Discovery Innovation, Chinese Academy of Science, Zhongshan, China; ^6^School of Life Science and Technology, Shanghai Institute for Advanced Immunochemical Studies, ShanghaiTech University, Shanghai, China

**Keywords:** simultaneous visualization, multiple structures, Alzheimer's disease, micro-optical sectioning tomography, cross-scale

## Abstract

Simultaneously visualizing Amyloid-β (Aβ) plaque with its surrounding brain structures at the subcellular level in the intact brain is essential for understanding the complex pathology of Alzheimer's disease, but is still rarely achieved due to the technical limitations. Combining the micro-optical sectioning tomography (MOST) system, whole-brain Nissl staining, and customized image processing workflow, we generated a whole-brain panorama of Alzheimer's disease mice without specific labeling. The workflow employed the steps that include virtual channel splitting, feature enhancement, iso-surface rendering, direct volume rendering, and feature fusion to extract and reconstruct the different signals with distinct gray values and morphologies. Taking advantage of this workflow, we found that the denser-distribution areas of Aβ plaques appeared with relatively more somata and smaller vessels, but show a dissimilar distributing pattern with nerve tracts. In addition, the entorhinal cortex and adjacent subiculum regions present the highest density and biggest diameter of plaques. The neuronal processes in the vicinity of these Aβ plaques showed significant structural alternation such as bending or abrupt branch ending. The capillaries inside or adjacent to the plaques were observed with abundant distorted micro-vessels and abrupt ending. Depicting Aβ plaques, somata, nerve processes and tracts, and blood vessels simultaneously, this panorama enables us for the first time, to analyze how the Aβ plaques interact with capillaries, somata, and processes at a submicron resolution of 3D whole-brain scale, which reveals potential pathological effects of Aβ plaques from a new cross-scale view. Our approach opens a door to routine systematic studies of complex interactions among brain components in mouse models of Alzheimer's disease.

## Introduction

Amyloid-β (Aβ) plaque deposition is one major pathological hallmark of Alzheimer's disease (AD) (Murphy and LeVine, 2010). Aβ plaques are surrounded by multiple brain structures such as neurons, glia cells, nerve fibers, and blood vessels in the AD brain (Marin et al., 2016; Yuan et al., 2016; Miners et al., 2018; Greenberg et al., 2020). The structural abnormalities of these structures have been observed in the AD brains, which include cerebral amyloid angiopathy (Gurol et al., 2016), white matter (WM) lesion (Bozzali et al., 2002), ventricular enlargement (Apostolova et al., 2012), and brain atrophy (Pini et al., 2016). In addition, Aβ plaques-associated dystrophic axons (Adalbert et al., 2009), dendritic spine loss and synaptic alterations (Bittner et al., 2012), separation of astrocyte end-feet from the endothelial vessel wall (Kimbrough et al., 2015), recruitment and activation of glial cells (Fakhoury, 2018), and neuron death (Kadowaki et al., 2005) were also identified. These findings highlighted the essential contributions of in-depth analyzing the interactions of Aβ plaque with its surroundings to a thorough understanding of the pathological mechanism and progress of AD. However, the simultaneous visualization that shows how the plaques affect their surrounded neurons, nerve processes and tracts, and blood vessels in high resolution and whole brain is rarely achieved.

A number of two categories of methods have been developed and wildly used to image Aβ plaques. The labeling methods require specific reagents binding to aggregated Aβ, which include amyloid binding dyes thioflavin S or T (Bacskai et al., 2003), fluorochrome methoxy XO4 (Klunk et al., 2002), and Aβ-specific antibodies (Youmans et al., 2012), and *in vivo* near-infrared fluorescent probes such as DANIR 8c (Fu et al., 2016). In contrast, the label-free methods, such as stimulated Raman scattering microscopy (Ji et al., 2018), optical coherence microscopy (Bolmont et al., 2012), autofluorescence under liquid nitrogen freezing (Luo et al., 2017), multiphoton excitation fluorescence and second harmonic generation (Johansson and Koelsch, 2017), and MALDI mass spectrometry imaging (Enzlein et al., 2020), could avoid potential interference induced by exogenous molecules and provide more options for Aβ plaque imaging. Noticeably, the emerging of efficient brain-wide optical imaging techniques coupled with fast-developing amyloid labeling methods allows whole-brain imaging of Aβ plaques. Combining iDISCO, a novel clearing method integrating whole-mount antibody immunolabeling, the light-sheet microscopy permitted 3D detection of amyloid pattern in a full mouse brain hemisphere and archived human brain tissues (Liebmann et al., 2016). Fluorescence micro-optical sectioning tomography (fMOST) was successfully used to detect the brain-wide distribution of DANIR 8c labeled Aβ plaques in mice (Long et al., 2019). Similarly, serial two-photon tomography imaging of methoxy-X04 labeled plaques was applied to obtain the whole-brain imaging of amyloid pathology in AD mice (Whitesell et al., 2019). As to label-free brain-wide imaging of Aβ plaques, cryo-micro-optical sectioning tomography (cryo-MOST) was developed to acquire the whole-brain coronal distribution of Aβ plaques using intrinsic fluorescence emission under liquid nitrogen freezing (Luo et al., 2017).

To image brain vasculature, some new techniques drew more attention due to enhanced spatial resolution or extensive potential applications in comparison with the classic methods such as computed tomography angiography (Leiva-Salinas et al., 2018) and magnetic resonance angiography (Dorr et al., 2007). For example, the vascular corrosion cast coupled with stereomicroscopic scanning was utilized to visualize the 3D arrangement of the entire brain vasculature in APP23 mice (Meyer et al., 2008). Serial two-photon microscopy (Delafontaine-Martel et al., 2018) and light sheet microscopy (Di Giovanna et al., 2018) combined with intracardiac perfusion of FITC-gelatin perfusate achieved whole-brain vascular imaging at high resolution. Except the FITC-gelatin gel perfusion method, whole-mount immunolabeling (Kirst et al., 2020), fluorescent tagged transgenic mice (Jing et al., 2018), and conventional vascular-labeling dyes such as fluorescent lectin (Khouri et al., 2021) or lipophilic tracers (Konno et al., 2020) were also been adopted in light sheet microscopy technique for whole-brain vascular imaging. Interestingly, a novel method named “SeeNet” integrating multiple technologies (vascular casting with intravascular perfusion of fluorescent cross-linker, tissue clearing, and light sheet microscopy) achieved whole-brain visualization of cerebral vasculatures at the single-micro-vessel level (Miyawaki et al., 2020). Noticeably, the MOST/fMOST exhibited powerful capability to describe the entire vasculature of the mouse brain at high resolution with or without fluorescence labeling (Gang et al., 2017; Xiong et al., 2017). In one of our recent studies, using MOST and modified Nissl staining, we generated a cross-scale whole-brain 3D vascular atlases of AD mice, which covers the entire vascular system from large vessels down to the smallest capillaries at submicron resolution (Zhang et al., 2019).

Combined with the specific labeling, the whole-brain imaging techniques were also applicable to image and analyze specific-function neurons and neural circuits. *In vivo* fluorescence labeling such as intravascular injections of fluorescent dyes (Appaix et al., 2012), intracerebral injection of neurotropic viruses (Zingg et al., 2017), and varieties of fluorescent transgenic mice (Daigle et al., 2018) allows systematic imaging of molecularly labeled structures within the large intact brain. Serial two-photon tomography permitted collecting high-resolution datasets of fluorescent labeled Thy1-GFP mouse brains and also imaging adeno-associated virus (AAV)-injected brains expressing GFP for anterograde tracing (Ragan et al., 2012). Light sheet microscopy integrating tissue clearing and virus tracers or transgenic mice was widely used in mapping neural circuits (Jing et al., 2018; Mano et al., 2018). Lattice light-sheet microscopy coupled with expanded Thy1-GFP mouse brain realized nanoscale brain-wide optical imaging of neural circuits (Gao et al., 2019). Using the fMOST technique and multiple labeling, several brain research projects have sought to reconstruct high-resolution neuronal morphology of regions of interest and to provide the visualizations of neural pathways (Yuan et al., 2015).

These abovementioned advanced imaging methods permit high-resolution volume imaging of Aβ plaques, neurons, neurites, or blood vessels, individually. To study the complex influences of Aβ plaques in the AD brain, visualizing structures as many as possible in one same sample simultaneously will be greatly helpful. Due to the spectral overlap of the fluorophore and crossover of fluorescence emission, it is still of huge challenges to detect more than three fluorescent labels using fluorescence imaging techniques (McRae et al., 2019). Employing the reflected bright-field imaging technique of MOST with a whole-brain Nissl staining method, our previous study demonstrated the capability to distinguish different structures that include somata, blood vessels, and nerve processes based on the differences in gray values and morphologies (Zhang et al., 2019). In this study, taking advantage of the powerful capability of MOST in dissecting gray values and a newly developed customized image processing workflow for extraction and reconstruction of multiple structural signals, we successfully visualized the Aβ plaques, nerve tracts and nerve processes, somata, and blood vessels simultaneously in the whole brain of AD mice for the first time, without any specific labeling. This research provides a novel approach to clearly present various structural information in a whole mouse brain simultaneously, which will facilitate a better understanding of the cerebral anatomical features under the pathological state of AD.

## Results

### Acquisition of High-Content Characteristic Signals in Same Whole Mouse Brain

**Figure 1:**
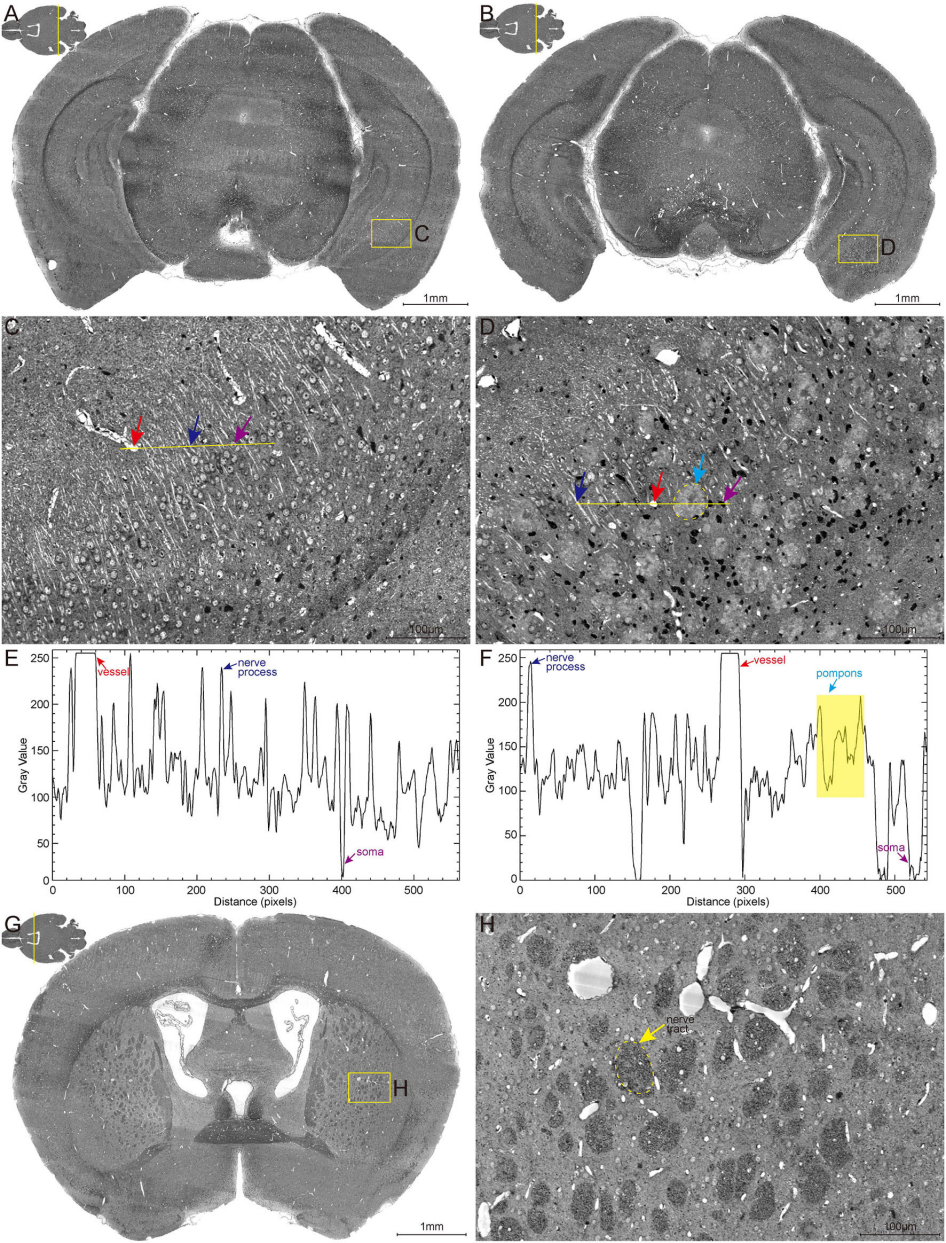
High-resolution brain atlas showing multiple signals of pompons, somata, nerve processes and tracts, and blood vessels. **(A,C,E)** WT group; **(B,D,F)** 5×FAD group. **(A,B)** Representative images of hippocampal coronal section in the 6-month-old WT and 5×FAD mice. **(C,D)** Enlarged views of the boxes in **(A,B)** show the multiple signals in Nissl staining data. Red, blue, purple, and cyan arrows denote vessels, nerve processes, somata, and pompons, respectively. **(E,F)** Two-dimensional graphs of the gray intensities of pixels along the drawn line in **(C,D)**. The x-axis represents distance along the line, and the y-axis is the gray intensity. The leftmost end of lines was corresponding to the leftmost end of the plot profile. Red, blue, purple, and cyan arrows denote the distinctive gray value of the representative vessels, nerve processes, somata, and pompons marked in **(C,D)**. **(G,H)** Representative image of coronal section across striatum in 5×FAD mouse shows the apparent signal of nerve tract. Enlarged views of the box in **(G)** are shown in **(H)**. The dotted box indicated by the yellow arrow denotes the nerve tract.

The high-content whole-brain datasets were obtained by combining the MOST system and whole-brain Nissl staining method. To acquire high contrast images, the bright-field MOST imaging was adjusted to a stable state with high image contrast during the time-consuming acquisition. Representative images of the coronal section of the whole-brain Nissl staining datasets in 6-month-old wild-type (WT) and 5×FAD mice (co-expressing five mutations of familial Alzheimer's disease) are shown in Figures 1A,B. The enlarged view in Figures 1C,D showed the different morphological features of multiple brain structures that include blood vessels (red arrow), nerve processes (blue arrow), and somata (purple arrow). The blood vessels were presented in a higher gray value comparing with surrounding somata and parenchyma, which makes them being easily visualized (Figures 1E,F). The signal of nerve processes was particularly evident in the strata radiatum of the hippocampus, with a gray value between that of vessels and somata (Figures 1C–F).

These abovementioned structures were observed with similar gray-level distribution in both WT and 5×FAD mice. Noticeably, a large amount of pompon-like structures, which obviously differed from other neuroanatomical brain structures, were observed in 5×FAD mice (cyan arrow in Figure 1D) rather than WT mice. Most of these pompon-like structures had a dark core surrounded by paler regions. Correspondingly, the gray-intensity plot of the pompons showed a specific “W” shape, owing to the relatively lower gray value in the center and the higher gray value in the rim (Figure 1F). In addition to these abovementioned signals, the massive dark circles in the striatum could also be identified according to their low gray level, large diameter, and distinctive morphology. As shown in Figures 1G,H, the massive dark circles in the striatum were supposed to be nerve tract. In conclusion, multiple discernible signals of different structures were simultaneously acquired using the whole-brain Nissl staining and MOST system.

### Customized Image Processing Workflow for Identification and Extraction of Multiple Structures

Although the characteristic signals of somata, blood vessels, and nerve processes and tracts had been acquired, the visual rendering of these multiple structures encountered considerable obstacles due to the heterogeneous gray levels and low contrast of the bright field images. To identify and validate exactly what the abovementioned characteristic signals are, a customized image processing workflow for extraction and reconstruction of these multiple structures was developed (Supplementary Figure 1). Initially, the coronal images were optimized to enhance image quality through the steps that include background correction, noise reduction, and contrast enhancement, which had been described in our previous study (Zhang et al., 2019). The enhanced contrast of the optimized high-quality coronal images made it more feasible to identify the ultrastructure of these structures based on the grayscale and morphological differences. The vessels in the highest gray ranges and the somata in the lowest gray ranges could be easily visualized. In addition, the information of processes, tracts, and plaques was difficult to be extracted due to their adjacent gray-value distribution. Therefore, the virtual channel splitting method was performed to individually extract the characteristic structure information of blood vessels, pompons, and neural structures. The vascular information was filtered out by region growth algorithm and then was reconstructed using iso-surface rendering; the pompon information was visualized by high-precision real-time rendering after feature enhancement; the neural information that includes soma, nerve process, and tract was extracted and then performed direct volume rendering. At last, all the separated virtual channels were integrated through feature fusion to complete synchronous visualization of multiple structures.

**Figure 2:**
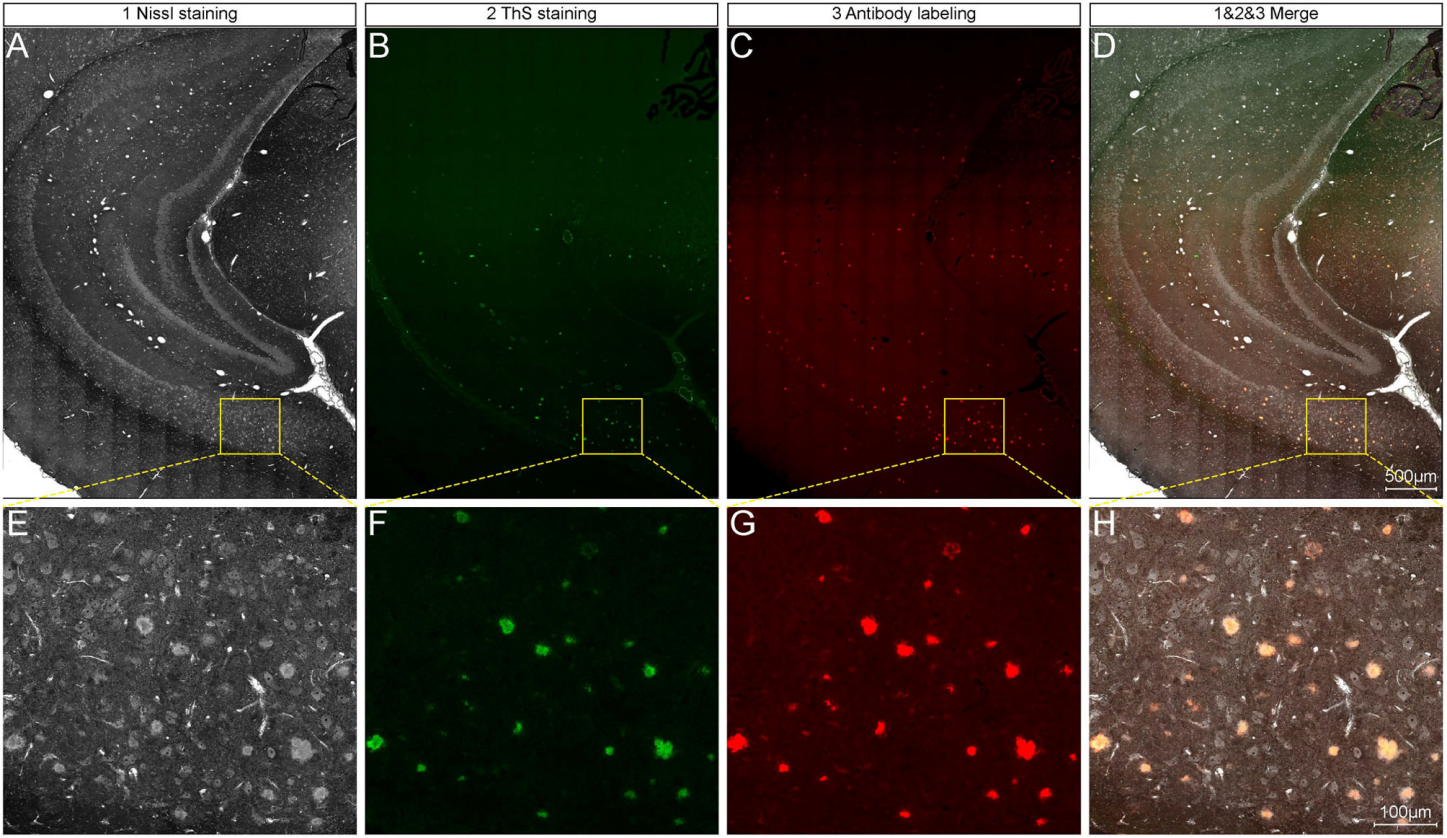
Comparison between whole-brain Nissl staining and Aβ-specific staining results on the same tissue section. **(A–C)** Representative images showing plaque signals identified by whole-brain Nissl staining **(A)**, ThS staining **(B)**, and antibody labeling **(C)** on the same tissue section of 6-month-old 5×FAD mice. **(D)** Overlapping of images in **(A–C)**. Enlarged views of the boxes in **(A–D)** are corresponding shown in **(E–H)**.

After the extraction and reconstruction of 3D architectures, the characteristic signals of neural structures and blood vessels were identified (Supplementary Figure 1) and were also confirmed by referring to the observed structures in the other studies (Wu et al., 2014; Xiong et al., 2017). The pompons observed in this study could not be classified as any reported neural structures, but resembled the morphology of Aβ plaques observed in other imaging studies using Thioflavin S staining (Dickson and Vickers, 2001). To examine whether the pompon-like structures were Aβ plaques, we performed whole-brain Nissl staining, thioflavin S (ThS) staining, and anti-Aβ antibody labeling on the same paraffin tissue sections for comparison. The large-size images of the coronal brain section from a 6-month-old 5×FAD mouse are shown in Figures 2A–D. Typical brain architectures were easily recognized in all images, which include the cortex, hippocampal cornu ammonis, and dentate gyrus. The enlarged views of yellow boxes in Figures 2A–D were displayed in Figures 2E,H, respectively. From the representative images of the Nissl staining coronal section in Figures 2A,E, the pompon-like structures can be clearly distinguished. Both the ThS fluorescence (Figures 2B,F) and antibody immunofluorescence (Figures 2C,G) were observed on the same coronal section as shown in Figures 2A,E. In regions where Aβ plaques were detected by ThS and anti-Aβ antibody, whole-brain Nissl staining could also successfully label them with one-to-one correlation. The merged images in Figures 2D,H illustrated the co-localization of Nissl, ThS, and anti-Aβ antibody labeled Aβ plaques. These results demonstrated that the pompon-like structures obtained by the whole-brain Nissl staining method were indeed Aβ plaques.

### Generation of All-in-One Panorama for Aβ Plaques, Somata, Nerve Processes and Tracts, and Blood Vessels in Whole Mouse Brain

#### Brain-Wide Visualization of Aβ Plaque Distributions

**Figure 3:**
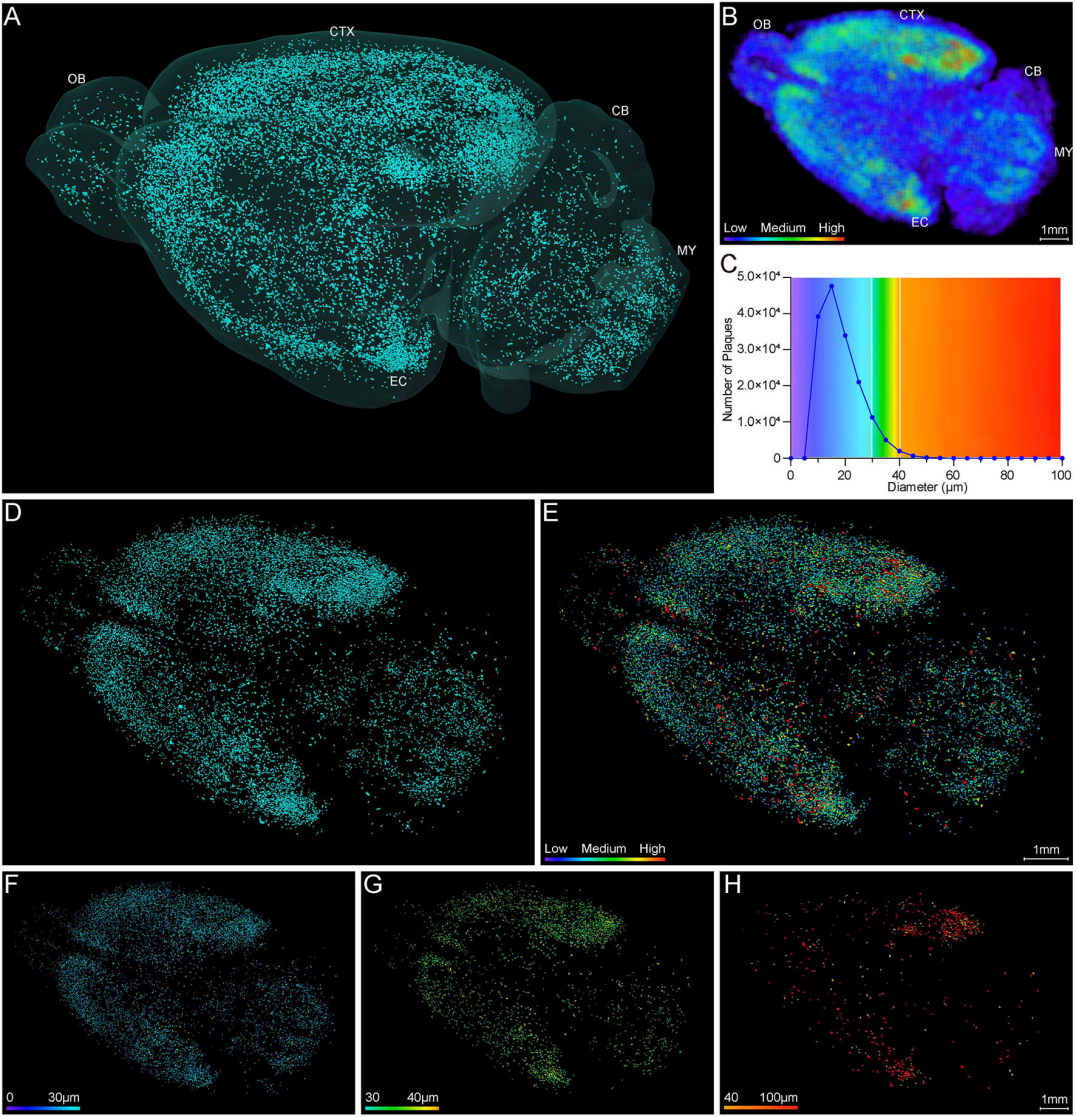
Whole-brain 3D visualization of anatomical distribution of Aβ plaques. **(A)** 3D lateral view of the whole brain amyloid plaque distribution in the 5×FAD mice. **(B)** A 3D heatmap of plaque density distribution from apical view. The density of plaques was coded by a color scale and deep blue represents the lowest density. **(C)** Statistical analysis of the number of plaques in different diameters. The colors above x-axis were coded by the diameter of plaques, which were displayed according to different diameter ranges. **(D,E)** Top view of the amyloid plaque distribution throughout the whole brain and corresponding diameter distribution coded by a color scale and deep blue represents the lowest diameter. **(F–H)** The whole-brain plaques in different diameter ranges are separately shown: <30 μm **(F)**, 30–40 μm **(G)**, > 40 μm **(H)**. OB, olfactory bulb; CTX, cerebral cortex; CB, cerebellum; MY, medulla; EC, entorhinal cortex.

Systematic whole-brain visualization of Aβ plaque distribution is critical to characterize the key pathological features for understanding the pathology of AD. Figure 3A and Supplementary Movie 1 showed the distribution of Aβ plaques across an entire brain of a 5×FAD mouse. The brain-wide plaque distribution from the olfactory bulb to cerebellum was clearly visualized, and the plaques were observed throughout the whole brain. A 3D heatmap of whole-brain plaque distribution density was shown in Figure 3B and Supplementary Movie 2. The deeper layer of the cortex exhibited a denser plaque distribution, and the entorhinal cortex along with its adjacent subiculum regions presented the highest density of plaques. To confirm the plaque distribution, we compared the plaque numbers in the entorhinal cortex and subiculum regions with those in their neighboring regions. Table 1 showed that the plaque numbers in the entorhinal cortex and subiculum regions were much larger than those in the adjacent cortex and hippocampal CA3 regions, which was consistent with the results from the 3D heatmap. Quantification analysis of the whole brain plaque number in different diameters revealed that the Aβ plaques smaller than 40 μm accounted for the majority of total plaques (Figure 3C). In addition, a top view of the brain-wide plaque distribution and its corresponding diameter-coded colormap were displayed in Figures 3D,E, respectively. The plaque distribution pattern was analyzed on three diameter ranges: diameter < 30 μm, 30 μm < diameter <40 μm, diameter > 40 μm (Figures 3F–H). In accordance with the quantification analysis in Figure 3C, the plaque number in these three ranges of diameter exhibited huge differences. The Aβ plaques smaller than 30 μm in diameter were the most plentiful, and there was no apparent difference in plaque density across the whole brain (Figure 3F). The Aβ plaques between 30 and 40 μm in diameter were relatively smaller in amount, and the plaque density varied obviously among different brain regions (Figure 3G). The cortical regions exhibited a denser plaque distribution, but the olfactory bulb, cerebellum, and deeper brain regions of the cerebrum had less plaques than the cortical regions (Figure 3G). In contrast to that most plaques were less than 40 μm in diameter, only few Aβ plaques exceeded 40 μm in diameter and were mainly distributed in the regions of the entorhinal cortex and adjacent subiculum (Figure 3H). The total and average plaque volumes in the regions of entorhinal cortex and adjacent subiculum were also much larger than those in the adjacent cortex and hippocampal CA3 regions (Tables 2, 3). The highest density and largest size of plaques in the entorhinal cortex and adjacent subiculum suggested that Aβ plaques might first appear in these related regions and gradually increase with age.

**Table 1:** Comparison of plaque numbers between the Entorhinal cortex and adjacent cortex regions, as well as between the hippocampal subiculum and CA3 regions.

**Brain regions**		**Plaque number**		
		**Sample 1**	**Sample 2**	**Sample 3**
Cortex	Entorhinal cortex	655	484	331
	Adjacent cortex	218	130	112
Hippocampus	Subiculum	826	634	573
	CA3	130	101	111

*The plaque numbers of these brain regions were calculated for three voxel blocks (0.5 × 0.5 × 0.5 mm^3^, one block per mouse) from three mice. The voxel blocks were selected and cropped from the similar anatomical position of the brain of these three mice. These three voxel blocks were also employed for calculating total and average plaque volumes (Tables 2, 3)*.

**Table 2:** Comparison of total plaque volumes between the Entorhinal cortex and adjacent cortex regions, as well as between the hippocampal subiculum and CA3 regions.

**Brain regions**		**Total plaque volume (μm^3^)**		
		**Sample 1**	**Sample 2**	**Sample 3**
Cortex	Entorhinal cortex	6.76E+06	5.41E+06	1.92E+06
	Adjacent cortex	1.25E+06	4.50E+05	2.42E+05
Hippocampus	Subiculum	7.30E+06	4.71E+06	6.75E+06
	CA3	2.43E+06	1.97E+05	2.60E+05

**Table 3:** Comparison of average plaque volumes between the Entorhinal cortex and adjacent cortex regions, as well as between the hippocampal subiculum and CA3 regions.

**Brain regions**		**Average plaque volume (μm^3^)**		
		**Sample 1**	**Sample 2**	**Sample 3**
Cortex	Entorhinal cortex	1.03E+04	1.12E+04	5.80E+03
	Adjacent Cortex	5.73E+03	3.46E+03	2.16E+03
Hippocampus	Subiculum	8.84E+03	7.43E+03	1.18E+04
	CA3	1.87E+04	1.95E+03	2.34E+03

#### Synchronous Visualization of Aβ Plaques, Somata, Nerve Tracts, and Blood Vessels in Whole Mouse Brain

**Figure 4:**
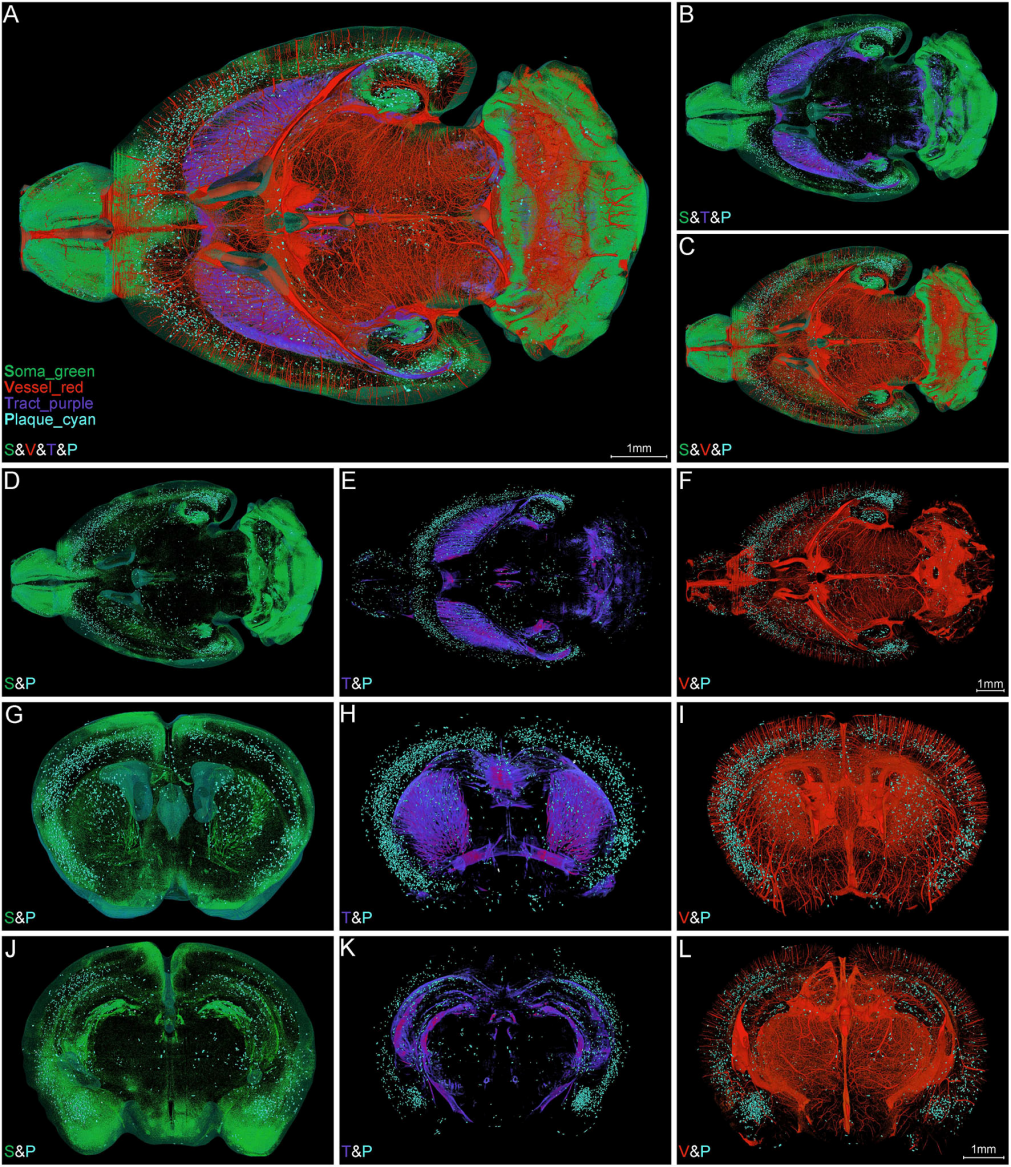
Simultaneous whole-brain visualization of Aβ plaques, somata, nerve tracts, and blood vessels. **(A–F)** Simultaneous whole-brain visualization of multiple signals in the 5×FAD mice with different structures coded by different pseudo-colors. The “S,” “V,” “T,” or “P” in the left bottom of each image refer to soma, vessel, tract and plaque, respectively (the same below). **(A)** Soma, vessel, tract, and plaque. **(B)** Soma, tract, and plaque. **(C)** Soma, vessel, and plaque. **(D)** Soma and plaque. **(E)** Tract and plaque. **(F)** Vessel and plaque. **(G–L)** Simultaneous visualization of multiple signals in the representative coronal slices across striatum **(G–I)** and hippocampus **(J–L)** in the same mouse as shown in **(A)**. **(G,J)** Soma and plaque. **(H,K)** Tract and plaque. **(I,L)** Vessel and plaque. Soma, vessel, tract, and plaque are shown in green, red, purple, and cyan, respectively (the same below).

To ascertain the spatial correlation between Aβ plaques and their surrounding brain structures, we visualized the Aβ plaques, somata, nerve tracts, and blood vessels simultaneously in the intact brain of 5×FAD mice. Figure 4A shows the colocalization of somata (green), blood vessels (red), nerve tracts (purple), and Aβ plaques (cyan) across the entire brain. The high-precision rendering of whole brain revealed the 3D images of multiple brain components with structure-specific pseudo-colors. Owing to the presence of massive dense granule cells, the visualization of somata (green) showed distinctive cytoarchitectonic features in the olfactory bulb and cerebellum (Figures 4A–C). Figure 4B shows the spatial distribution difference among Aβ plaques, somata, and nerve tracts. Apparently, the nerve tracts principally spread over the WM areas, whereas the somata were mainly distributed in the gray matter (GM) areas. In the cerebrum, the Aβ plaques were densely distributed in the deep layer other than the superficial layer of the cortex, and the distribution of Aβ plaques was correlated with that of somata and nerve tracts (Figures 4B–E). The Aβ depositions were obviously much denser in the GM areas where were full of somata, as compared to those in the WM areas where full of tracts (Figures 4D,E). In addition, the spatial distribution of Aβ plaque also showed a correlation with the vascular distribution. Plaque-dense areas were generally localized in the distal end rather than root of vessels (Figures 4C,F).

The representative images of coronal slices across the striatum (Figures 4G–I) and hippocampus (Figures 4J–L) also exhibited the similar Aβ plaque distribution pattern. From these local high-resolution perspectives, the plaques were relatively enriched in soma-dense areas (Figures 4G,J) and were sparse in tract-dense areas (Figures 4H,K). The blood vessels were largely arranged in parallel and penetrated the deep cortical layer. The plaques were mainly aggregated in the distal end of the blood vessels (Figures 4I,L). In conclusion, the plaque-dense areas exhibited more somata but less nerve tracts and localized in the distal end of vessels.

### Spatial Distribution Correlation of Aβ Plaques, Somata, and Blood Vessels in Cortex and Hippocampus

#### Aβ Plaque, Somata, and Blood Vessels in Cortex

**Figure 5:**
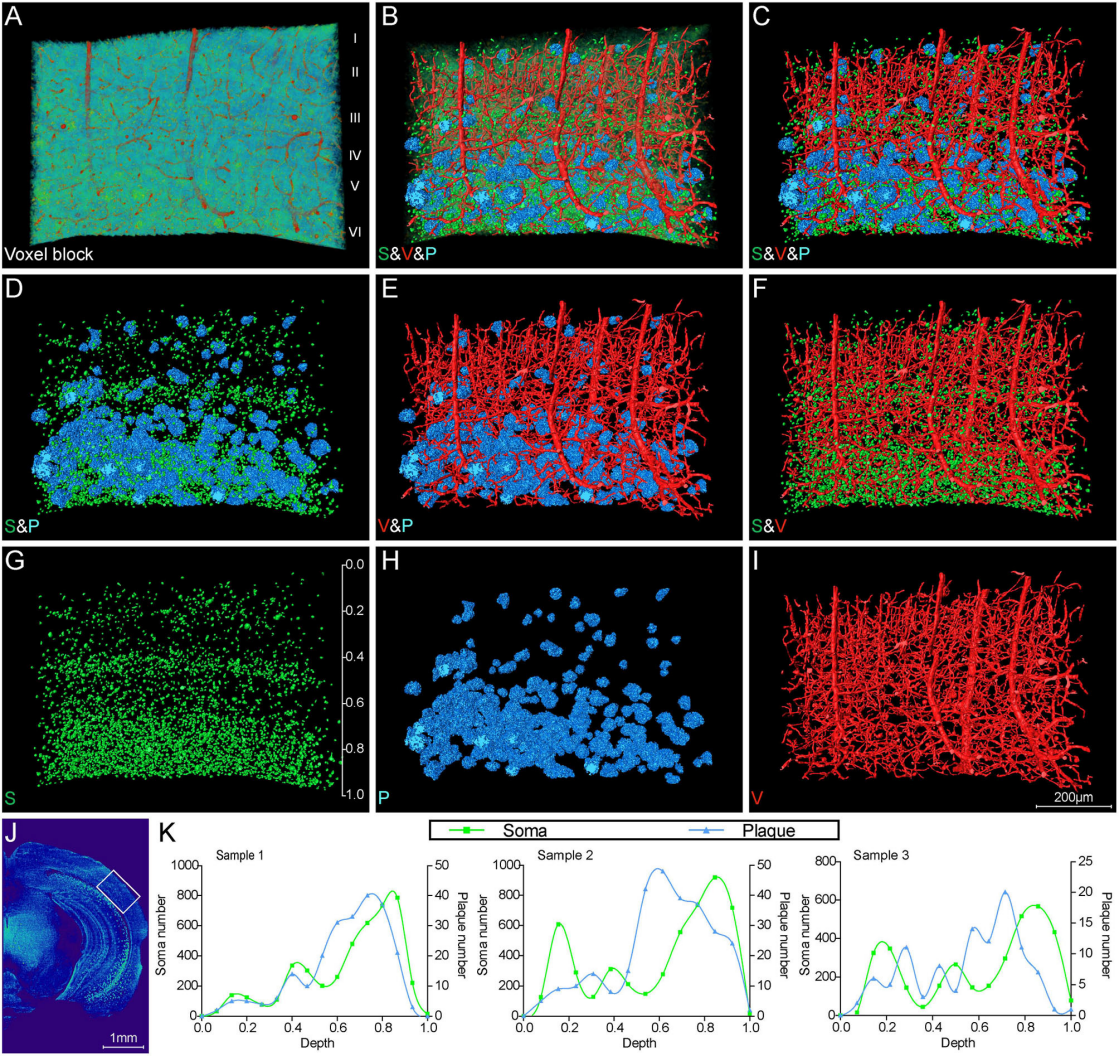
Visual reconstruction of Aβ plaques and adjacent somata and vessels in cortical area. **(A)** Representative voxel block from the cortical region with default colormaps in the 5×FAD mice. **(B–I)** The same voxel blocks as shown in A were displayed with plaques, somata, and vessels to illustrate the spatial relationship among these signals. **(J)** The boxed area indicated the position of the voxel block as shown in **(A–I)**. **(K)** Quantitative analysis of the number of somata and plaques with cortical depth changing based on images of 200-μm-thick coronal slices similar to that shown in **(J)**. The left y-axis denoted the number of somata and the right y-axis denoted the number of plaques. The x-axis represented cortical depth which was normalized by the whole cortical size of each sample.

To further investigate the correlation of Aβ plaques, somata, and blood vessels in local regions, we performed a 3D volume rendering of the cortical voxel blocks. The voxel block cropped from the cortical area as shown in Figure 5A exhibited the representative cerebral architecture in the 5×FAD mice. Figures 5B–I depict the images of the same section as shown in Figure 5A over a wider range of gray levels (color-coded) that revealed Aβ plaques, somata, and blood vessels. Figures 5B,C revealed 3D images of Aβ plaques, somata, and blood vessels with structure-specific pseudo-colors. By viewing somata and plaques (Figure 5D), we confirmed that the Aβ plaques were prone to accumulate in the deeper layer of the cortex, which could also be observed from Figures 3B, 4B,D,G,J. The deeper layer of the cortex had more abundant somata and distal vessels (Figures 5D–I). The visualization of somata and vasculatures in WT mice also depicted the similar distribution pattern (Supplementary Figures 2A–E). The normal cortex showed an apparently layered distribution of somata, and the soma number varied in different layers, reaching the highest in the deeper layer (Supplementary Figures 2C,E). In addition, we performed a quantitative analysis on the number of somata and plaques with cortical depth changing based on the images of 200-μm-thick coronal sections similar to that shown in Figure 5J. A total of three 5×FAD mice samples were employed to calculate the spatial relationship between somata and plaques, and the x-coordinate of cortical depth was normalized by the whole cortical size (Figure 5K). The quantitative results revealed that the depth with the largest number of plaques was in the vicinity of that with the largest number of somata, which demonstrated that the plaques were prone to distribute near the location that more somata gathered. These results demonstrated that there was a strong spatial distribution correlation between Aβ plaques and somata.

#### Aβ Plaque, Somata, and Blood Vessels in Hippocampus

**Figure 6:**
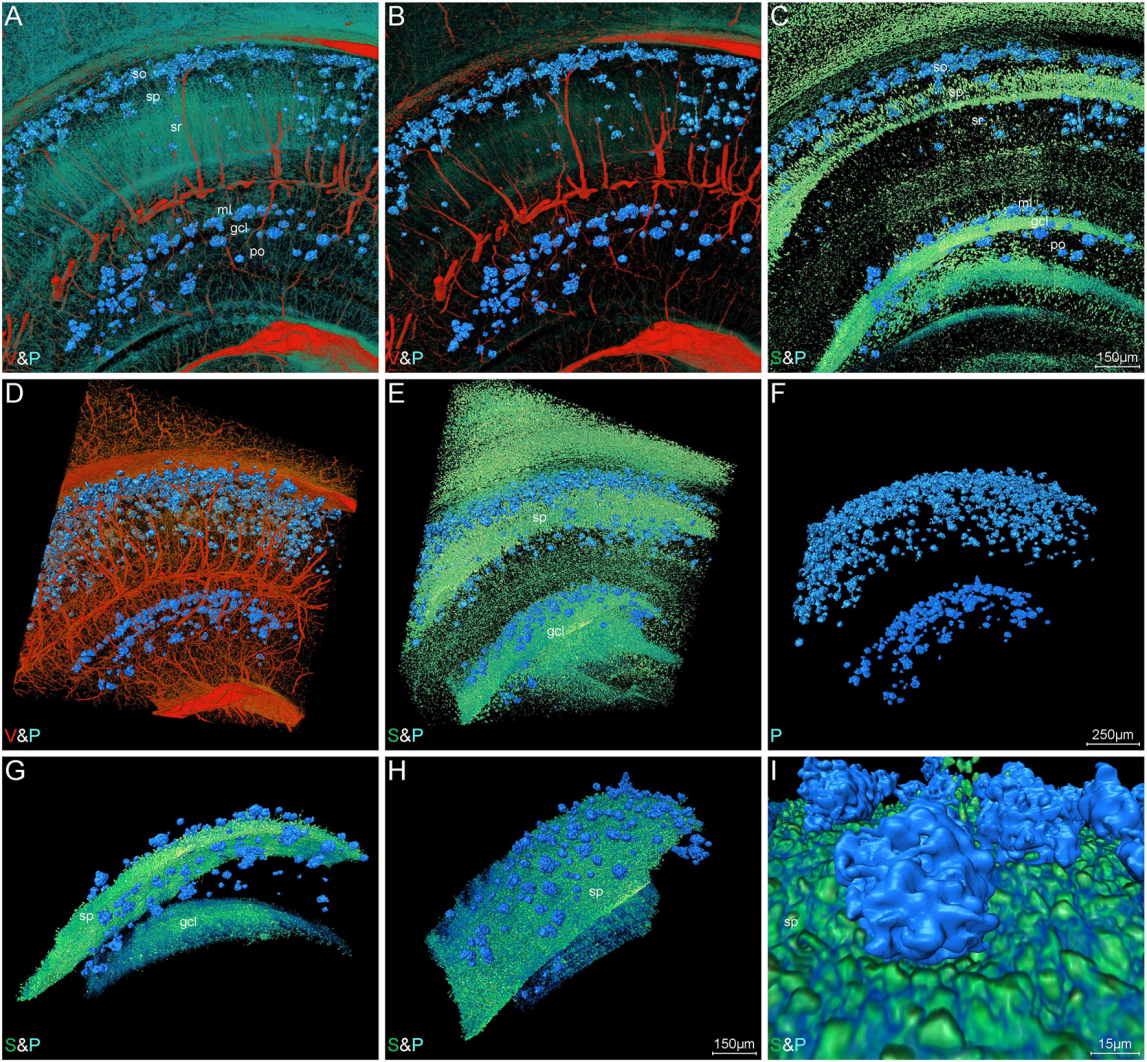
Visual reconstruction of Aβ plaques and adjacent somata and vessels in hippocampal area. **(A–C)** Representative coronal view of 200-μm-thick slices showing Aβ plaques and other structures in the hippocampus of 5×FAD mice. **(D–I)** 3D volume rendering of 400-μm-thick sections across the same coronal slice as shown in A describing the specific plaque distribution. **(D)** The Aβ plaques were dispersed abundantly in the areas of the distal end of blood vessels. **(E,F)** The Aβ plaques were prominently distributed into a layer and the majority were close to the cell layer, namely stratum pyramidale and granule cell layer. **(G,H)** Lateral view of the extracted cell layer, with stratiform Aβ plaques visible adjacently to the cell layer. **(I)** The magnified view showing the Aβ plaques embedded in the cell layer. In the Cornu Ammonis, so, stratum oriens; sp, stratum pyramidale; sr, stratum radiatum. In the dentate gyrus, ml, molecular layer; po, polymorph layer; gcl, granule cell layer.

The representative images of hippocampal regions also depicted a characteristic Aβ plaque distribution. Figures 6A–I show the Aβ plaques, somata, and blood vessels in 200-μm-thick coronal sections across the hippocampus. The plaques were predominantly distributed in the stratum oriens of cornu ammonis and the molecular layer of dentate gyrus (Figures 6A–C). It should be noted that the plaques in the stratum oriens and molecular layer were in close proximity to the stratum pyramidale and granule cell layer, as well as located in the distal end of blood vessels. These results indicated the distribution correlation of the plaques, somata, and blood vessels in the hippocampus, which was also in accordance with the visualization results in the whole brain (Figures 4B–D,F). There were few plaques appearing in the stratum radiatum, which was full of neuronal processes (Figure 6A). High-precision volume rendering of 400-μm-thick slices similar to the same coronal slice as shown in Figure 6A also described the characteristic plaque distribution (Figures 6D–F). By visualizing the plaques and vessels, we found that more abundant plaques were distributed in the distal end rather than in the root of vessels (Figure 6D). The normal distribution relationship of hippocampal structures is shown in Supplementary Figures 2F–J, which demonstrated the specific distribution of somata, vessels, and neuronal processes in different hippocampal subregions. To further define the spatial relationship between plaques and somata, we extracted the plaques and their neighboring cell layers including both pyramidal cell and granule cell layers (Figures 6E–I). The volume rendering of hippocampal voxel block in Figures 6E,F showed the stratiform plaques in the vicinity of cell layers. From the extracted images, we found some plaques embedded in the cell layer, which leads to a notch in the intact cell layer (Figures 6G–I). In conclusion, these results revealed a characteristic layered distribution pattern of Aβ plaques in both cortical and hippocampal regions.

### Structural Alternations of Nerve Processes and Capillaries Surrounding Aβ Plaques at Subcellular Resolution

#### Abnormalities of Neuronal Processes Surrounding Aβ Plaques

**Figure 7:**
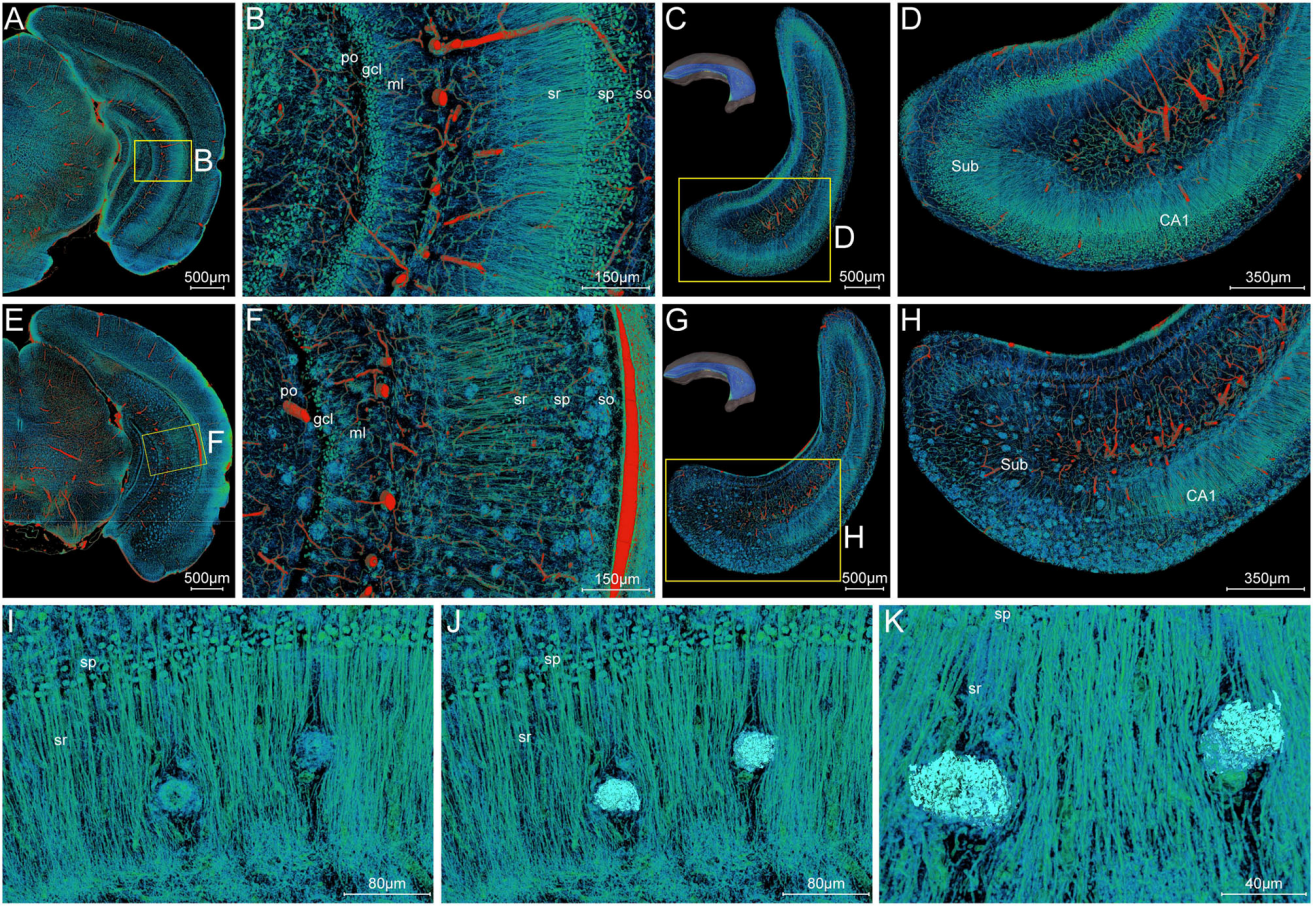
Visualization of neuronal processes surrounding Aβ plaques. **(A–D)** Simultaneous visualization of neuronal processes and somata in the WT mouse. **(E–H)** Simultaneous visualization of Aβ plaques, neuronal processes, and somata in the 5×FAD mouse. **(A,B,E,F)** Representative image of the typical coronal slice across the hippocampus and the magnification of the boxed area. **(C,D,G,H)** Representative image of the typical slice parallel to the longitudinal hippocampal axis across the hippocampus and the magnification of the boxed area. Sub, subiculum. **(C,G)** The unilateral hippocampus in the upper left corner indicated the location of the reconstructed slices parallel to the longitudinal hippocampal axis. **(I–K)** Representative high-resolution images of the strata radiatum in the hippocampus showing the typical Aβ plaques and their surrounding neuronal processes. The same view showed the Aβ plaques displayed by volume rendering **(I)** and surface reconstruction **(J)** in the compact network of neuronal processes. The more clear view in **(K)** showed the neuronal processes with abrupt branch ending or bending. In the Cornu Ammonis, so, stratum oriens; sp, stratum pyramidale; sr, stratum radiatum. In the dentate gyrus, ml, molecular layer; po, polymorph layer; gcl, granule cell layer.

To draw a precise map of the plaque deposition and adjacent nerve processes, we made simultaneous visualization of Aβ plaques and nerve processes at subcellular resolution in the local hippocampal regions. Figures 7A–D show the representative images of coronal and longitudinal sections of nerve processes in the right hippocampus of the WT mice. The processes in the stratum radiatum appeared compact spoke-like extensions deriving from the somata in the stratum pyramidale (Figures 7B,D). Compared with those processes in the WT mice, the processes showed apparent abnormalities in the 5×FAD mice (Figures 7E–H). The plaques were dispersed over the stratum oriens, stratum radiatum, and subiculum. It is noted that the processes were nearly absent in areas where plaques aggregated heavily. The processes were remarkably reduced in the subiculum region (Figures 7G,H), which was also consistent with the highest plaque density in the subiculum region as shown in Figure 3B. To further focus on typical individual Aβ plaque and surrounding nerve processes, we performed a visual reconstruction of typical region in the stratum radiatum of cornu ammonis at subcellular resolution (Figures 7I–K). The processes passing through or near Aβ plaques exhibited various abnormalities including abrupt branch ending or bending. In addition, we found that a cluster of smaller somata, generally identified as microglia cell or astrocytes in the previous imaging studies (Liu et al., 2010; Serrano-Pozo et al., 2011), was dispersed in or around the Aβ plaques (Supplementary Figures 3A–F, S4A–I). These plaque-associated somata were smaller in size and appeared darker in grayscale Nissl staining images (Supplementary Figures 3B–F).

#### Deformation of Capillaries Associated With Aβ Plaques

**Figure 8:**
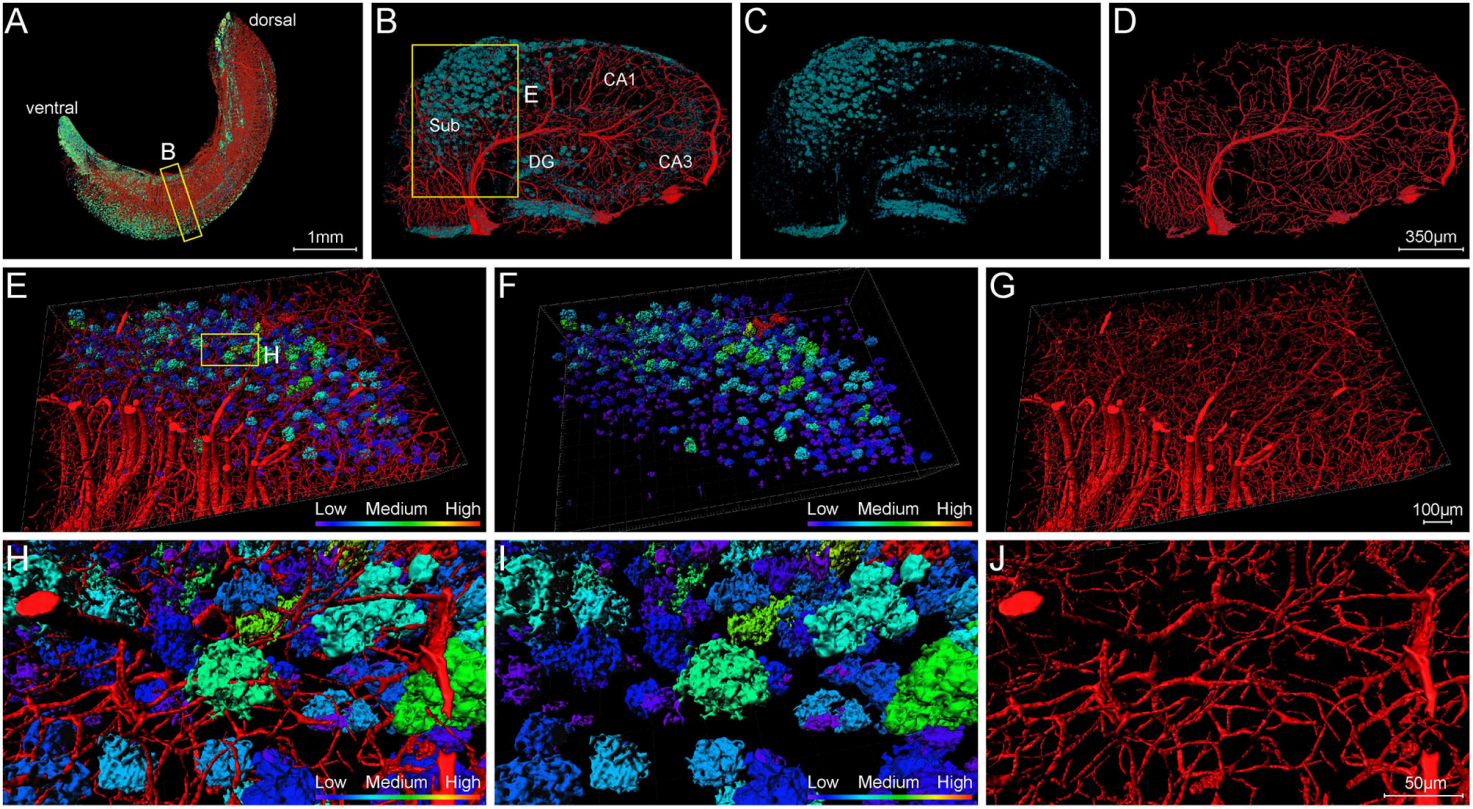
Visualization of blood vessels surrounding Aβ plaques. **(A)** Front view of Aβ plaques and blood vessels in the right hippocampus showing the abundant plaques in the ventral part of the hippocampus of 5×FAD mice, with vessels eliminating. The sectional view of the box area in **(A)** is enlarged and shown in **(B)**. **(B–D)** Representative sectional views of hippocampal vasculature and plaque burden perpendicular to the longitudinal hippocampal axis. **(B)** Vessel and plaque. **(C)** Plaque. **(D)** Vessel. **(E–G)** The magnifications of the box in B showing the plaques in the compact vascular network. **(E)** Vessel and plaque. **(F)** Plaque. **(G)** Vessel. **(H–J)** The magnifications of the box in E depicting the interaction of plaques and vessels. **(H)** Vessel and plaque. **(I)** Plaque. **(J)** Vessel.

To clearly analyze the interaction between Aβ plaques and vasculatures in local regions, we visualized the plaques and vasculatures simultaneously in the hippocampus. The distribution of plaques and vasculatures across the entire unilateral hippocampus is shown in Figure 8A. The plaques were found throughout the hippocampus. Besides, the plaque-denser areas in the ventral part exhibited a reduction of blood vessels, and the plaque-sparser areas in the dorsal part showed relatively richer blood vessels (Figure 8A). These results demonstrated the substantial disruption of the hippocampal angioarchitecture. Actually, with respect to the cortical architecture, the plaque led to the holes in the compact network of nerve cells (Supplementary Figures 4A–C), and the capillaries were impaired heavily with severe fragmentation and truncation (Supplementary Figures 4D–F). To further present the detailed fine-scale description of the vasculature, a representative view of cross-sections perpendicular to the longitudinal hippocampal axis is displayed (Figures 8B–D). The Aβ plaques aggregated heavily in the region of the subiculum (Figures 8B,C), in which the vasculature was accordingly damaged severely (Figures 8B,D). The cross-view of normal vasculature in WT mice is shown in Supplementary Figure 2I, with no plaques in the vascular network. The magnification of the subiculum region also depicted the spatial relationship between plaques and blood vessels (Figures 8E–G and Supplementary Movie 3). The plaques in different sizes were hanged over the branches of dense capillary network. From high-resolution images of the subiculum (Figures 8H–J), we found that capillaries inside or adjacent to the plaques appeared with apparent deformations including distorted micro-vessels and abrupt ending. In addition, the plaques in the compact network of capillaries and nerve cells exhibited more holes and bulges, which lead to the excessive irregularities in surface and high level in roughness (Figure 8I and Supplementary Figures 4G–I). It should be noted that the plaques in the dense network of processes located far from the dense cell layers and appeared relatively solider and smoother, which is shown in Figures 7I–K.

## Discussion and Conclusion

In this study, we constructed an all-in-one panorama of Aβ plaques, somata, nerve processes and tracts, and blood vessels in the whole mouse brain of AD mice with a non-fluorescent labeling method. To achieve the simultaneous visualization of Aβ plaques and their surrounding brain structures, a customized image processing workflow coupled with the MOST bright-field imaging and whole-brain Nissl staining method were developed and carried out. With this technique route, we are capable to discern characteristic signals of Aβ plaques, somata, nerve processes and tracts, and blood vessels based on the difference of gray values and morphologies in the whole-brain Nissl staining dataset.

### Identification of Whole-Brain Multiple Structures Without Specific Labeling

High-content fluorescence imaging highly relies on the acquisition of multi-channel signals simultaneously. *In vivo* fluorescence labeling methods, which include injections of fluorescent dyes (Robertson et al., 2015), injection with virus expressing fluorescent proteins (Zingg et al., 2017), and transgenic mice expressing fluorescent proteins (Wessels et al., 2008), are capable of labeling several different brain structures simultaneously. For instance, Prox1-GFP/Flt1-DsRed mice with inherent fluorescence of blood and lymphatic vessels allow for direct visualization of blood and lymphatic vessels in various organs (Zhong et al., 2017). Combining with fast-developing brain-wide imaging techniques, such as light sheet microscopy (Dodt et al., 2007), serial two-photon tomography (Ragan et al., 2012), and fMOST (Gong et al., 2016), the whole-brain imaging of specimen having two or three fluorescent labels is achievable. However, imaging of specimens with three or more fluorescent labels is often complicated by the bleed-through or crossover of fluorescence emission, unless the spectral profiles of the fluorophores are very well separated (Valm et al., 2016; McRae et al., 2019).

Here, we utilized the whole-brain Nissl staining, a non-fluorescent labeling method, to achieve simultaneous displaying of whole-brain multiple structures in one sample. The conventional Nissl staining was a classic nucleic acid staining method directing at thin tissue sections and was commonly adopted to label somata with displaying Nissl body or revealing reduction in neuronal population (Nobakht et al., 2011), whereas the whole-brain Nissl staining method was modified to better label an entire brain. After the whole-brain Nissl staining, the Aβ plaques, somata, nerve processes and tracts, and blood vessels would show varying degrees of gray values and could be identified and extracted integrating with their different morphologies. Noticeably, the application of bright-field Nissl staining to display Aβ plaque has not been verified in the other studies. Our study achieved the first whole-brain distribution of Aβ plaques based on the Nissl staining. We speculated that several factors contributed to the clear presentation of Aβ plaque in this study. First, the ultrathin sections (1 μm) employed in the MOST system resulted in high axial resolution and the modification of whole-brain Nissl staining method led to enhanced contrast, both of which made it more feasible to display the Aβ plaques. Importantly, we also adjusted the MOST system with relative higher image contrast and kept the image sequence being high-quality during the long-term imaging. Besides, the capability of bright-field image processing enabled us to make 3D reconstruction on the pompon-like signal of interest, which may also contribute to the recognition and verification of the Aβ plaque in Nissl staining. In the conventional Nissl staining brain slices, the redundant information except the somata was usually ignored, even though the Aβ plaque might be displayed occasionally. Our study suggested several benefits of the application of whole-brain Nissl staining and MOST acquisition. First, analyzing the Aβ plaques in bright field images yields the possibility to measure more descriptive features such as texture and shape simultaneously. Additionally, we thus found that the Aβ plaques surrounding by nerve processes and few somata were solider and smoother, but those in compact network of capillaries and nerve cells appeared more irregular and rougher. Second, the simultaneous acquisition of multiple signals also breaks the limitation of spectral overlap of the fluorophore. To sum up, the whole-brain Nissl staining provides an alternative non-fluorescent method for labeling multiple components in brain research and offers new idea for the conventional method in new use.

### Customized Image Processing Workflow for Simultaneous Visualization

The MOST system adopted the reflected bright field imaging to generate a massive amount of data (Li et al., 2010). The processing method for the high-throughput bright-field images is more demanding than in the fluorescence case (Buggenthin et al., 2013). The bright-field images exhibited more heterogeneous gray levels and lower contrast than the fluorescent images. Specifically, the gray value of Aβ plaques exhibited inhomogeneous distribution of “W” shape and was close to that of the brain parenchyma. This characteristic gray-value distribution of Aβ plaque made it difficult to be extracted. Besides, the inhomogeneous gray level may also exist among slices over the long periods of imaging. Therefore, despite the multiple signals of different structures were provided by the whole-brain Nissl staining dataset generated from the MOST system, there are still considerable obstacles faced by simultaneous visualization of these multiple structures using direct volume rendering.

In this study, we have made attempts in developing a customized image processing workflow for the extraction and reconstruction of these multiple structures. An image optimization method for high-throughput bright-field images was first performed to enhance the image contrast and to facilitate the following segmentation. To simultaneously visualizing all of these signals in the same whole brain, we took advantage of the distinct features of different structures and utilized virtual channel splitting to extract the information of different structures, as well as ultimately performed feature fusion to provide a visual reconstruction of multiple structures. This visualization workflow could be adopted in other high-throughput bright field imaging data and will also greatly benefit the systematic understanding of the complex brain. The ability to visualize multiple structures at submicron resolution of 3D whole-brain scale, especially in the same sample, will help to clarify the underlying mechanisms of neurodegeneration.

### All-in-One Panorama of Multiple Structures in Whole Brain of AD Mice

Aβ plaques, currently believed as the earliest detectable AD biomarker, are increasingly recognized to be an intricated role interplaying with various neighboring brain structures such as neurons, glia cells, and vasculatures (Marin et al., 2016; Yuan et al., 2016; Miners et al., 2018; Greenberg et al., 2020). The previous studies employing brain-wide imaging techniques have realized the brain-wide visualization of Aβ plaques (Liebmann et al., 2016; Long et al., 2019; Whitesell et al., 2019). Besides, whole-brain imaging of the single surrounding structure such as blood vessels, cells, and neural circuits associated with Aβ plaques was also achieved easily (Meyer et al., 2008; Liebmann et al., 2016; Zhang et al., 2020). Nevertheless, a few studies have systematically correlated the other multiple surrounding structures morphology with their spatial relation to Aβ plaques, especially at the submicron level in the intact brain. Our study provided a novel and robust approach for simultaneous cross-scale reconstruction of Aβ plaques and their surrounding multiple brain structures in the whole brain. On the brain-wide scale, the spatial distribution of Aβ plaques was correlated with that of somata, blood vessels, and nerve tracts. In addition, the entorhinal cortex and adjacent subiculum regions that present the highest density of plaques and also large-sized plaques suggested that Aβ plaques might first appear in these related regions and gradually increase with age, which was consistent with the report that the 5×FAD mice developed rapid and severe amyloid pathology, with extracellular Aβ deposition beginning at 2 months in layer V of the cortex and subiculum (Oakley et al., 2006). In the local regions of cortex and hippocampus, the Aβ plaques were found distributed in the vicinity of cell layers and located in the distal end of blood vessels. Given that the 5×FAD mice were generated by the neuron-specific mouse Thy1 promoter to drive APP and PS1 transgenes overexpression (Oakley et al., 2006), it is not difficult to understand the neuronal origin for the Aβ deposits and the strong spatial distribution correlation between Aβ plaques and somata. By viewing at subcellular resolution, the bending or abrupt ending of the nerve processes, as well as distortion or fracture of micro-vessels, was visualized.

Our study has revealed the heterogeneous distribution of Aβ plaque even in the same brain regions (Figures 5H,K) and the strong correlation between Aβ plaque and soma (Figures 5, 6), which implied that investigating the plaque distribution in the context of its surrounding multi-structures might be more beneficial compared with that in different brain regions. Although further study to elucidate the underlying physiological function and mechanism behind significant structural alternation in AD is needed to carried out, our findings provided the cross-scale high-precision panorama of multiple brain structures associated with AD pathology in one same sample. Our study placed emphasis on solving problems in processing high-throughput bright field images and made attempt in developing a method for the extraction and reconstruction of multiple structures. This will facilitate a better understanding of the cerebral anatomical features under the pathological state of AD and shows extensive application prospect in drug efficacy assessment from brain-wide level.

## Methods

### Sample Preparation and Data Acquisition

The 6-month-old 5×FAD mice and WT littermates were provided and used in this work. The 5×FAD mice carry three mutations of the human amyloid precursor gene (Swedish-K670N/M671L, Florida-I716V, and London-V717I) and two mutations of the human presenilin-1 gene (M146L and L286V) and recapitulate many AD-related phenotypes and have a relatively early and aggressive presentation (Oakley et al., 2006). The transgenic mice were obtained from The Jackson Laboratory (stock no: 34840-JAX), and the corresponding WT control mice were littermates of the AD mice. All the animal procedures were performed in accordance with the National Institutes of Health Guide for the Care and Use of Laboratory Animals, under the protocols approved by and strictly following the guidelines of the Institutional Animal Care and Use Committee (IACUC). The whole-brain staining method followed the previous description (Wu et al., 2014). In brief, the entire intact brains were carefully removed and post-fixed after cardiac perfusion. Then, the brains were stained with 2.5% thionine (Sigma-Aldrich, 861340) solution for at least 12 days. The stained brains were subsequently dehydrated with a graded series of ethanol and acetone solutions. Next, the dehydrated brains were immersed and then embedded in Spurr resin (Sigma-Aldrich, EM0300) to be suitable for the MOST system. During the long-term imaging, we adjusted the MOST system with high image contrast and kept the image sequence being high quality.

### Image Preprocessing and Optimization

The raw images were preprocessed for seamless image stitching and luminance nonuniformity correction based on a previous preprocessing program (Ding et al., 2013). Then, an image optimization method was performed to further improve background uniformity, periodic noise, and to enhance the image contrast of the preprocessed coronal images. The optimization was implemented through steps including background correction, noise reduction, and contrast enhancement (Zhang et al., 2019). The enhanced contrast of the optimized coronal images significantly improved the convenience of information extraction and grayscale rendering.

### Verification of Aβ Plaque

The brains were first prepared by the whole-brain Nissl staining method without resin embedding and then followed by Aβ-specific staining of paraffin sections. Briefly, the mouse brains were first put into 2.5% thionine staining solution for 12-day whole-brain Nissl staining. After the staining completed, the mouse brains were immersed in 50% ethanol for 2 h and then in 70% ethanol for 6 days. The samples were subsequently subjected to gradient dehydration and paraffin infiltration: 95% ethanol for 2 h; two steps of 100% ethanol for 1 h each; mixed volume of xylene and absolute ethanol for 20 min; two steps of xylene for 20 min each; dipping wax (56–58°C) for 2 h; embedding; slicing. After obtaining the paraffin sections of 5×FAD mouse brains, anti-Aβ antibody ab2454 (CST, 2454s) and Alexa fluor 546-conjugated secondary antibody were incubated on the same sections. Subsequently, the sections were stained with 1% ThS solution and mounted with a coverslip. Finally, the sections were imaged with confocal microscopy.

### Simultaneous Visualization of Multiple Brain Components

The optimized high-quality coronal images of 5×FAD mice were directly used to extract different brain structure information in different gray levels. The enhanced contrast of the optimized images significantly improves the convenience of recognition and extraction of Aβ plaques, blood vessels, somata, neuronal processes, and nerve tracts. In this paper, the structural information was classified into three categories, namely, blood vessels, plaques, and neural structures, which were extracted by virtual channel splitting individually (refer to Supplementary Figure 1). The source code is available and provided in the Supplementary Material. The code includes scripting commands using Tool Command Language (Tcl) with Amira-specific extensions. It allows users to automate certain processes and to create scripts for managing routine tasks or for presenting demos in the Amira software (Amira 2019.2). In brief, before executing the Tcl code, the data directory path must be set in the scripts. The location where should input the data directory path had been annotated in the word file “Scripts for Virtual channel splitting.” Then, copy all the scripts to a TXT file and change the file extension as “.hx.” Next, drag and drop the “.hx” file onto the Amira console to run the Tcl code in the Amira software. It should be noted that the parameters set in the scripts are suitable for the dataset generated by MOST and optimized by our method (Zhang et al., 2019). When handling with the other datasets, the parameters should be correspondingly modified.

Subsequently, high-quality rendering of these different structures was integrated through feature fusion to accomplish synchronous visualization of multiple structures in one sample. Iso-surface rendering and direct volume rendering were employed to achieve high-quality rendering of brain structures using Amira software. Iso-surface rendering intends to visualize an iso-surface in the 3D data field (Bosma et al., 1998) and has several well-recognized advantages in efficiency and accuracy. Direct volume rendering represents one of the most widely used methods for 3D visualization of tomographic images. In volume rendering, image projections are constructed by simulating the absorption and emission of light in an image stack along each ray path to the eye. The blood vessels were filtered out by region growth algorithm and then were visualized using iso-surface rendering. The somata, nerve processes, and tracts were reconstructed using direct volume rendering in Amira software. The pompon-like structures were visualized by high-precision real-time rendering after feature enhancement.

We first reconstructed Aβ plaques, blood vessels, somata, and nerve tracts at the whole brain scale and then reconstructed these various brain structures on the typical coronal plane (400 μm in thickness) of the striatum and hippocampus. The high-precision reconstruction of somata, blood vessels, and Aβ plaques in the typical cortical and hippocampal regions was also conducted. The impact of Aβ plaque on its surrounding nerve processes and capillaries was investigated by visualizing nerve processes or blood vessels associated with Aβ plaques. A 3D heatmap of plaque density was performed across the whole brain. The density of plaques was coded by a color scale and deep blue represents the lowest density. The plaque number and volume in the entorhinal cortex and its adjacent cortex, as well as in the hippocampal subiculum and CA3 regions, were calculated for three voxel blocks (0.5 × 0.5 × 0.5 mm^3^, one blocks per mouse) from the similar anatomical position of the brain of three mice. Besides, the soma number and plaque number for three 5×FAD mice were calculated in different cortical depths that were normalized by the whole cortical size of each sample. The soma number for WT mice was also calculated in different cortical depths to evaluate the normal distribution correlation between somata and blood vessels.

## Data Availability Statement

The original contributions presented in the study are included in the article/Supplementary Material, further inquiries can be directed to the corresponding author/s. The source code is provided in the Supplementary Material (refer to Scripts for Virtual channel splitting).

## Ethics Statement

The animal study was reviewed and approved by Institutional Animal Care and Use Committee of Shanghai Institute of Materia Medica.

## Author Contributions

HJ, ZG, and HZ conceived and designed the research. JZ performed the animal experiments. XZ performed the sample preparation, data acquisition, and image preprocessing. XZ and JZ performed the validation of Aβ plaque. XY performed the image optimization, visualization, and quantitative analysis. HJ, ZG, HZ, XY, XZ, and JZ wrote the manuscript. All authors analyzed the data. All authors contributed to the article and approved the submitted version.

## Funding

This work was supported by the National Science Fund for Distinguished Young Scholars (81825021 to ZG), Youth Innovation Promotion Association CAS (2018323 to XY), Shanghai Municipal Science and Technology Major Project (2018SHZDZX05 to HJ and ZG), China Postdoctoral Science Foundation (2021M703344 to XZ), and Shanghai Super Postdoctoral Incentive Program (2021431 to XZ).

## Conflict of Interest

The authors declare that the research was conducted in the absence of any commercial or financial relationships that could be construed as a potential conflict of interest.

## Publisher's Note

All claims expressed in this article are solely those of the authors and do not necessarily represent those of their affiliated organizations, or those of the publisher, the editors and the reviewers. Any product that may be evaluated in this article, or claim that may be made by its manufacturer, is not guaranteed or endorsed by the publisher.

## References

[B1] Adalbert R. Nogradi A. Babetto E. Janeckova L. Walker S. A. Kerschensteiner M.. (2009). Severely dystrophic axons at amyloid plaques remain continuous and connected to viable cell bodies. Brain 132 (Pt 2), 402–416. 10.1093/brain/awn312 19059977

[B2] Apostolova L. G. Green A. E. Babakchanian S. Hwang K. S. Chou Y. Y. Toga A. W.. (2012). Hippocampal atrophy and ventricular enlargement in normal aging, mild cognitive impairment (MCI), Alzheimer Disease. Alzheimer Dis. Assoc. Disord. 26, 17–27. 10.1097/WAD.0b013e3182163b62 22343374 PMC3286134

[B3] Appaix F. Girod S. Boisseau S. Romer J. Vial J. C. Albrieux M.. (2012). Specific in vivo staining of astrocytes in the whole brain after intravenous injection of sulforhodamine dyes. PLoS ONE 7, e35169. 10.1371/journal.pone.0035169 22509398 PMC3324425

[B4] Bacskai B. J. Hickey G. A. Skoch J. Kajdasz S. T. Wang Y. Huang G. F.. (2003). Four-dimensional multiphoton imaging of brain entry, amyloid binding, and clearance of an amyloid-beta ligand in transgenic mice. Proc. Natl. Acad. Sci. U.S.A. 100, 12462–12467. 10.1073/pnas.2034101100 14517353 PMC218780

[B5] Bittner T. Burgold S. Dorostkar M. M. Fuhrmann M. Wegenast-Braun B. M. Schmidt B.. (2012). Amyloid plaque formation precedes dendritic spine loss. Acta Neuropathol. 124, 797–807. 10.1007/s00401-012-1047-8 22993126 PMC3508278

[B6] Bolmont T. Bouwens A. Pache C. Dimitrov M. Berclaz C. Villiger M.. (2012). Label-free imaging of cerebral beta-amyloidosis with extended-focus optical coherence microscopy. J. Neurosci. 32, 14548–14556. 10.1523/JNEUROSCI.0925-12.2012 23077040 PMC6621450

[B7] Bosma M. Jaap S. Steven J. (1998). “Storage lobregt retrieval for image, video databases,” in Iso-Surface Volume Rendering (San Diego, CA).

[B8] Bozzali M. Falini A. Franceschi M. Cercignani M. Zuffi M. Scotti G.. (2002). White matter damage in Alzheimer's disease assessed in vivo using diffusion tensor magnetic resonance imaging. J. Neurol Neurosurg. Psychiatry 72, 742–746. 10.1136/jnnp.72.6.742 12023417 PMC1737921

[B9] Buggenthin F. Carsten M. Michael S. Philipp Hoppe S. Oliver H. Timm S.. (2013). An automatic method for robust and fast cell detection in bright field images from high-throughput microscopy. BMC Bioinformatics 14, 297. 10.1186/1471-2105-14-297 24090363 PMC3850979

[B10] Daigle T. L. Madisen L. Hage T. A. Valley M. T. Knoblich U. Larsen R. S.. (2018). A suite of transgenic driver and reporter mouse lines with enhanced brain-cell-type targeting and functionality. Cell 174, 465.e22–480.e22. 10.1016/j.cell.2018.06.035 30007418 PMC6086366

[B11] Delafontaine-Martel P. Lefebvre J. Tardif P. L. Levy B. I. Pouliot P. Lesage F. (2018). Whole brain vascular imaging in a mouse model of Alzheimer's disease with two-photon microscopy. J. Biomed. Opt. 23, 1–10. 10.1117/1.JBO.23.7.076501 29998647

[B12] Dickson T. C. Vickers J. C. (2001). The morphological phenotype of beta-amyloid plaques and associated neuritic changes in Alzheimer's disease. Neuroscience 105, 99–107. 10.1016/S0306-4522(01)00169-5 11483304

[B13] Ding W. Wu L. i. A Yang J. Meng Z. Wang Y.. (2013). Automatic macroscopic density artefact removal in a Nissl-stained microscopic atlas of whole mouse brain. J. Microsc. 251, 168–177. 10.1111/jmi.12058 23781931

[B14] Dodt H. U. Leischner U. Schierloh A. Jahrling N. Mauch C. P. Deininger K.. (2007). Ultramicroscopy: three-dimensional visualization of neuronal networks in the whole mouse brain. Nat. Methods 4, 331–336. 10.1038/nmeth1036 17384643

[B15] Dorr A. Sled J. G. Kabani N. (2007). Three-dimensional cerebral vasculature of the CBA mouse brain: a magnetic resonance imaging and micro computed tomography study. Neuroimage 35, 1409–1423. 10.1016/j.neuroimage.2006.12.040 17369055

[B16] Enzlein T. Cordes J. Munteanu B. Michno W. Serneels L. Strooper D. C.. (2020). Computational analysis of alzheimer amyloid plaque composition in 2D-and elastically reconstructed 3D-MALDI MS images. Anal. Chem. 92, 14484–14493. 10.1021/acs.analchem.0c02585 33138378

[B17] Fakhoury M. (2018). Microglia and astrocytes in Alzheimer's disease: implications for therapy. Curr. Neuropharmacol. 16, 508–518. 10.2174/1570159X15666170720095240 28730967 PMC5997862

[B18] Fu H. Tu P. Zhao L. Liu D. A. I. J. Cui B. M. (2016). Amyloid-beta deposits target efficient near-infrared fluorescent probes: synthesis, *in vitro* evaluation, and *in vivo* imaging. Anal. Chem. 88, 1944–1950. 10.1021/acs.analchem.5b04441 26717442

[B19] Gang Y. Liu X. Wang X. Zhang Q. Zhou H. Chen R.. (2017). Plastic embedding immunolabeled large-volume samples for three-dimensional high-resolution imaging. Biomed. Opt. Express 8, 3583–3596. 10.1364/BOE.8.003583 28856037 PMC5560827

[B20] Gao R. Asano S. M. Upadhyayula S. Pisarev I. Milkie D. E. Liu T. L.. (2019). Cortical column and whole-brain imaging with molecular contrast and nanoscale resolution. Science 363, 6424. 10.1126/science.aau8302 30655415 PMC6481610

[B21] Giovanna D. I. Tibo A. P. Silvestri A. Mullenbroich L. Costantini M. C. Allegra Mascaro I.. (2018). Whole-brain vasculature reconstruction at the single capillary level. Sci. Rep. 8, 12573. 10.1038/s41598-018-30533-3 30135559 PMC6105658

[B22] Gong H. Xu D. Yuan J. Li X. Guo C. Peng J.. (2016). High-throughput dual-colour precision imaging for brain-wide connectome with cytoarchitectonic landmarks at the cellular level. Nat. Commun. 7, 12142. 10.1038/ncomms12142 27374071 PMC4932192

[B23] Greenberg S. M. Bacskai B. J. Hernandez-Guillamon M. Pruzin J. Sperling R. van Veluw J. S. (2020). Cerebral amyloid angiopathy and Alzheimer disease - one peptide, two pathways. Nat. Rev. Neurol. 16, 30–42. 10.1038/s41582-019-0281-2 31827267 PMC7268202

[B24] Gurol M. E. Becker J. A. Fotiadis P. Riley G. Schwab K. Johnson K. A.. (2016). Florbetapir-PET to diagnose cerebral amyloid angiopathy: a prospective study. Neurology 87, 2043–2049. 10.1212/WNL.0000000000003197 27605173 PMC5109947

[B25] Ji M. Arbel L. Zhang M. Freudiger L. Hou C. W. Lin S. S.. (2018). Label-free imaging of amyloid plaques in Alzheimer's disease with stimulated Raman scattering microscopy. Sci. Adv. 4, eaat7715. 10.1126/sciadv.aat7715 30456301 PMC6239428

[B26] Jing D. Zhang S. Luo W. Gao X. Men Y. Ma C.. (2018). Tissue clearing of both hard and soft tissue organs with the PEGASOS method. Cell Res. 28, 803–818. 10.1038/s41422-018-0049-z 29844583 PMC6082844

[B27] Johansson P. K. Koelsch P. (2017). Label-free imaging of amyloids using their intrinsic linear and nonlinear optical properties. Biomed. Opt. Express 8, 743–756. 10.1364/BOE.8.000743 28270981 PMC5330564

[B28] Kadowaki H. Nishitoh H. Urano F. Sadamitsu C. Matsuzawa A. Takeda K.. (2005). Amyloid beta induces neuronal cell death through ROS-mediated ASK1 activation. Cell Death Differ. 12, 19–24. 10.1038/sj.cdd.4401528 15592360

[B29] Khouri K. Xie D. F. Crouzet C. Bahani A. W. Cribbs D. H. Fisher M. J.. (2021). Simple methodology to visualize whole-brain microvasculature in three dimensions. Neurophotonics 8, 025004. 10.1117/1.NPh.8.2.025004 33884280 PMC8056070

[B30] Kimbrough I. F. Robel S. Roberson E. D. Sontheimer H. (2015). Vascular amyloidosis impairs the gliovascular unit in a mouse model of Alzheimer's disease. Brain 138 (Pt 12), 3716–3733. 10.1093/brain/awv327 26598495 PMC5006220

[B31] Kirst C. Skriabine S. Vieites-Prado A. Topilko T. Bertin P. Gerschenfeld G.. (2020). Mapping the fine-scale organization and plasticity of the brain vasculature. Cell 180, 780–795. e25. 10.1016/j.cell.2020.01.028 32059781

[B32] Klunk W. E. Bacskai B. J. Mathis C. A. Kajdasz S. T. McLellan M. E. Frosch M. P.. (2002). Imaging Abeta plaques in living transgenic mice with multiphoton microscopy and methoxy-X04, a systemically administered Congo red derivative. J. Neuropathol. Exp. Neurol. 61, 797–805. 10.1093/jnen/61.9.797 12230326

[B33] Konno A. Matsumoto N. Tomono Y. Okazaki S. (2020). Pathological application of carbocyanine dye-based multicolour imaging of vasculature and associated structures. Sci. Rep. 10, 12613. 10.1038/s41598-020-69394-0 32724051 PMC7387484

[B34] Leiva-Salinas C. Jiang B. Wintermark M. (2018). Computed tomography, computed tomography angiography, and perfusion computed tomography evaluation of acute ischemic stroke. Neuroimaging Clin. N. Am. 28, 565–572. 10.1016/j.nic.2018.06.002 30322593

[B35] Li A. Gong H. Zhang H. Wang B. Yan Q. Wu C.. (2010). Micro-optical sectioning tomography to obtain a high-resolution atlas of the mouse brain. Science 330, 1404–1408. 10.1126/science.1191776 21051596

[B36] Liebmann T. Renier N. Bettayeb K. Greengard P. Tessier-Lavigne M. Flajolet M. (2016). Three-dimensional study of Alzheimer's disease hallmarks using the iDISCO clearing method. Cell Rep. 16, 1138–1152. 10.1016/j.celrep.2016.06.060 27425620 PMC5040352

[B37] Liu Z. Condello C. Schain A. Harb R. Grutzendler J. (2010), CX3CR.1 in microglia regulates brain amyloid deposition through selective protofibrillar amyloid-beta phagocytosis. J. Neurosci. 30, 17091–17101. 10.1523/JNEUROSCI.4403-10.2010 21159979 PMC3077120

[B38] Long B. Xiangning L. Jianping Z. Siqi C. Wenwei L. Qiuyuan Z.. (2019). Three-dimensional quantitative analysis of amyloid plaques in the whole brain with high voxel resolution. Scientia Sinica Vitae 49, 140–150. 10.1360/N052019-00001

[B39] Luo Y. Wang A. Liu M. Lei T. Zhang X. Gao Z.. (2017). Label-free brainwide visualization of senile plaque using cryo-micro-optical sectioning tomography. Opt. Lett. 42, 4247–4250. 10.1364/OL.42.004247 29088144

[B40] Mano T. Albanese A. Dodt H. U. Erturk A. Gradinaru V. Treweek J. B.. (2018). Whole-brain analysis of cells and circuits by tissue clearing and light-sheet microscopy. J. Neurosci. 38, 9330–9337. 10.1523/JNEUROSCI.1677-18.2018 30381424 PMC6706004

[B41] Marin M. A. Ziburkus J. Jankowsky J. Rasband M. N. (2016). Amyloid-beta plaques disrupt axon initial segments. Exp. Neurol. 281, 93–98. 10.1016/j.expneurol.2016.04.018 27109181 PMC4877279

[B42] McRae T. D. Oleksyn D. Miller J. Gao Y. R. (2019). Robust blind spectral unmixing for fluorescence microscopy using unsupervised learning. PLoS ONE 14, e0225410. 10.1371/journal.pone.0225410 31790435 PMC6886781

[B43] Meyer E. P. Ulmann-Schuler A. Staufenbiel M. Krucker T. (2008). Altered morphology and 3D architecture of brain vasculature in a mouse model for Alzheimer's disease. Proc. Natl. Acad. Sci. U.S.A. 105, 3587–3592. 10.1073/pnas.0709788105 18305170 PMC2265182

[B44] Miners J. S. Schulz I. Love S. (2018). Differing associations between Abeta accumulation, hypoperfusion, blood-brain barrier dysfunction and loss of PDGFRB pericyte marker in the precuneus and parietal white matter in Alzheimer's disease. J. Cereb. Blood Flow Metab. 38, 103–115. 10.1177/0271678X17690761 28151041 PMC5757436

[B45] Miyawaki T. Morikawa S. Susaki E. A. Nakashima A. Takeuchi H. Yamaguchi S.. (2020). Visualization and molecular characterization of whole-brain vascular networks with capillary resolution. Nat. Commun. 11, 1104. 10.1038/s41467-020-14786-z 32107377 PMC7046771

[B46] Murphy M. P. LeVine H. III. (2010). Alzheimer's disease and the amyloid-beta peptide. J. Alzheimers Dis. 19, 311–323. 10.3233/JAD-2010-1221 20061647 PMC2813509

[B47] Nobakht M. Hoseini S. M. Mortazavi P. Sohrabi I. Esmailzade B. Rahbar Rooshandel N.. (2011). Neuropathological changes in brain cortex and hippocampus in a rat model of Alzheimer's disease. Iran. Biomed. J. 15, 51–58. 21725500 PMC3639737

[B48] Oakley H. Cole S. L. Logan S. Maus E. Shao P. Craft J.. (2006). Intraneuronal beta-amyloid aggregates, neurodegeneration, and neuron loss in transgenic mice with five familial Alzheimer's disease mutations: potential factors in amyloid plaque formation. J. Neurosci. 26, 10129–10140. 10.1523/JNEUROSCI.1202-06.2006 17021169 PMC6674618

[B49] Pini L. Pievani M. Bocchetta M. Altomare D. Bosco P. Cavedo E.. (2016). Brain atrophy in Alzheimer's disease and aging. Ageing Res. Rev. 30, 25–48. 10.1016/j.arr.2016.01.002 26827786

[B50] Ragan T. Kadiri L. R. Venkataraju K. U. Bahlmann K. Sutin J. Taranda J.. (2012). Serial two-photon tomography for automated ex vivo mouse brain imaging. Nat. Methods 9, 255–258. 10.1038/nmeth.1854 22245809 PMC3297424

[B51] Robertson R. T. Levine S. T. Haynes S. M. Gutierrez P. Baratta J. L. Tan Z.. (2015). Use of labeled tomato lectin for imaging vasculature structures. Histochem. Cell Biol. 143, 225–234. 10.1007/s00418-014-1301-3 25534591

[B52] Serrano-Pozo A. Frosch M. P. Masliah E. Hyman B. T. (2011). Neuropathological alterations in Alzheimer disease. Cold Spring Harb. Perspect. Med. 1, a006189. 10.1101/cshperspect.a006189 22229116 PMC3234452

[B53] Valm A. M. Oldenbourg R. Borisy G. G. (2016). Multiplexed spectral imaging of 120 different fluorescent labels. PLoS ONE 11, e0158495. 10.1371/journal.pone.0158495 27391327 PMC4938436

[B54] Wessels J. T. Hoffman R. M. Wouters F. S. (2008). The use of transgenic fluorescent mouse strains, fluorescent protein coding vectors, and innovative imaging techniques in the life sciences. Cytometry A 73, 490–491. 10.1002/cyto.a.20548 18307256

[B55] Whitesell J. D. Buckley A. R. Knox J. E. Kuan L. Graddis N. Pelos A.. (2019). Whole brain imaging reveals distinct spatial patterns of amyloid beta deposition in three mouse models of Alzheimer's disease. J. Comp. Neurol. 527, 2122–2145. 10.1002/cne.24555 30311654 PMC8026112

[B56] Wu J. He Y. Yang Z. Guo C. Luo Q. Zhou W.. (2014). 3D BrainCV: simultaneous visualization and analysis of cells and capillaries in a whole mouse brain with one-micron voxel resolution. Neuroimage 87, 199–208. 10.1016/j.neuroimage.2013.10.036 24185025

[B57] Xiong B. Li A. Lou Y. Chen S. Long B. Peng J.. (2017). Precise cerebral vascular atlas in stereotaxic coordinates of whole mouse brain. Front. Neuroanat. 11, 128. 10.3389/fnana.2017.00128 29311856 PMC5742197

[B58] Youmans K. L. Tai L. M. Kanekiyo T. Stine W. B. Jr. Michon S. C.. (2012). Intraneuronal Aβ detection in 5xFAD mice by a new Aβ-specific antibody. Mol. Neurodegener. 7, 8. 10.1186/1750-1326-7-8 22423893 PMC3355009

[B59] Yuan J. Gong H. Li A. Li X. Chen S. Zeng S.. (2015). Visible rodent brain-wide networks at single-neuron resolution. Front. Neuroanat. 9, 70. 10.3389/fnana.2015.00070 26074784 PMC4446545

[B60] Yuan P. Condello C. Keene C. D. Wang Y. Bird T. D. Paul S. M.. (2016). TREM2 haplodeficiency in mice and humans impairs the microglia barrier function leading to decreased amyloid compaction and severe axonal dystrophy. Neuron 90, 724–739. 10.1016/j.neuron.2016.05.003 27196974 PMC4898967

[B61] Zhang J. Long B. Li A. Sun J. Tian J. Luo T.. (2020). Whole-brain three-dimensional profiling reveals brain region specific axon vulnerability in 5xFAD mouse model. Front. Neuroanat. 14, 608177. 10.3389/fnana.2020.608177 33324177 PMC7726261

[B62] Zhang X. Xianzhen Y. Jingjing Z. Anan L. Hui G. Qingming L.. (2019). High-resolution mapping of brain vasculature and its impairment in the hippocampus of Alzheimer's disease mice. National Science Review 6, 1223–1238. 10.1093/nsr/nwz124 34692000 PMC8291402

[B63] Zhong W. Gao X. Wang S. Han K. Ema M. Adams S.. (2017). Prox1-GFP/Flt1-DsRed transgenic mice: an animal model for simultaneous live imaging of angiogenesis and lymphangiogenesis. Angiogenesis 20, 581–598. 10.1007/s10456-017-9572-7 28795242 PMC5782821

[B64] Zingg B. Chou X. L. Zhang Z. G. Mesik L. Liang F. Tao H. W.. (2017). AAV-Mediated anterograde transsynaptic tagging: mapping corticocollicular input-defined neural pathways for defense behaviors. Neuron 93, 33–47. 10.1016/j.neuron.2016.11.045 27989459 PMC5538794

