# Supplementary material for: High-Resolution Digital Panorama of Multiple Structures in Whole Brain of Alzheimer's Disease Mice
Source: Front Neurosci. 2022 Apr 19;16:870520. doi: 10.3389/fnins.2022.870520 (PMC9067162; doi:10.3389/fnins.2022.870520)
Supplement: Supplementary file 1 [file Data_Sheet_1.docx]

Supplementary Material

Supplementary Figure 1 - 4

Supplementary Movie 1 - 3

Scripts for Virtual channel splitting


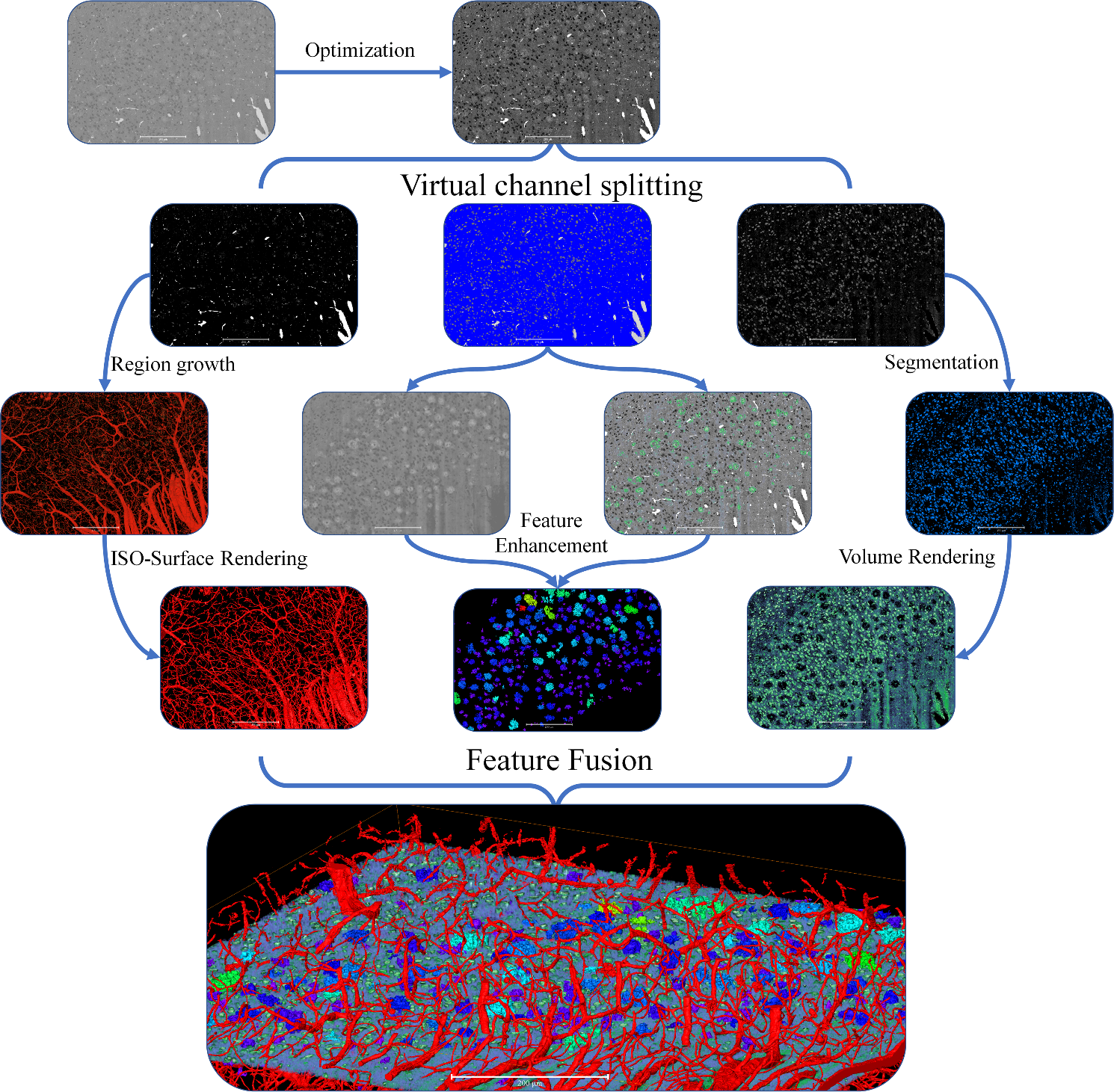


**Supplementary Figure 1.** Procedure of customized image processing workflow for simultaneous reconstruction.

**
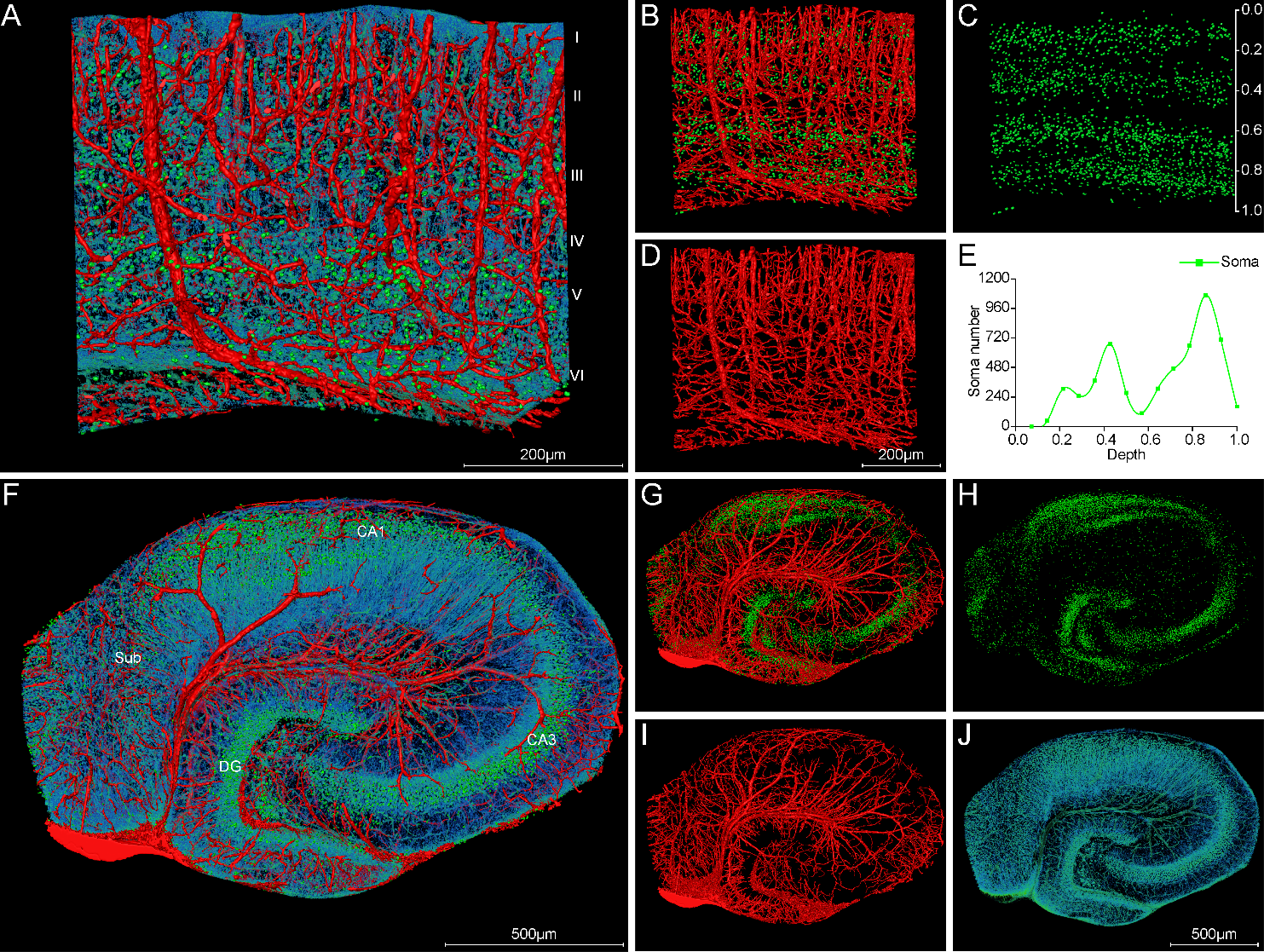
 Supplementary Figure 2.** Visual reconstruction of somata and vessels in age-matched WT mice.

(A-D) Simultaneous visualization of multiple signals in the cortex of WT mouse with different structures coded by different pseudo-colors. (A) Representative coronal view of local cortical region showing the spatial relationship among somata, vessels, and neuronal processes (light blue). (B-D) The same sections as shown in A were displayed with somata, and vessels to demonstrate the spatial relationship between them. (E) Quantitative analysis of the number of somata with cortical depth changing based on images of 50-μm-thick coronal slices similar to that shown in C. The y axis denoted the number of somata. The x axis represented cortical depth which was normalized by whole cortical size and was corresponding to the coordinate in C. (F-J) Simultaneous visualization of multiple signals in the hippocampus of WT mouse with different structures coded by different pseudo-colors. (F) Representative sectional views perpendicular to the longitudinal hippocampal axis showing the spatial relationship among somata, vessels, and neuronal processes (light blue). (G-J) The same sections as shown in F were displayed with somata, vessels, and neuronal processes to demonstrate the spatial relationship among them.


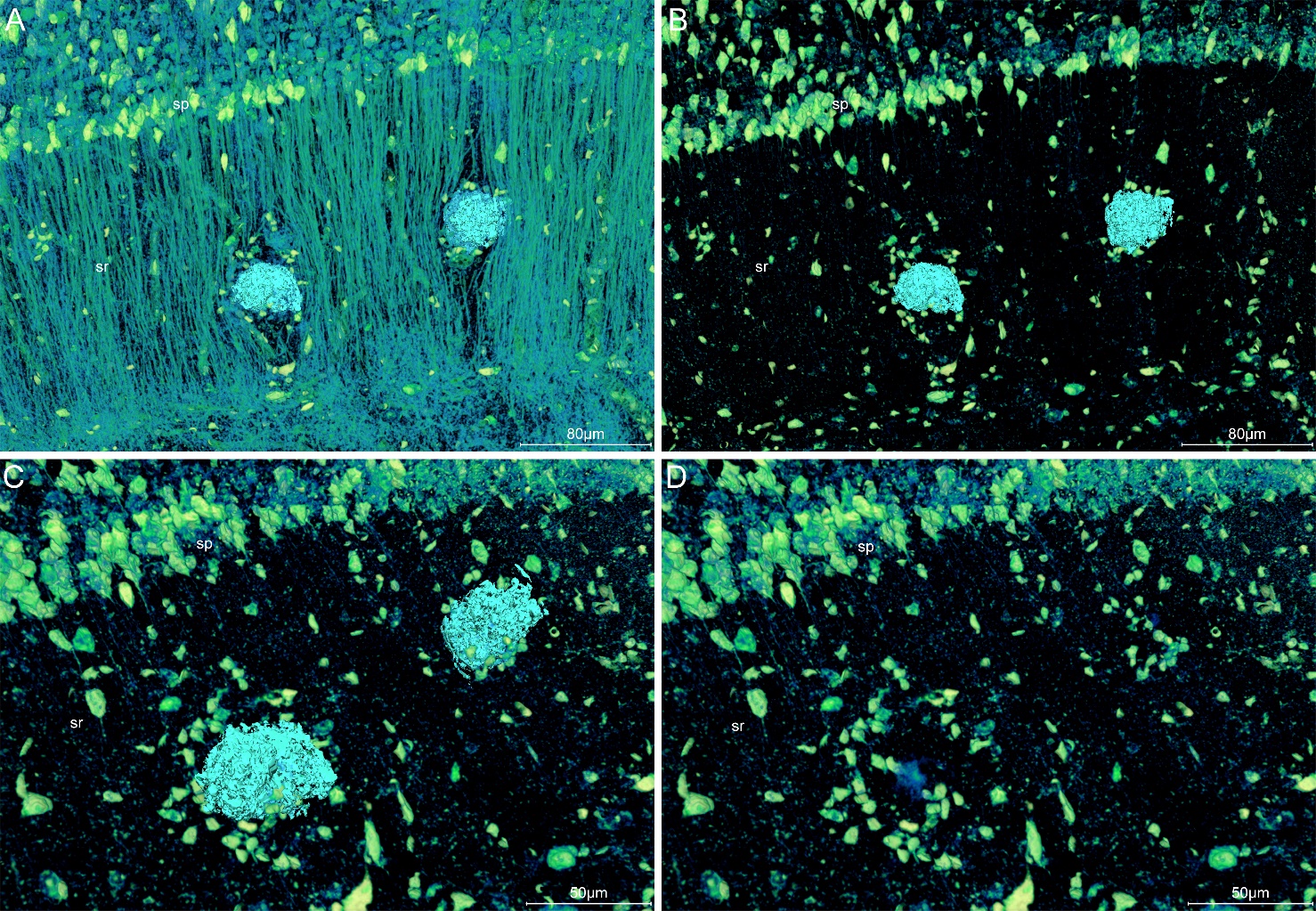
**Supplementary Figure 3.** Smaller somata clustering around Aβ plaques.

(A-C) The same voxel blocks as shown in Fig. 7I were displayed with plaques, somata, and neuronal processes to illustrate the plaque-associated smaller somata surrounding Aβ plaques. The somata was in a range of green color coded by gray value and the smaller somata were displayed in bright green.


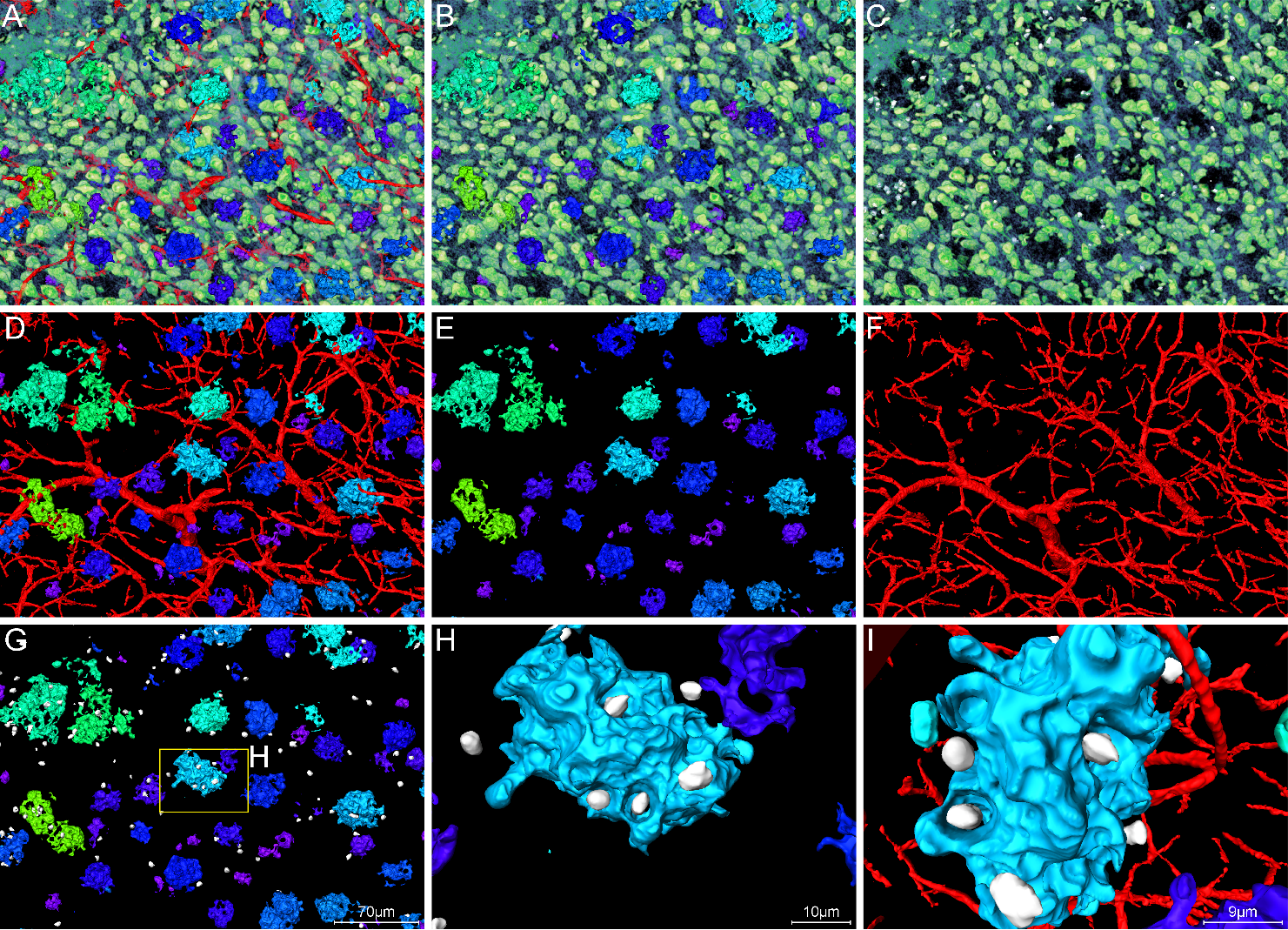
 **Supplementary Figure 4.** High-resolution demonstration of Aβ plaques and adjacent somata, vessels in cortical area.

(A-F) The Aβ plaques interspersing among compact network of capillaries and nerve cells led to vacancy in nerve tissue and disruption of capillaries. The cell processes were in light blue. The small-sized smaller somata were in white. The capillaries were in red. The irregular plaques were in different colors (e.g. magenta, green and dark blue). (G-I) The plaque-associated smaller somata (in white) in or around Aβ plaques. (H) The boxed area in G was shown in a higher magnification. (I) A different view of the same plaque in H with the addition of blood vessels.

**Supplementary Movie 1.** Whole-brain visualization of Aβ plaque distributions.

**Supplementary Movie 2.** 3D heatmap of whole-brain plaque density distribution.

**Supplementary Movie 3.** Simultaneous visualization of Aβ plaques and vasculatures in subiculum region.

**Scripts for Virtual channel splitting:**

The code includes scripting commands using Tool Command Language (Tcl) with Amira-specific extensions. It allows users to automate certain processes and to create scripts for managing routine tasks or for presenting demos in the Amira software (Amira 2019.2). The related word file named “Scripts for Virtual channel splitting” has been uploaded in Supplementary Material.
