# Supplementary material for: High-Resolution Digital Panorama of Multiple Structures in Whole Brain of Alzheimer's Disease Mice
Source: Front Neurosci. 2022 Apr 19;16:870520. doi: 10.3389/fnins.2022.870520 (PMC9067162; doi:10.3389/fnins.2022.870520)
Supplement: Supplementary file 5 [file Data_Sheet_2.docx]

# Amira Project 201920

# Amira

# Generated by Amira 2019.2

# Create viewers

viewer setVertical 0

viewer 0 setTransparencyType 6

viewer 0 setAutoRedraw 0

viewer 0 show

mainWindow show

_units setUnitsManagementMode 0

set hideNewModules 1

[ load ${AMIRA_ROOT}/data/colormaps/temperature.icol ] setLabel "temperature.icol"

"temperature.icol" setIconPosition 0 0

"temperature.icol" setNoRemoveAll 1

"temperature.icol" fire

"temperature.icol" setMinMax 38 44

"temperature.icol" flags setValue 1

"temperature.icol" shift setMinMax -1 1

"temperature.icol" shift setButtons 0

"temperature.icol" shift setEditButton 1

"temperature.icol" shift setIncrement 0.133333

"temperature.icol" shift setValue 0

"temperature.icol" shift setSubMinMax -1 1

"temperature.icol" scale setMinMax 0 1

"temperature.icol" scale setButtons 0

"temperature.icol" scale setEditButton 1

"temperature.icol" scale setIncrement 0.1

"temperature.icol" scale setValue 1

"temperature.icol" scale setSubMinMax 0 1

"temperature.icol" fire

"temperature.icol" setViewerMask 16383

set hideNewModules 1

[ load ${AMIRA_ROOT}/data/colormaps/grayScale.am ] setLabel "grayScale.am"

"grayScale.am" setIconPosition 0 0

"grayScale.am" setNoRemoveAll 1

"grayScale.am" setVar "CustomHelp" {HxColormap256}

"grayScale.am" fire

"grayScale.am" setMinMax 0 255

"grayScale.am" flags setValue 1

"grayScale.am" shift setMinMax -1 1

"grayScale.am" shift setButtons 0

"grayScale.am" shift setEditButton 1

"grayScale.am" shift setIncrement 0.133333

"grayScale.am" shift setValue 0

"grayScale.am" shift setSubMinMax -1 1

"grayScale.am" scale setMinMax 0 1

"grayScale.am" scale setButtons 0

"grayScale.am" scale setEditButton 1

"grayScale.am" scale setIncrement 0.1

"grayScale.am" scale setValue 1

"grayScale.am" scale setSubMinMax 0 1

"grayScale.am" fire

"grayScale.am" setViewerMask 16383

set hideNewModules 1

[ load ${AMIRA_ROOT}/data/colormaps/physics.icol ] setLabel "physics.icol"

"physics.icol" setIconPosition 0 0

"physics.icol" setNoRemoveAll 1

"physics.icol" fire

"physics.icol" setMinMax 0 1

"physics.icol" flags setValue 1

"physics.icol" shift setMinMax -1 1

"physics.icol" shift setButtons 0

"physics.icol" shift setEditButton 1

"physics.icol" shift setIncrement 0.133333

"physics.icol" shift setValue 0

"physics.icol" shift setSubMinMax -1 1

"physics.icol" scale setMinMax 0 1

"physics.icol" scale setButtons 0

"physics.icol" scale setEditButton 1

"physics.icol" scale setIncrement 0.1

"physics.icol" scale setValue 1

"physics.icol" scale setSubMinMax 0 1

"physics.icol" fire

"physics.icol" setViewerMask 16383

set hideNewModules 1

[ load ${AMIRA_ROOT}/data/colormaps/volrenGreen.col ] setLabel "volrenGreen.col"

"volrenGreen.col" setIconPosition 0 0

"volrenGreen.col" setNoRemoveAll 1

"volrenGreen.col" setVar "CustomHelp" {HxColormap256}

"volrenGreen.col" fire

"volrenGreen.col" setMinMax 10 200

"volrenGreen.col" flags setValue 1

"volrenGreen.col" shift setMinMax -1 1

"volrenGreen.col" shift setButtons 0

"volrenGreen.col" shift setEditButton 1

"volrenGreen.col" shift setIncrement 0.133333

"volrenGreen.col" shift setValue 0

"volrenGreen.col" shift setSubMinMax -1 1

"volrenGreen.col" scale setMinMax 0 1

"volrenGreen.col" scale setButtons 0

"volrenGreen.col" scale setEditButton 1

"volrenGreen.col" scale setIncrement 0.1

"volrenGreen.col" scale setValue 1

"volrenGreen.col" scale setSubMinMax 0 1

"volrenGreen.col" fire

"volrenGreen.col" setViewerMask 16383

set hideNewModules 1

[ load ${AMIRA_ROOT}/data/colormaps/labels.am ] setLabel "labels.am"

"labels.am" setIconPosition 20 1491

"labels.am" setNoRemoveAll 1

"labels.am" setVar "CustomHelp" {HxColormap256}

"labels.am" fire

"labels.am" setMinMax 1 8

"labels.am" flags setValue 1

"labels.am" shift setMinMax -1 1

"labels.am" shift setButtons 0

"labels.am" shift setEditButton 1

"labels.am" shift setIncrement 0.133333

"labels.am" shift setValue 0

"labels.am" shift setSubMinMax -1 1

"labels.am" scale setMinMax 0 1

"labels.am" scale setButtons 0

"labels.am" scale setEditButton 1

"labels.am" scale setIncrement 0.1

"labels.am" scale setValue 1

"labels.am" scale setSubMinMax 0 1

"labels.am" fire

"labels.am" setViewerMask 16383

set hideNewModules 0

%Set data directory path after the word “load”. Then copy all the scripts to a TXT file and change the file extension as “.hx”. Next drag and drop the “.hx” file onto the Amira console to run the scripts.

[ load ${SCRIPTDIR}/AD.Optimized.view.am ] setLabel "AD.Optimized.view.am"

"AD.Optimized.view.am" setIconPosition 20 10

"AD.Optimized.view.am" sharedColormap disconnect

"AD.Optimized.view.am" sharedColormap setDefaultColor 0.8 0.8 0.8

"AD.Optimized.view.am" sharedColormap setDefaultAlpha 0.500000

"AD.Optimized.view.am" sharedColormap activateLocalRange 1

"AD.Optimized.view.am" sharedColormap setLocalMinMax 0.000000 1.000000

"AD.Optimized.view.am" sharedColormap enableAlpha 1

"AD.Optimized.view.am" sharedColormap enableAlphaToggle 1

"AD.Optimized.view.am" sharedColormap setAutoAdjustRangeMode 1

"AD.Optimized.view.am" sharedColormap setColorbarMinMax 0 255

"AD.Optimized.view.am" fire

"AD.Optimized.view.am" fire

"AD.Optimized.view.am" setViewerMask 16383

"AD.Optimized.view.am" select

set hideNewModules 0

%Apply Volume Rendering module to allow for real-time rendering

create HxVolumeRenderingSettings "Volume Rendering Settings"

"Volume Rendering Settings" setViewerMask 0

"Volume Rendering Settings" setIconPosition 675 10

"Volume Rendering Settings" setVar "CustomHelp" {HxVolumeRenderingSettings}

set hideNewModules 0

"Volume Rendering Settings" data connect "AD.Optimized.view.am"

"Volume Rendering Settings" fire

"Volume Rendering Settings" rendering setValue 0

"Volume Rendering Settings" fire

"Volume Rendering Settings" interpolationAdvanced setValue 1

"Volume Rendering Settings" composition setValue 0

"Volume Rendering Settings" moveLowResolutionScale setMinMax 1 10

"Volume Rendering Settings" moveLowResolutionScale setButtons 1

"Volume Rendering Settings" moveLowResolutionScale setEditButton 1

"Volume Rendering Settings" moveLowResolutionScale setIncrement 1

"Volume Rendering Settings" moveLowResolutionScale setValue 3

"Volume Rendering Settings" moveLowResolutionScale setSubMinMax 1 10

"Volume Rendering Settings" samplingQuality setMinMax 0 2

"Volume Rendering Settings" samplingQuality setButtons 0

"Volume Rendering Settings" samplingQuality setEditButton 1

"Volume Rendering Settings" samplingQuality setIncrement 0.133333

"Volume Rendering Settings" samplingQuality setValue 1

"Volume Rendering Settings" samplingQuality setSubMinMax 0 2

"Volume Rendering Settings" opacityThreshold setMinMax 0 1

"Volume Rendering Settings" opacityThreshold setButtons 0

"Volume Rendering Settings" opacityThreshold setEditButton 1

"Volume Rendering Settings" opacityThreshold setIncrement 0.05

"Volume Rendering Settings" opacityThreshold setValue 0

"Volume Rendering Settings" opacityThreshold setSubMinMax 0 1

"Volume Rendering Settings" optimizations setValue 0 0

"Volume Rendering Settings" optimizations setToggleVisible 0 1

"Volume Rendering Settings" optimizations setValue 1 1

"Volume Rendering Settings" optimizations setToggleVisible 1 1

"Volume Rendering Settings" optimizations setValue 2 0

"Volume Rendering Settings" optimizations setToggleVisible 2 1

"Volume Rendering Settings" quality setValue 1

"Volume Rendering Settings" fire

"Volume Rendering Settings" artifactsReduction setValue 0 0

"Volume Rendering Settings" artifactsReduction setToggleVisible 0 1

"Volume Rendering Settings" artifactsReduction setValue 1 1

"Volume Rendering Settings" artifactsReduction setToggleVisible 1 1

"Volume Rendering Settings" lighting setState {item 0 1 item 1 0 }

"Volume Rendering Settings" gradient setState {item 0 1 item 2 9.99999974737875e-05 }

"Volume Rendering Settings" effects setValue 0 0

"Volume Rendering Settings" effects setToggleVisible 0 1

"Volume Rendering Settings" effects setValue 1 0

"Volume Rendering Settings" effects setToggleVisible 1 1

"Volume Rendering Settings" effects setValue 2 0

"Volume Rendering Settings" effects setToggleVisible 2 1

"Volume Rendering Settings" effects setValue 3 0

"Volume Rendering Settings" effects setToggleVisible 3 1

"Volume Rendering Settings" edgeEnhancement setMinMax 0 -3.40282346638529e+038 3.40282346638529e+038

"Volume Rendering Settings" edgeEnhancement setValue 0 9.99999974737875e-005

"Volume Rendering Settings" edge2D setState {item 1 0.100000001490116 item 3 0.100000001490116 item 5 1 }

"Volume Rendering Settings" boundaryOpacity setMinMax 0 -3.40282346638529e+038 3.40282346638529e+038

"Volume Rendering Settings" boundaryOpacity setValue 0 2.5

"Volume Rendering Settings" boundaryOpacity setMinMax 1 -3.40282346638529e+038 3.40282346638529e+038

"Volume Rendering Settings" boundaryOpacity setValue 1 2.5

"Volume Rendering Settings" material setIndex 0 4

"Volume Rendering Settings" lightingStyle setIndex 0 1

"Volume Rendering Settings" toneMapping setIndex 0 0

"Volume Rendering Settings" depthOfField setValue 0

"Volume Rendering Settings" blurFactor setMinMax 0.00999999977648258 1

"Volume Rendering Settings" blurFactor setButtons 0

"Volume Rendering Settings" blurFactor setEditButton 1

"Volume Rendering Settings" blurFactor setIncrement 0.066

"Volume Rendering Settings" blurFactor setValue 0.01

"Volume Rendering Settings" blurFactor setSubMinMax 0.00999999977648258 1

"Volume Rendering Settings" specularColor setMinMax 0 1

"Volume Rendering Settings" specularColor setButtons 0

"Volume Rendering Settings" specularColor setEditButton 1

"Volume Rendering Settings" specularColor setIncrement 0.1

"Volume Rendering Settings" specularColor setValue 0.4

"Volume Rendering Settings" specularColor setSubMinMax 0 1

"Volume Rendering Settings" shininess setMinMax 0 1

"Volume Rendering Settings" shininess setButtons 0

"Volume Rendering Settings" shininess setEditButton 1

"Volume Rendering Settings" shininess setIncrement 0.1

"Volume Rendering Settings" shininess setValue 0.4

"Volume Rendering Settings" shininess setSubMinMax 0 1

"Volume Rendering Settings" fire

"Volume Rendering Settings" setViewerMask 16383

"Volume Rendering Settings" setPickable 1

set hideNewModules 0

create HxVolumeRender2 "Volume Rendering"

"Volume Rendering" setIconPosition 675 36

"Volume Rendering" setVar "CustomHelp" {HxVolumeRender2}

"Volume Rendering" data connect "AD.Optimized.view.am"

"Volume Rendering" volumeRenderingSettings connect "Volume Rendering Settings"

"Volume Rendering" fire

"Volume Rendering" colormap connect "physics.icol"

"Volume Rendering" colormap setDefaultColor 1 1 1

"Volume Rendering" colormap setDefaultAlpha 0.500000

"Volume Rendering" colormap activateLocalRange 1

"Volume Rendering" colormap setLocalMinMax 150.000000 180.000000

"Volume Rendering" colormap enableAlpha 1

"Volume Rendering" colormap enableAlphaToggle 1

"Volume Rendering" colormap setAutoAdjustRangeMode 0

"Volume Rendering" fire

"Volume Rendering" colormapLookup setValue 2

"Volume Rendering" gamma setMinMax 0.100000001490116 8

"Volume Rendering" gamma setButtons 0

"Volume Rendering" gamma setEditButton 1

"Volume Rendering" gamma setIncrement 0.526667

"Volume Rendering" gamma setValue 3

"Volume Rendering" gamma setSubMinMax 0.100000001490116 8

"Volume Rendering" alphaScale setMinMax 0 1

"Volume Rendering" alphaScale setButtons 0

"Volume Rendering" alphaScale setEditButton 1

"Volume Rendering" alphaScale setIncrement 0.1

"Volume Rendering" alphaScale setValue 1

"Volume Rendering" alphaScale setSubMinMax 0 1

"Volume Rendering" channelSelector setState {2 }

"Volume Rendering" fire

"Volume Rendering" setViewerMask 16382

"Volume Rendering" setPickable 1

set hideNewModules 0

%Apply Invert module to calculate the reverse of an image

create negative "Invert"

"Invert" setIconPosition 160 46

"Invert" setVar "CustomHelp" {negative.html}

"Invert" interpretation setValue 0

"Invert" outputLocation setIndex 0 0

"Invert" inputImage connect "AD.Optimized.view.am"

"Invert" numberOfSignificantBits setMinMax 0 0 2147483648

"Invert" numberOfSignificantBits setValue 0 8

"Invert" applyTransformToResult 1

"Invert" fire

"Invert" setViewerMask 16383

"Invert" setPickable 1

set hideNewModules 0

[ load "${SCRIPTDIR}/../A beta-20210713-files/AD.Optimized.view.inverted" ] setLabel "AD.Optimized.view.inverted"

"AD.Optimized.view.inverted" setIconPosition 20 262

"AD.Optimized.view.inverted" master connect "Invert" "ImgOut" 0

"AD.Optimized.view.inverted" sharedColormap disconnect

"AD.Optimized.view.inverted" sharedColormap setDefaultColor 0.8 0.8 0.8

"AD.Optimized.view.inverted" sharedColormap setDefaultAlpha 0.500000

"AD.Optimized.view.inverted" sharedColormap activateLocalRange 1

"AD.Optimized.view.inverted" sharedColormap setLocalMinMax 0.000000 1.000000

"AD.Optimized.view.inverted" sharedColormap enableAlpha 1

"AD.Optimized.view.inverted" sharedColormap enableAlphaToggle 1

"AD.Optimized.view.inverted" sharedColormap setAutoAdjustRangeMode 1

"AD.Optimized.view.inverted" fire

"AD.Optimized.view.inverted" fire

"AD.Optimized.view.inverted" setViewerMask 16383

set hideNewModules 0

%Apply Interactive Thresholding module to segment the region of interest

create HxInteractiveThreshold "Interactive Thresholding"

"Interactive Thresholding" setViewerMask 0

"Interactive Thresholding" setIconPosition 160 82

"Interactive Thresholding" setVar "CustomHelp" {HxInteractiveThreshold}

set hideNewModules 0

"Interactive Thresholding" data connect "AD.Optimized.view.am"

"Interactive Thresholding" inputColorMap connect "grayScale.am"

"Interactive Thresholding" inputColorMap setDefaultColor 1 0.8 0.5

"Interactive Thresholding" inputColorMap setDefaultAlpha 0.500000

"Interactive Thresholding" inputColorMap activateLocalRange 1

"Interactive Thresholding" inputColorMap setLocalMinMax 84.000000 255.000000

"Interactive Thresholding" inputColorMap enableAlpha 1

"Interactive Thresholding" inputColorMap enableAlphaToggle 1

"Interactive Thresholding" inputColorMap setAutoAdjustRangeMode 1

"Interactive Thresholding" fire

"Interactive Thresholding" preview setValue 0 1

"Interactive Thresholding" preview setToggleVisible 0 1

"Interactive Thresholding" preview setValue 1 0

"Interactive Thresholding" preview setToggleVisible 1 1

"Interactive Thresholding" orientation setValue 0

"Interactive Thresholding" sliceNumber setMinMax 0 199

"Interactive Thresholding" sliceNumber setButtons 1

"Interactive Thresholding" sliceNumber setEditButton 1

"Interactive Thresholding" sliceNumber setIncrement 1

"Interactive Thresholding" sliceNumber setValue 100

"Interactive Thresholding" sliceNumber setSubMinMax 0 199

"Interactive Thresholding" rendering setValue 1

"Interactive Thresholding" intensityRange setMinMax 0 255

"Interactive Thresholding" intensityRange setValues 160 255

"Interactive Thresholding" intensityRange setButtons 0

"Interactive Thresholding" intensityRange setEditButton 1

"Interactive Thresholding" intensityRange setIncrement 17

"Interactive Thresholding" intensityRange setSubMinMax 0 0

"Interactive Thresholding" colorMask setColor 0 0 0 1

"Interactive Thresholding" applyTransformToResult 1

"Interactive Thresholding" fire

"Interactive Thresholding" setViewerMask 16382

"Interactive Thresholding" setPickable 1

set hideNewModules 0

[ load "${SCRIPTDIR}/../A beta-20210713-files/AD.Optimized.view.thresholded" ] setLabel "AD.Optimized.view.thresholded"

"AD.Optimized.view.thresholded" setIconPosition 20 298

"AD.Optimized.view.thresholded" master connect "Interactive Thresholding" "result" 0

"AD.Optimized.view.thresholded" sharedColormap connect "labels.am"

"AD.Optimized.view.thresholded" sharedColormap setDefaultColor 0.8 0.8 0.8

"AD.Optimized.view.thresholded" sharedColormap setDefaultAlpha 0.500000

"AD.Optimized.view.thresholded" sharedColormap activateLocalRange 1

"AD.Optimized.view.thresholded" sharedColormap setLocalMinMax 1.000000 8.000000

"AD.Optimized.view.thresholded" sharedColormap enableAlpha 1

"AD.Optimized.view.thresholded" sharedColormap enableAlphaToggle 1

"AD.Optimized.view.thresholded" sharedColormap setAutoAdjustRangeMode 1

"AD.Optimized.view.thresholded" fire

"AD.Optimized.view.thresholded" primary setIndex 0 0

"AD.Optimized.view.thresholded" fire

"AD.Optimized.view.thresholded" setViewerMask 16383

set hideNewModules 0

create HxInteractiveThreshold "Interactive Thresholding 2"

"Interactive Thresholding 2" setViewerMask 0

"Interactive Thresholding 2" setIconPosition 160 118

"Interactive Thresholding 2" setVar "CustomHelp" {HxInteractiveThreshold}

set hideNewModules 0

"Interactive Thresholding 2" data connect "AD.Optimized.view.am"

"Interactive Thresholding 2" inputColorMap connect "grayScale.am"

"Interactive Thresholding 2" inputColorMap setDefaultColor 1 0.8 0.5

"Interactive Thresholding 2" inputColorMap setDefaultAlpha 0.500000

"Interactive Thresholding 2" inputColorMap activateLocalRange 1

"Interactive Thresholding 2" inputColorMap setLocalMinMax 84.000000 255.000000

"Interactive Thresholding 2" inputColorMap enableAlpha 1

"Interactive Thresholding 2" inputColorMap enableAlphaToggle 1

"Interactive Thresholding 2" inputColorMap setAutoAdjustRangeMode 1

"Interactive Thresholding 2" fire

"Interactive Thresholding 2" preview setValue 0 1

"Interactive Thresholding 2" preview setToggleVisible 0 1

"Interactive Thresholding 2" preview setValue 1 0

"Interactive Thresholding 2" preview setToggleVisible 1 1

"Interactive Thresholding 2" orientation setValue 0

"Interactive Thresholding 2" sliceNumber setMinMax 0 199

"Interactive Thresholding 2" sliceNumber setButtons 1

"Interactive Thresholding 2" sliceNumber setEditButton 1

"Interactive Thresholding 2" sliceNumber setIncrement 1

"Interactive Thresholding 2" sliceNumber setValue 100

"Interactive Thresholding 2" sliceNumber setSubMinMax 0 199

"Interactive Thresholding 2" rendering setValue 1

"Interactive Thresholding 2" intensityRange setMinMax 0 255

"Interactive Thresholding 2" intensityRange setValues 0 135

"Interactive Thresholding 2" intensityRange setButtons 0

"Interactive Thresholding 2" intensityRange setEditButton 1

"Interactive Thresholding 2" intensityRange setIncrement 17

"Interactive Thresholding 2" intensityRange setSubMinMax 0 0

"Interactive Thresholding 2" colorMask setColor 0 0 0 1

"Interactive Thresholding 2" applyTransformToResult 1

"Interactive Thresholding 2" fire

"Interactive Thresholding 2" setViewerMask 16382

"Interactive Thresholding 2" setPickable 1

set hideNewModules 0

[ load "${SCRIPTDIR}/../A beta-20210713-files/AD.Optimized.view2.thresholded" ] setLabel "AD.Optimized.view2.thresholded"

"AD.Optimized.view2.thresholded" setIconPosition 20 370

"AD.Optimized.view2.thresholded" master connect "Interactive Thresholding 2" "result" 0

"AD.Optimized.view2.thresholded" sharedColormap connect "labels.am"

"AD.Optimized.view2.thresholded" sharedColormap setDefaultColor 0.8 0.8 0.8

"AD.Optimized.view2.thresholded" sharedColormap setDefaultAlpha 0.500000

"AD.Optimized.view2.thresholded" sharedColormap activateLocalRange 1

"AD.Optimized.view2.thresholded" sharedColormap setLocalMinMax 1.000000 8.000000

"AD.Optimized.view2.thresholded" sharedColormap enableAlpha 1

"AD.Optimized.view2.thresholded" sharedColormap enableAlphaToggle 1

"AD.Optimized.view2.thresholded" sharedColormap setAutoAdjustRangeMode 1

"AD.Optimized.view2.thresholded" fire

"AD.Optimized.view2.thresholded" primary setIndex 0 0

"AD.Optimized.view2.thresholded" fire

"AD.Optimized.view2.thresholded" setViewerMask 16383

set hideNewModules 0

% Apply OR Image module to perfrom image operation

create logical_orimage "OR Image"

"OR Image" setIconPosition 160 334

"OR Image" setVar "CustomHelp" {logical_orimage.html}

"OR Image" interpretation setValue 0

"OR Image" outputLocation setIndex 0 0

"OR Image" inputImage1 connect "AD.Optimized.view.thresholded"

"OR Image" inputImage2 connect "AD.Optimized.view2.thresholded"

"OR Image" applyTransformToResult 1

"OR Image" fire

"OR Image" setViewerMask 16383

"OR Image" setPickable 1

set hideNewModules 0

[ load "${SCRIPTDIR}/../A beta-20210713-files/AD.Optimized.view.or" ] setLabel "AD.Optimized.view.or"

"AD.Optimized.view.or" setIconPosition 20 406

"AD.Optimized.view.or" master connect "OR Image" "ImgOut" 0

"AD.Optimized.view.or" sharedColormap connect "labels.am"

"AD.Optimized.view.or" sharedColormap setDefaultColor 0.8 0.8 0.8

"AD.Optimized.view.or" sharedColormap setDefaultAlpha 0.500000

"AD.Optimized.view.or" sharedColormap activateLocalRange 1

"AD.Optimized.view.or" sharedColormap setLocalMinMax 1.000000 8.000000

"AD.Optimized.view.or" sharedColormap enableAlpha 1

"AD.Optimized.view.or" sharedColormap enableAlphaToggle 1

"AD.Optimized.view.or" sharedColormap setAutoAdjustRangeMode 1

"AD.Optimized.view.or" fire

"AD.Optimized.view.or" primary setIndex 0 0

"AD.Optimized.view.or" fire

"AD.Optimized.view.or" setViewerMask 16383

set hideNewModules 0

%Apply Multiply By Value module to multiply an image by a constant C

create multiplyvalue "Multiply By Value"

"Multiply By Value" setIconPosition 160 442

"Multiply By Value" setVar "CustomHelp" {multiplyvalue.html}

"Multiply By Value" interpretation setValue 0

"Multiply By Value" outputLocation setIndex 0 0

"Multiply By Value" inputImage1 connect "AD.Optimized.view.or"

"Multiply By Value" value setMinMax 0 -3.40282346638529e+038 3.40282346638529e+038

"Multiply By Value" value setValue 0 135

"Multiply By Value" applyTransformToResult 1

"Multiply By Value" fire

"Multiply By Value" setViewerMask 16383

"Multiply By Value" setPickable 1

set hideNewModules 0

[ load "${SCRIPTDIR}/../A beta-20210713-files/AD.Optimized.view.mult" ] setLabel "AD.Optimized.view.mult"

"AD.Optimized.view.mult" setIconPosition 20 514

"AD.Optimized.view.mult" master connect "Multiply By Value" "ImgOut" 0

"AD.Optimized.view.mult" sharedColormap disconnect

"AD.Optimized.view.mult" sharedColormap setDefaultColor 0.8 0.8 0.8

"AD.Optimized.view.mult" sharedColormap setDefaultAlpha 0.500000

"AD.Optimized.view.mult" sharedColormap activateLocalRange 1

"AD.Optimized.view.mult" sharedColormap setLocalMinMax 0.000000 1.000000

"AD.Optimized.view.mult" sharedColormap enableAlpha 1

"AD.Optimized.view.mult" sharedColormap enableAlphaToggle 1

"AD.Optimized.view.mult" sharedColormap setAutoAdjustRangeMode 1

"AD.Optimized.view.mult" fire

"AD.Optimized.view.mult" fire

"AD.Optimized.view.mult" setViewerMask 16383

set hideNewModules 0

%Apply Invert module to calculate the reverse of an image

create negative "Invert 2"

"Invert 2" setIconPosition 160 478

"Invert 2" setVar "CustomHelp" {negative.html}

"Invert 2" interpretation setValue 0

"Invert 2" outputLocation setIndex 0 0

"Invert 2" inputImage connect "AD.Optimized.view.or"

"Invert 2" numberOfSignificantBits setMinMax 0 0 2147483648

"Invert 2" numberOfSignificantBits setValue 0 8

"Invert 2" applyTransformToResult 1

"Invert 2" fire

"Invert 2" setViewerMask 16383

"Invert 2" setPickable 1

set hideNewModules 0

[ load "${SCRIPTDIR}/../A beta-20210713-files/AD.Optimized.view2.inverted" ] setLabel "AD.Optimized.view2.inverted"

"AD.Optimized.view2.inverted" setIconPosition 20 586

"AD.Optimized.view2.inverted" master connect "Invert 2" "ImgOut" 0

"AD.Optimized.view2.inverted" sharedColormap connect "labels.am"

"AD.Optimized.view2.inverted" sharedColormap setDefaultColor 0.8 0.8 0.8

"AD.Optimized.view2.inverted" sharedColormap setDefaultAlpha 0.500000

"AD.Optimized.view2.inverted" sharedColormap activateLocalRange 1

"AD.Optimized.view2.inverted" sharedColormap setLocalMinMax 1.000000 8.000000

"AD.Optimized.view2.inverted" sharedColormap enableAlpha 1

"AD.Optimized.view2.inverted" sharedColormap enableAlphaToggle 1

"AD.Optimized.view2.inverted" sharedColormap setAutoAdjustRangeMode 1

"AD.Optimized.view2.inverted" fire

"AD.Optimized.view2.inverted" primary setIndex 0 0

"AD.Optimized.view2.inverted" fire

"AD.Optimized.view2.inverted" setViewerMask 16383

set hideNewModules 0

%Apply Multiply By Value module to multiply an image by a constant C

create multiplyimage "Multiply By Image"

"Multiply By Image" setIconPosition 160 154

"Multiply By Image" setVar "CustomHelp" {multiplyimage.html}

"Multiply By Image" interpretation setValue 0

"Multiply By Image" outputLocation setIndex 0 0

"Multiply By Image" inputImage1 connect "AD.Optimized.view.am"

"Multiply By Image" inputImage2 connect "AD.Optimized.view2.inverted"

"Multiply By Image" applyTransformToResult 1

"Multiply By Image" fire

"Multiply By Image" setViewerMask 16383

"Multiply By Image" setPickable 1

set hideNewModules 0

[ load "${SCRIPTDIR}/../A beta-20210713-files/AD.Optimized.view2.mult" ] setLabel "AD.Optimized.view2.mult"

"AD.Optimized.view2.mult" setIconPosition 20 622

"AD.Optimized.view2.mult" master connect "Multiply By Image" "ImgOut" 0

"AD.Optimized.view2.mult" sharedColormap disconnect

"AD.Optimized.view2.mult" sharedColormap setDefaultColor 0.8 0.8 0.8

"AD.Optimized.view2.mult" sharedColormap setDefaultAlpha 0.500000

"AD.Optimized.view2.mult" sharedColormap activateLocalRange 1

"AD.Optimized.view2.mult" sharedColormap setLocalMinMax 0.000000 1.000000

"AD.Optimized.view2.mult" sharedColormap enableAlpha 1

"AD.Optimized.view2.mult" sharedColormap enableAlphaToggle 1

"AD.Optimized.view2.mult" sharedColormap setAutoAdjustRangeMode 1

"AD.Optimized.view2.mult" fire

"AD.Optimized.view2.mult" fire

"AD.Optimized.view2.mult" setViewerMask 16383

set hideNewModules 0

%Apply Add Image module to add two images

create addimage "Add Image"

"Add Image" setIconPosition 160 550

"Add Image" setVar "CustomHelp" {addimage.html}

"Add Image" interpretation setValue 0

"Add Image" outputLocation setIndex 0 0

"Add Image" inputImage1 connect "AD.Optimized.view2.mult"

"Add Image" inputImage2 connect "AD.Optimized.view.mult"

"Add Image" applyTransformToResult 1

"Add Image" fire

"Add Image" setViewerMask 16383

"Add Image" setPickable 1

set hideNewModules 0

[ load "${SCRIPTDIR}/../A beta-20210713-files/AD.Optimized.view2.add" ] setLabel "AD.Optimized.view2.add"

"AD.Optimized.view2.add" setIconPosition 20 658

"AD.Optimized.view2.add" master connect "Add Image" "ImgOut" 0

"AD.Optimized.view2.add" sharedColormap disconnect

"AD.Optimized.view2.add" sharedColormap setDefaultColor 0.8 0.8 0.8

"AD.Optimized.view2.add" sharedColormap setDefaultAlpha 0.500000

"AD.Optimized.view2.add" sharedColormap activateLocalRange 1

"AD.Optimized.view2.add" sharedColormap setLocalMinMax 0.000000 1.000000

"AD.Optimized.view2.add" sharedColormap enableAlpha 1

"AD.Optimized.view2.add" sharedColormap enableAlphaToggle 1

"AD.Optimized.view2.add" sharedColormap setAutoAdjustRangeMode 1

"AD.Optimized.view2.add" fire

"AD.Optimized.view2.add" fire

"AD.Optimized.view2.add" setViewerMask 16383

set hideNewModules 0

%Apply Median Filter module to use lowpass filters to reduce the contrast and soften the edges of objects in an image

create medianfilter "Median Filter"

"Median Filter" setIconPosition 160 694

"Median Filter" setVar "CustomHelp" {medianfilter3d.html}

"Median Filter" interpretation setValue 0

"Median Filter" outputLocation setIndex 0 0

"Median Filter" neighborhood setValue 2

"Median Filter" inputImage connect "AD.Optimized.view2.add"

"Median Filter" type setIndex 0 0

"Median Filter" iterations setMinMax 0 0 2147483648

"Median Filter" iterations setValue 0 30

"Median Filter" applyTransformToResult 1

"Median Filter" fire

"Median Filter" setViewerMask 16383

"Median Filter" setPickable 1

set hideNewModules 0

[ load "${SCRIPTDIR}/../A beta-20210713-files/AD.Optimized.view2.filtered" ] setLabel "AD.Optimized.view2.filtered"

"AD.Optimized.view2.filtered" setIconPosition 20 766

"AD.Optimized.view2.filtered" master connect "Median Filter" "ImgOut" 0

"AD.Optimized.view2.filtered" sharedColormap disconnect

"AD.Optimized.view2.filtered" sharedColormap setDefaultColor 0.8 0.8 0.8

"AD.Optimized.view2.filtered" sharedColormap setDefaultAlpha 0.500000

"AD.Optimized.view2.filtered" sharedColormap activateLocalRange 1

"AD.Optimized.view2.filtered" sharedColormap setLocalMinMax 0.000000 1.000000

"AD.Optimized.view2.filtered" sharedColormap enableAlpha 1

"AD.Optimized.view2.filtered" sharedColormap enableAlphaToggle 1

"AD.Optimized.view2.filtered" sharedColormap setAutoAdjustRangeMode 1

"AD.Optimized.view2.filtered" fire

"AD.Optimized.view2.filtered" fire

"AD.Optimized.view2.filtered" setViewerMask 16383

set hideNewModules 0

%Apply Ortho Slice module for visualizing scalar data fields defined on uniform Cartesian grids

create HxOrthoSlice "Ortho Slice"

"Ortho Slice" setIconPosition 842 828

"Ortho Slice" setVar "CustomHelp" {HxOrthoSlice}

"Ortho Slice" data connect "AD.Optimized.view2.filtered"

"Ortho Slice" fire

"Ortho Slice" sliceOrientation setValue 0

"Ortho Slice" fire

"Ortho Slice" origin setBoundingBox -3.40282e+038 3.40282e+038 -3.40282e+038 3.40282e+038 -3.40282e+038 3.40282e+038

"Ortho Slice" origin setImmediateMode 0

"Ortho Slice" origin setOrtho 0

"Ortho Slice" origin showDragger 0

"Ortho Slice" origin showPoints 0

"Ortho Slice" origin setPointScale 1

"Ortho Slice" origin showOptionButton 0

"Ortho Slice" origin setNumPoints 1 1 1

"Ortho Slice" origin setCoord 0 1936.55 2561.13 100

"Ortho Slice" normal setBoundingBox -3.40282e+038 3.40282e+038 -3.40282e+038 3.40282e+038 -3.40282e+038 3.40282e+038

"Ortho Slice" normal setImmediateMode 0

"Ortho Slice" normal setOrtho 0

"Ortho Slice" normal showDragger 0

"Ortho Slice" normal showPoints 0

"Ortho Slice" normal setPointScale 1

"Ortho Slice" normal showOptionButton 0

"Ortho Slice" normal setNumPoints 1 1 1

"Ortho Slice" normal setCoord 0 0 0 1

"Ortho Slice" options setValue 0 0

"Ortho Slice" options setToggleVisible 0 1

"Ortho Slice" options setValue 1 0

"Ortho Slice" options setToggleVisible 1 1

"Ortho Slice" options setValue 2 0

"Ortho Slice" options setToggleVisible 2 1

"Ortho Slice" mappingType setIndex 0 0

"Ortho Slice" contrastLimit setMinMax 0 -16777216 16777216

"Ortho Slice" contrastLimit setValue 0 7

"Ortho Slice" colormap connect "grayScale.am"

"Ortho Slice" colormap setDefaultColor 1 0.8 0.5

"Ortho Slice" colormap setDefaultAlpha 1.000000

"Ortho Slice" colormap activateLocalRange 1

"Ortho Slice" colormap setLocalMinMax 135.000000 159.000000

"Ortho Slice" colormap enableAlpha 1

"Ortho Slice" colormap enableAlphaToggle 1

"Ortho Slice" colormap setAutoAdjustRangeMode 1

"Ortho Slice" sliceNumber setMinMax 0 199

"Ortho Slice" sliceNumber setButtons 1

"Ortho Slice" sliceNumber setEditButton 1

"Ortho Slice" sliceNumber setIncrement 1

"Ortho Slice" sliceNumber setValue 100

"Ortho Slice" sliceNumber setSubMinMax 0 199

"Ortho Slice" transparency setValue 0

"Ortho Slice" alpha setMinMax 0 -16777216 16777216

"Ortho Slice" alpha setValue 0 0

"Ortho Slice" alpha setMinMax 1 -16777216 16777216

"Ortho Slice" alpha setValue 1 255

"Ortho Slice" frameSettings setState {item 0 1 item 2 1 color 3 1 0.5 0 }

"Ortho Slice" embossingOnOff setValue 0

"Ortho Slice" depth setMinMax -60 60

"Ortho Slice" depth setButtons 0

"Ortho Slice" depth setEditButton 1

"Ortho Slice" depth setIncrement 8

"Ortho Slice" depth setValue 2

"Ortho Slice" depth setSubMinMax -60 60

"Ortho Slice" fire

"Ortho Slice" fire

"Ortho Slice" setViewerMask 16382

"Ortho Slice" setShadowStyle 0

"Ortho Slice" setPickable 1

set hideNewModules 0

%Apply Volume Rendering module to allow for real-time rendering

create HxVolumeRenderingSettings "Volume Rendering Settings 2"

"Volume Rendering Settings 2" setViewerMask 0

"Volume Rendering Settings 2" setIconPosition 651 766

"Volume Rendering Settings 2" setVar "CustomHelp" {HxVolumeRenderingSettings}

set hideNewModules 0

"Volume Rendering Settings 2" data connect "AD.Optimized.view2.filtered"

"Volume Rendering Settings 2" fire

"Volume Rendering Settings 2" rendering setValue 0

"Volume Rendering Settings 2" fire

"Volume Rendering Settings 2" interpolationAdvanced setValue 1

"Volume Rendering Settings 2" composition setValue 0

"Volume Rendering Settings 2" moveLowResolutionScale setMinMax 1 10

"Volume Rendering Settings 2" moveLowResolutionScale setButtons 1

"Volume Rendering Settings 2" moveLowResolutionScale setEditButton 1

"Volume Rendering Settings 2" moveLowResolutionScale setIncrement 1

"Volume Rendering Settings 2" moveLowResolutionScale setValue 3

"Volume Rendering Settings 2" moveLowResolutionScale setSubMinMax 1 10

"Volume Rendering Settings 2" samplingQuality setMinMax 0 2

"Volume Rendering Settings 2" samplingQuality setButtons 0

"Volume Rendering Settings 2" samplingQuality setEditButton 1

"Volume Rendering Settings 2" samplingQuality setIncrement 0.133333

"Volume Rendering Settings 2" samplingQuality setValue 1

"Volume Rendering Settings 2" samplingQuality setSubMinMax 0 2

"Volume Rendering Settings 2" opacityThreshold setMinMax 0 1

"Volume Rendering Settings 2" opacityThreshold setButtons 0

"Volume Rendering Settings 2" opacityThreshold setEditButton 1

"Volume Rendering Settings 2" opacityThreshold setIncrement 0.05

"Volume Rendering Settings 2" opacityThreshold setValue 0

"Volume Rendering Settings 2" opacityThreshold setSubMinMax 0 1

"Volume Rendering Settings 2" optimizations setValue 0 0

"Volume Rendering Settings 2" optimizations setToggleVisible 0 1

"Volume Rendering Settings 2" optimizations setValue 1 1

"Volume Rendering Settings 2" optimizations setToggleVisible 1 1

"Volume Rendering Settings 2" optimizations setValue 2 0

"Volume Rendering Settings 2" optimizations setToggleVisible 2 1

"Volume Rendering Settings 2" quality setValue 1

"Volume Rendering Settings 2" fire

"Volume Rendering Settings 2" artifactsReduction setValue 0 0

"Volume Rendering Settings 2" artifactsReduction setToggleVisible 0 1

"Volume Rendering Settings 2" artifactsReduction setValue 1 1

"Volume Rendering Settings 2" artifactsReduction setToggleVisible 1 1

"Volume Rendering Settings 2" lighting setState {item 0 1 item 1 0 }

"Volume Rendering Settings 2" gradient setState {item 0 1 item 2 9.99999974737875e-05 }

"Volume Rendering Settings 2" effects setValue 0 0

"Volume Rendering Settings 2" effects setToggleVisible 0 1

"Volume Rendering Settings 2" effects setValue 1 0

"Volume Rendering Settings 2" effects setToggleVisible 1 1

"Volume Rendering Settings 2" effects setValue 2 0

"Volume Rendering Settings 2" effects setToggleVisible 2 1

"Volume Rendering Settings 2" effects setValue 3 0

"Volume Rendering Settings 2" effects setToggleVisible 3 1

"Volume Rendering Settings 2" edgeEnhancement setMinMax 0 -3.40282346638529e+038 3.40282346638529e+038

"Volume Rendering Settings 2" edgeEnhancement setValue 0 9.99999974737875e-005

"Volume Rendering Settings 2" edge2D setState {item 1 0.100000001490116 item 3 0.100000001490116 item 5 1 }

"Volume Rendering Settings 2" boundaryOpacity setMinMax 0 -3.40282346638529e+038 3.40282346638529e+038

"Volume Rendering Settings 2" boundaryOpacity setValue 0 2.5

"Volume Rendering Settings 2" boundaryOpacity setMinMax 1 -3.40282346638529e+038 3.40282346638529e+038

"Volume Rendering Settings 2" boundaryOpacity setValue 1 2.5

"Volume Rendering Settings 2" material setIndex 0 4

"Volume Rendering Settings 2" lightingStyle setIndex 0 1

"Volume Rendering Settings 2" toneMapping setIndex 0 0

"Volume Rendering Settings 2" depthOfField setValue 0

"Volume Rendering Settings 2" blurFactor setMinMax 0.00999999977648258 1

"Volume Rendering Settings 2" blurFactor setButtons 0

"Volume Rendering Settings 2" blurFactor setEditButton 1

"Volume Rendering Settings 2" blurFactor setIncrement 0.066

"Volume Rendering Settings 2" blurFactor setValue 0.01

"Volume Rendering Settings 2" blurFactor setSubMinMax 0.00999999977648258 1

"Volume Rendering Settings 2" specularColor setMinMax 0 1

"Volume Rendering Settings 2" specularColor setButtons 0

"Volume Rendering Settings 2" specularColor setEditButton 1

"Volume Rendering Settings 2" specularColor setIncrement 0.1

"Volume Rendering Settings 2" specularColor setValue 0.4

"Volume Rendering Settings 2" specularColor setSubMinMax 0 1

"Volume Rendering Settings 2" shininess setMinMax 0 1

"Volume Rendering Settings 2" shininess setButtons 0

"Volume Rendering Settings 2" shininess setEditButton 1

"Volume Rendering Settings 2" shininess setIncrement 0.1

"Volume Rendering Settings 2" shininess setValue 0.4

"Volume Rendering Settings 2" shininess setSubMinMax 0 1

"Volume Rendering Settings 2" fire

"Volume Rendering Settings 2" setViewerMask 16383

"Volume Rendering Settings 2" setPickable 1

set hideNewModules 0

%Apply Volume Rendering module to allow for real-time rendering

create HxVolumeRender2 "Volume Rendering 2"

"Volume Rendering 2" setIconPosition 651 792

"Volume Rendering 2" setVar "CustomHelp" {HxVolumeRender2}

"Volume Rendering 2" data connect "AD.Optimized.view2.filtered"

"Volume Rendering 2" volumeRenderingSettings connect "Volume Rendering Settings 2"

"Volume Rendering 2" fire

"Volume Rendering 2" colormap connect "volrenGreen.col"

"Volume Rendering 2" colormap setDefaultColor 1 1 1

"Volume Rendering 2" colormap setDefaultAlpha 0.500000

"Volume Rendering 2" colormap activateLocalRange 1

"Volume Rendering 2" colormap setLocalMinMax 145.000000 156.000000

"Volume Rendering 2" colormap enableAlpha 1

"Volume Rendering 2" colormap enableAlphaToggle 1

"Volume Rendering 2" colormap setAutoAdjustRangeMode 1

"Volume Rendering 2" fire

"Volume Rendering 2" colormapLookup setValue 2

"Volume Rendering 2" gamma setMinMax 0.100000001490116 8

"Volume Rendering 2" gamma setButtons 0

"Volume Rendering 2" gamma setEditButton 1

"Volume Rendering 2" gamma setIncrement 0.526667

"Volume Rendering 2" gamma setValue 3

"Volume Rendering 2" gamma setSubMinMax 0.100000001490116 8

"Volume Rendering 2" alphaScale setMinMax 0 1

"Volume Rendering 2" alphaScale setButtons 0

"Volume Rendering 2" alphaScale setEditButton 1

"Volume Rendering 2" alphaScale setIncrement 0.1

"Volume Rendering 2" alphaScale setValue 1

"Volume Rendering 2" alphaScale setSubMinMax 0 1

"Volume Rendering 2" channelSelector setState {2 }

"Volume Rendering 2" fire

"Volume Rendering 2" setViewerMask 16382

"Volume Rendering 2" setPickable 1

set hideNewModules 0

%Apply Bilateral Filter module to conduct edge-preserving smoothing

create bilateralfilter "Bilateral Filter"

"Bilateral Filter" setIconPosition 160 730

"Bilateral Filter" setVar "CustomHelp" {bilateralfilter3d.html}

"Bilateral Filter" interpretation setValue 0

"Bilateral Filter" outputLocation setIndex 0 0

"Bilateral Filter" inputImage connect "AD.Optimized.view2.add"

"Bilateral Filter" kernelSizeX setMinMax 0 3 100

"Bilateral Filter" kernelSizeX setValue 0 21

"Bilateral Filter" kernelSizeY setMinMax 0 3 100

"Bilateral Filter" kernelSizeY setValue 0 21

"Bilateral Filter" kernelSizeZ setMinMax 0 3 100

"Bilateral Filter" kernelSizeZ setValue 0 7

"Bilateral Filter" similarity setMinMax 0 0 3.40282346638529e+038

"Bilateral Filter" similarity setValue 0 20

"Bilateral Filter" filterMode setIndex 0 0

"Bilateral Filter" applyTransformToResult 1

"Bilateral Filter" fire

"Bilateral Filter" setViewerMask 16383

"Bilateral Filter" setPickable 1

set hideNewModules 0

[ load ${SCRIPTDIR}/AD.Optimized.view3.filtered.am ] setLabel "AD.Optimized.view3.filtered.am"

"AD.Optimized.view3.filtered.am" setIconPosition 20 802

"AD.Optimized.view3.filtered.am" master connect "Bilateral Filter" "ImgOut" 0

"AD.Optimized.view3.filtered.am" sharedColormap disconnect

"AD.Optimized.view3.filtered.am" sharedColormap setDefaultColor 0.8 0.8 0.8

"AD.Optimized.view3.filtered.am" sharedColormap setDefaultAlpha 0.500000

"AD.Optimized.view3.filtered.am" sharedColormap activateLocalRange 1

"AD.Optimized.view3.filtered.am" sharedColormap setLocalMinMax 0.000000 1.000000

"AD.Optimized.view3.filtered.am" sharedColormap enableAlpha 1

"AD.Optimized.view3.filtered.am" sharedColormap enableAlphaToggle 1

"AD.Optimized.view3.filtered.am" sharedColormap setAutoAdjustRangeMode 1

"AD.Optimized.view3.filtered.am" fire

"AD.Optimized.view3.filtered.am" fire

"AD.Optimized.view3.filtered.am" setViewerMask 16383

set hideNewModules 0

%Apply Interactive Thresholding module to segment the region of interest

create HxInteractiveThreshold "Interactive Thresholding 3"

"Interactive Thresholding 3" setViewerMask 0

"Interactive Thresholding 3" setIconPosition 160 838

"Interactive Thresholding 3" setVar "CustomHelp" {HxInteractiveThreshold}

set hideNewModules 0

"Interactive Thresholding 3" data connect "AD.Optimized.view3.filtered.am"

"Interactive Thresholding 3" inputColorMap connect "grayScale.am"

"Interactive Thresholding 3" inputColorMap setDefaultColor 1 0.8 0.5

"Interactive Thresholding 3" inputColorMap setDefaultAlpha 0.500000

"Interactive Thresholding 3" inputColorMap activateLocalRange 1

"Interactive Thresholding 3" inputColorMap setLocalMinMax 135.000000 155.000000

"Interactive Thresholding 3" inputColorMap enableAlpha 1

"Interactive Thresholding 3" inputColorMap enableAlphaToggle 1

"Interactive Thresholding 3" inputColorMap setAutoAdjustRangeMode 1

"Interactive Thresholding 3" fire

"Interactive Thresholding 3" preview setValue 0 1

"Interactive Thresholding 3" preview setToggleVisible 0 1

"Interactive Thresholding 3" preview setValue 1 0

"Interactive Thresholding 3" preview setToggleVisible 1 1

"Interactive Thresholding 3" orientation setValue 0

"Interactive Thresholding 3" sliceNumber setMinMax 0 199

"Interactive Thresholding 3" sliceNumber setButtons 1

"Interactive Thresholding 3" sliceNumber setEditButton 1

"Interactive Thresholding 3" sliceNumber setIncrement 1

"Interactive Thresholding 3" sliceNumber setValue 100

"Interactive Thresholding 3" sliceNumber setSubMinMax 0 199

"Interactive Thresholding 3" rendering setValue 1

"Interactive Thresholding 3" intensityRange setMinMax -2147483648 2147483648

"Interactive Thresholding 3" intensityRange setValues 146 156

"Interactive Thresholding 3" intensityRange setButtons 0

"Interactive Thresholding 3" intensityRange setEditButton 1

"Interactive Thresholding 3" intensityRange setIncrement 2.86331e+008

"Interactive Thresholding 3" intensityRange setSubMinMax 0 0

"Interactive Thresholding 3" colorMask setColor 0 0 0 1

"Interactive Thresholding 3" applyTransformToResult 1

"Interactive Thresholding 3" fire

"Interactive Thresholding 3" setViewerMask 16382

"Interactive Thresholding 3" setPickable 1

set hideNewModules 0

[ load ${SCRIPTDIR}/AD.Optimized.view3.filtered.thresholded.am ] setLabel "AD.Optimized.view3.filtered.thresholded.am"

"AD.Optimized.view3.filtered.thresholded.am" setIconPosition 20 874

"AD.Optimized.view3.filtered.thresholded.am" master connect "Interactive Thresholding 3" "result" 0

"AD.Optimized.view3.filtered.thresholded.am" sharedColormap connect "labels.am"

"AD.Optimized.view3.filtered.thresholded.am" sharedColormap setDefaultColor 0.8 0.8 0.8

"AD.Optimized.view3.filtered.thresholded.am" sharedColormap setDefaultAlpha 0.500000

"AD.Optimized.view3.filtered.thresholded.am" sharedColormap activateLocalRange 1

"AD.Optimized.view3.filtered.thresholded.am" sharedColormap setLocalMinMax 1.000000 8.000000

"AD.Optimized.view3.filtered.thresholded.am" sharedColormap enableAlpha 1

"AD.Optimized.view3.filtered.thresholded.am" sharedColormap enableAlphaToggle 1

"AD.Optimized.view3.filtered.thresholded.am" sharedColormap setAutoAdjustRangeMode 1

"AD.Optimized.view3.filtered.thresholded.am" fire

"AD.Optimized.view3.filtered.thresholded.am" primary setIndex 0 0

"AD.Optimized.view3.filtered.thresholded.am" fire

"AD.Optimized.view3.filtered.thresholded.am" setViewerMask 16383

set hideNewModules 0

%Apply Ortho Slice module for visualizing scalar data fields defined on uniform Cartesian grids

create HxOrthoSlice "Ortho Slice 2"

"Ortho Slice 2" setIconPosition 819 658

"Ortho Slice 2" setVar "CustomHelp" {HxOrthoSlice}

"Ortho Slice 2" data connect "AD.Optimized.view2.add"

"Ortho Slice 2" fire

"Ortho Slice 2" sliceOrientation setValue 0

"Ortho Slice 2" fire

"Ortho Slice 2" origin setBoundingBox -3.40282e+038 3.40282e+038 -3.40282e+038 3.40282e+038 -3.40282e+038 3.40282e+038

"Ortho Slice 2" origin setImmediateMode 0

"Ortho Slice 2" origin setOrtho 0

"Ortho Slice 2" origin showDragger 0

"Ortho Slice 2" origin showPoints 0

"Ortho Slice 2" origin setPointScale 1

"Ortho Slice 2" origin showOptionButton 0

"Ortho Slice 2" origin setNumPoints 1 1 1

"Ortho Slice 2" origin setCoord 0 1936.55 2561.13 105

"Ortho Slice 2" normal setBoundingBox -3.40282e+038 3.40282e+038 -3.40282e+038 3.40282e+038 -3.40282e+038 3.40282e+038

"Ortho Slice 2" normal setImmediateMode 0

"Ortho Slice 2" normal setOrtho 0

"Ortho Slice 2" normal showDragger 0

"Ortho Slice 2" normal showPoints 0

"Ortho Slice 2" normal setPointScale 1

"Ortho Slice 2" normal showOptionButton 0

"Ortho Slice 2" normal setNumPoints 1 1 1

"Ortho Slice 2" normal setCoord 0 0 0 1

"Ortho Slice 2" options setValue 0 0

"Ortho Slice 2" options setToggleVisible 0 1

"Ortho Slice 2" options setValue 1 0

"Ortho Slice 2" options setToggleVisible 1 1

"Ortho Slice 2" options setValue 2 0

"Ortho Slice 2" options setToggleVisible 2 1

"Ortho Slice 2" mappingType setIndex 0 0

"Ortho Slice 2" contrastLimit setMinMax 0 -16777216 16777216

"Ortho Slice 2" contrastLimit setValue 0 7

"Ortho Slice 2" colormap connect "grayScale.am"

"Ortho Slice 2" colormap setDefaultColor 1 0.8 0.5

"Ortho Slice 2" colormap setDefaultAlpha 1.000000

"Ortho Slice 2" colormap activateLocalRange 1

"Ortho Slice 2" colormap setLocalMinMax 135.000000 159.000000

"Ortho Slice 2" colormap enableAlpha 1

"Ortho Slice 2" colormap enableAlphaToggle 1

"Ortho Slice 2" colormap setAutoAdjustRangeMode 1

"Ortho Slice 2" sliceNumber setMinMax 0 199

"Ortho Slice 2" sliceNumber setButtons 1

"Ortho Slice 2" sliceNumber setEditButton 1

"Ortho Slice 2" sliceNumber setIncrement 1

"Ortho Slice 2" sliceNumber setValue 105

"Ortho Slice 2" sliceNumber setSubMinMax 0 199

"Ortho Slice 2" transparency setValue 0

"Ortho Slice 2" alpha setMinMax 0 -16777216 16777216

"Ortho Slice 2" alpha setValue 0 0

"Ortho Slice 2" alpha setMinMax 1 -16777216 16777216

"Ortho Slice 2" alpha setValue 1 255

"Ortho Slice 2" frameSettings setState {item 0 1 item 2 1 color 3 1 0.5 0 }

"Ortho Slice 2" embossingOnOff setValue 0

"Ortho Slice 2" depth setMinMax -60 60

"Ortho Slice 2" depth setButtons 0

"Ortho Slice 2" depth setEditButton 1

"Ortho Slice 2" depth setIncrement 8

"Ortho Slice 2" depth setValue 2

"Ortho Slice 2" depth setSubMinMax -60 60

"Ortho Slice 2" fire

"Ortho Slice 2" fire

"Ortho Slice 2" setViewerMask 16382

"Ortho Slice 2" setShadowStyle 0

"Ortho Slice 2" setPickable 1

set hideNewModules 0

%Apply Closing module to fill the holes inside the particles, eliminates the small details by smoothing the boundary from the outside and connects close particles

create closing "Closing"

"Closing" setIconPosition 160 910

"Closing" setVar "CustomHelp" {closing}

"Closing" Type setState {type Cube}

"Closing" interpretation setValue 0

"Closing" outputLocation setIndex 0 0

"Closing" neighborhood setValue 2

"Closing" inputImage connect "AD.Optimized.view3.filtered.thresholded.am"

"Closing" size setMinMax 0 0 2147483648

"Closing" size setValue 0 5

"Closing" applyTransformToResult 1

"Closing" fire

"Closing" setViewerMask 16383

"Closing" setPickable 1

set hideNewModules 0

[ load ${SCRIPTDIR}/AD.Optimized.view3.filtered.closing.am ] setLabel "AD.Optimized.view3.filtered.closing.am"

"AD.Optimized.view3.filtered.closing.am" setIconPosition 20 946

"AD.Optimized.view3.filtered.closing.am" master connect "Closing" "ImgOut" 0

"AD.Optimized.view3.filtered.closing.am" sharedColormap connect "labels.am"

"AD.Optimized.view3.filtered.closing.am" sharedColormap setDefaultColor 0.8 0.8 0.8

"AD.Optimized.view3.filtered.closing.am" sharedColormap setDefaultAlpha 0.500000

"AD.Optimized.view3.filtered.closing.am" sharedColormap activateLocalRange 1

"AD.Optimized.view3.filtered.closing.am" sharedColormap setLocalMinMax 1.000000 8.000000

"AD.Optimized.view3.filtered.closing.am" sharedColormap enableAlpha 1

"AD.Optimized.view3.filtered.closing.am" sharedColormap enableAlphaToggle 1

"AD.Optimized.view3.filtered.closing.am" sharedColormap setAutoAdjustRangeMode 1

"AD.Optimized.view3.filtered.closing.am" fire

"AD.Optimized.view3.filtered.closing.am" primary setIndex 0 0

"AD.Optimized.view3.filtered.closing.am" fire

"AD.Optimized.view3.filtered.closing.am" setViewerMask 16383

set hideNewModules 0

%Apply Dilation module to fill the small holes inside particles and gulfs at the object boundaries, enlarges the size of the particles and may connect neighboring particles.

create dilate "Dilation"

"Dilation" setIconPosition 160 982

"Dilation" setVar "CustomHelp" {dilate}

"Dilation" Type setState {type Cube}

"Dilation" interpretation setValue 0

"Dilation" outputLocation setIndex 0 0

"Dilation" neighborhood setValue 2

"Dilation" inputImage connect "AD.Optimized.view3.filtered.closing.am"

"Dilation" size setMinMax 0 0 2147483648

"Dilation" size setValue 0 3

"Dilation" applyTransformToResult 1

"Dilation" fire

"Dilation" setViewerMask 16383

"Dilation" setPickable 1

set hideNewModules 0

[ load ${SCRIPTDIR}/AD.Optimized.view3.filtered.dilated.am ] setLabel "AD.Optimized.view3.filtered.dilated.am"

"AD.Optimized.view3.filtered.dilated.am" setIconPosition 20 1018

"AD.Optimized.view3.filtered.dilated.am" master connect "Dilation" "ImgOut" 0

"AD.Optimized.view3.filtered.dilated.am" sharedColormap connect "labels.am"

"AD.Optimized.view3.filtered.dilated.am" sharedColormap setDefaultColor 0.8 0.8 0.8

"AD.Optimized.view3.filtered.dilated.am" sharedColormap setDefaultAlpha 0.500000

"AD.Optimized.view3.filtered.dilated.am" sharedColormap activateLocalRange 1

"AD.Optimized.view3.filtered.dilated.am" sharedColormap setLocalMinMax 1.000000 8.000000

"AD.Optimized.view3.filtered.dilated.am" sharedColormap enableAlpha 1

"AD.Optimized.view3.filtered.dilated.am" sharedColormap enableAlphaToggle 1

"AD.Optimized.view3.filtered.dilated.am" sharedColormap setAutoAdjustRangeMode 1

"AD.Optimized.view3.filtered.dilated.am" fire

"AD.Optimized.view3.filtered.dilated.am" primary setIndex 0 0

"AD.Optimized.view3.filtered.dilated.am" fire

"AD.Optimized.view3.filtered.dilated.am" setViewerMask 16383

set hideNewModules 0

%Apply Multiply By Image module to multiplie an image by another one

create multiplyimage "Multiply By Image 2"

"Multiply By Image 2" setIconPosition 160 190

"Multiply By Image 2" setVar "CustomHelp" {multiplyimage.html}

"Multiply By Image 2" interpretation setValue 0

"Multiply By Image 2" outputLocation setIndex 0 0

"Multiply By Image 2" inputImage1 connect "AD.Optimized.view.am"

"Multiply By Image 2" inputImage2 connect "AD.Optimized.view3.filtered.dilated.am"

"Multiply By Image 2" applyTransformToResult 1

"Multiply By Image 2" fire

"Multiply By Image 2" setViewerMask 16383

"Multiply By Image 2" setPickable 1

set hideNewModules 0

[ load ${SCRIPTDIR}/AD.Optimized.view3.mult.am ] setLabel "AD.Optimized.view3.mult.am"

"AD.Optimized.view3.mult.am" setIconPosition 20 1054

"AD.Optimized.view3.mult.am" master connect "Multiply By Image 2" "ImgOut" 0

"AD.Optimized.view3.mult.am" sharedColormap disconnect

"AD.Optimized.view3.mult.am" sharedColormap setDefaultColor 0.8 0.8 0.8

"AD.Optimized.view3.mult.am" sharedColormap setDefaultAlpha 0.500000

"AD.Optimized.view3.mult.am" sharedColormap activateLocalRange 1

"AD.Optimized.view3.mult.am" sharedColormap setLocalMinMax 0.000000 1.000000

"AD.Optimized.view3.mult.am" sharedColormap enableAlpha 1

"AD.Optimized.view3.mult.am" sharedColormap enableAlphaToggle 1

"AD.Optimized.view3.mult.am" sharedColormap setAutoAdjustRangeMode 1

"AD.Optimized.view3.mult.am" fire

"AD.Optimized.view3.mult.am" fire

"AD.Optimized.view3.mult.am" setViewerMask 16383

set hideNewModules 0

%Apply Interactive Thresholding module to segment the region of interest

create HxInteractiveThreshold "Interactive Thresholding 4"

"Interactive Thresholding 4" setViewerMask 0

"Interactive Thresholding 4" setIconPosition 160 1090

"Interactive Thresholding 4" setVar "CustomHelp" {HxInteractiveThreshold}

set hideNewModules 0

"Interactive Thresholding 4" data connect "AD.Optimized.view3.mult.am"

"Interactive Thresholding 4" inputColorMap connect "grayScale.am"

"Interactive Thresholding 4" inputColorMap setDefaultColor 1 0.8 0.5

"Interactive Thresholding 4" inputColorMap setDefaultAlpha 0.500000

"Interactive Thresholding 4" inputColorMap activateLocalRange 1

"Interactive Thresholding 4" inputColorMap setLocalMinMax 0.000000 255.000000

"Interactive Thresholding 4" inputColorMap enableAlpha 1

"Interactive Thresholding 4" inputColorMap enableAlphaToggle 1

"Interactive Thresholding 4" inputColorMap setAutoAdjustRangeMode 1

"Interactive Thresholding 4" fire

"Interactive Thresholding 4" preview setValue 0 1

"Interactive Thresholding 4" preview setToggleVisible 0 1

"Interactive Thresholding 4" preview setValue 1 0

"Interactive Thresholding 4" preview setToggleVisible 1 1

"Interactive Thresholding 4" orientation setValue 0

"Interactive Thresholding 4" sliceNumber setMinMax 0 199

"Interactive Thresholding 4" sliceNumber setButtons 1

"Interactive Thresholding 4" sliceNumber setEditButton 1

"Interactive Thresholding 4" sliceNumber setIncrement 1

"Interactive Thresholding 4" sliceNumber setValue 99

"Interactive Thresholding 4" sliceNumber setSubMinMax 0 199

"Interactive Thresholding 4" rendering setValue 1

"Interactive Thresholding 4" intensityRange setMinMax 0 65535

"Interactive Thresholding 4" intensityRange setValues 150 170

"Interactive Thresholding 4" intensityRange setButtons 0

"Interactive Thresholding 4" intensityRange setEditButton 1

"Interactive Thresholding 4" intensityRange setIncrement 4369

"Interactive Thresholding 4" intensityRange setSubMinMax 0 0

"Interactive Thresholding 4" colorMask setColor 0 0 0 1

"Interactive Thresholding 4" applyTransformToResult 1

"Interactive Thresholding 4" fire

"Interactive Thresholding 4" setViewerMask 16382

"Interactive Thresholding 4" setPickable 1

set hideNewModules 0

[ load ${SCRIPTDIR}/AD.Optimized.view3.thresholded.am ] setLabel "AD.Optimized.view3.thresholded.am"

"AD.Optimized.view3.thresholded.am" setIconPosition 20 1162

"AD.Optimized.view3.thresholded.am" master connect "Interactive Thresholding 4" "result" 0

"AD.Optimized.view3.thresholded.am" sharedColormap connect "labels.am"

"AD.Optimized.view3.thresholded.am" sharedColormap setDefaultColor 0.8 0.8 0.8

"AD.Optimized.view3.thresholded.am" sharedColormap setDefaultAlpha 0.500000

"AD.Optimized.view3.thresholded.am" sharedColormap activateLocalRange 1

"AD.Optimized.view3.thresholded.am" sharedColormap setLocalMinMax 1.000000 8.000000

"AD.Optimized.view3.thresholded.am" sharedColormap enableAlpha 1

"AD.Optimized.view3.thresholded.am" sharedColormap enableAlphaToggle 1

"AD.Optimized.view3.thresholded.am" sharedColormap setAutoAdjustRangeMode 1

"AD.Optimized.view3.thresholded.am" fire

"AD.Optimized.view3.thresholded.am" primary setIndex 0 0

"AD.Optimized.view3.thresholded.am" fire

"AD.Optimized.view3.thresholded.am" setViewerMask 16383

set hideNewModules 0

%Apply Label Analysis module to compute a group of measures on each cell or connected component of the input image, and generates a spreadsheet with the result values

create HxAnalyzeLabels "Label Analysis"

"Label Analysis" setIconPosition 160 1198

"Label Analysis" setVar "CustomHelp" {HxAnalyzeLabels}

"Label Analysis" data connect "AD.Optimized.view3.thresholded.am"

"Label Analysis" fire

"Label Analysis" interpretation setValue 0

"Label Analysis" sequenceMode setValue 0

labelMeasure setAttributes feret2d 0 18 36 54 72 90 108 126 144 162

labelMeasure setAttributes feret3d 31

labelMeasure setAttributes cooccurrence 0 0

labelMeasure setAttributes histogram 1 0 255 1

labelMeasure setAttributes quantile 0.1 0.1 0.1 0.1 0.1 0.1

labelMeasure setAttributes breadth3d 10

labelMeasure setAttributes neighborCount 0 5 0

"Label Analysis" measures setState {"Standard Shape Analysis" Volume3d BaryCenterX BaryCenterY BaryCenterZ Anisotropy Elongation Flatness EigenVal1 EigenVal2 EigenVal3 EigenVec1X EigenVec1Y EigenVec1Z EigenVec2X EigenVec2Y EigenVec2Z EigenVec3X EigenVec3Y EigenVec3Z ExtentMin1 ExtentMin2 ExtentMin3 ExtentMax1 ExtentMax2 ExtentMax3 BinMom2x BinMom2y BinMom2z BinMomxy BinMomxz BinMomyz VoxelFaceArea BorderVoxelCount GreyMass}

"Label Analysis" showAnalysis setValue 0 1

"Label Analysis" showAnalysis setToggleVisible 0 1

"Label Analysis" applyTransformToResult 1

"Label Analysis" fire

"Label Analysis" setViewerMask 16383

"Label Analysis" setPickable 1

set hideNewModules 0

[ load ${SCRIPTDIR}/AD.Optimized.view3.Label-Analysis.am ] setLabel "AD.Optimized.view3.Label-Analysis.am"

"AD.Optimized.view3.Label-Analysis.am" setIconPosition 20 1306

"AD.Optimized.view3.Label-Analysis.am" master connect "Label Analysis" "AnlOut" 0

"AD.Optimized.view3.Label-Analysis.am" fire

"AD.Optimized.view3.Label-Analysis.am" fire

"AD.Optimized.view3.Label-Analysis.am" setViewerMask 16383

set hideNewModules 0

[ load ${SCRIPTDIR}/AD.Optimized.view3.label.am ] setLabel "AD.Optimized.view3.label.am"

"AD.Optimized.view3.label.am" setIconPosition 20 1234

"AD.Optimized.view3.label.am" master connect "Label Analysis" "ImgOutLab" 1

"AD.Optimized.view3.label.am" sharedColormap connect "labels.am"

"AD.Optimized.view3.label.am" sharedColormap setDefaultColor 0.8 0.8 0.8

"AD.Optimized.view3.label.am" sharedColormap setDefaultAlpha 0.500000

"AD.Optimized.view3.label.am" sharedColormap activateLocalRange 1

"AD.Optimized.view3.label.am" sharedColormap setLocalMinMax 1.000000 8.000000

"AD.Optimized.view3.label.am" sharedColormap enableAlpha 1

"AD.Optimized.view3.label.am" sharedColormap enableAlphaToggle 1

"AD.Optimized.view3.label.am" sharedColormap setAutoAdjustRangeMode 1

"AD.Optimized.view3.label.am" fire

"AD.Optimized.view3.label.am" primary setIndex 0 0

"AD.Optimized.view3.label.am" fire

"AD.Optimized.view3.label.am" setViewerMask 16383

set hideNewModules 0

%Apply Analysis Filter module to remove from the input label analysis labels whose measure does not fulfill a filter formula

create HxFilterAnalysis "Analysis Filter"

"Analysis Filter" setIconPosition 160 1270

"Analysis Filter" setVar "CustomHelp" {HxFilterAnalysis}

"Analysis Filter" data connect "AD.Optimized.view3.Label-Analysis.am"

"Analysis Filter" image connect "AD.Optimized.view3.label.am"

"Analysis Filter" fire

"Analysis Filter" filter setState { Volume3d > 125}

"Analysis Filter" showAnalysis setValue 0 1

"Analysis Filter" showAnalysis setToggleVisible 0 1

"Analysis Filter" applyTransformToResult 1

"Analysis Filter" fire

"Analysis Filter" setViewerMask 16383

"Analysis Filter" setPickable 1

set hideNewModules 0

[ load ${SCRIPTDIR}/AD.Optimized.view3.Analysis-Filter.am ] setLabel "AD.Optimized.view3.Analysis-Filter.am"

"AD.Optimized.view3.Analysis-Filter.am" setIconPosition 20 1342

"AD.Optimized.view3.Analysis-Filter.am" master connect "Analysis Filter" "AnlOut" 0

"AD.Optimized.view3.Analysis-Filter.am" fire

"AD.Optimized.view3.Analysis-Filter.am" fire

"AD.Optimized.view3.Analysis-Filter.am" setViewerMask 16383

set hideNewModules 0

[ load ${SCRIPTDIR}/AD.Optimized.view3.label-filtering.am ] setLabel "AD.Optimized.view3.label-filtering.am"

"AD.Optimized.view3.label-filtering.am" setIconPosition 20 1378

"AD.Optimized.view3.label-filtering.am" master connect "Analysis Filter" "result1" 1

"AD.Optimized.view3.label-filtering.am" sharedColormap connect "labels.am"

"AD.Optimized.view3.label-filtering.am" sharedColormap setDefaultColor 0.8 0.8 0.8

"AD.Optimized.view3.label-filtering.am" sharedColormap setDefaultAlpha 0.500000

"AD.Optimized.view3.label-filtering.am" sharedColormap activateLocalRange 1

"AD.Optimized.view3.label-filtering.am" sharedColormap setLocalMinMax 1.000000 8.000000

"AD.Optimized.view3.label-filtering.am" sharedColormap enableAlpha 1

"AD.Optimized.view3.label-filtering.am" sharedColormap enableAlphaToggle 1

"AD.Optimized.view3.label-filtering.am" sharedColormap setAutoAdjustRangeMode 1

"AD.Optimized.view3.label-filtering.am" fire

"AD.Optimized.view3.label-filtering.am" primary setIndex 0 0

"AD.Optimized.view3.label-filtering.am" fire

"AD.Optimized.view3.label-filtering.am" setViewerMask 16383

set hideNewModules 0

%Apply Generate Surface module to compute a triangular approximation of the interfaces between different regions or materials in a Label Field with either uniform or stacked coordinates

create HxGMC "Generate Surface"

"Generate Surface" setIconPosition 160 1414

"Generate Surface" setVar "CustomHelp" {HxGMC}

"Generate Surface" data connect "AD.Optimized.view3.label-filtering.am"

"Generate Surface" fire

"Generate Surface" smoothing setIndex 0 3

"Generate Surface" smoothingExtent setMinMax 1 9

"Generate Surface" smoothingExtent setButtons 0

"Generate Surface" smoothingExtent setEditButton 1

"Generate Surface" smoothingExtent setIncrement 0.533333

"Generate Surface" smoothingExtent setValue 5

"Generate Surface" smoothingExtent setSubMinMax 1 9

"Generate Surface" options setState {item 0 0 item 1 0 }

"Generate Surface" borderOnOff setValue 1

"Generate Surface" algorithmMode setIndex 0 0

"Generate Surface" borderSettings setValue 0 1

"Generate Surface" borderSettings setToggleVisible 0 1

"Generate Surface" borderSettings setValue 1 0

"Generate Surface" borderSettings setToggleVisible 1 1

"Generate Surface" borderSettings setValue 2 0

"Generate Surface" borderSettings setToggleVisible 2 1

"Generate Surface" borderSettings setValue 3 0

"Generate Surface" borderSettings setToggleVisible 3 0

"Generate Surface" materialList setIndex 0 0

"Generate Surface" smoothMaterial setIndex 0 0

"Generate Surface" applyTransformToResult 1

"Generate Surface" fire

"Generate Surface" setViewerMask 16383

"Generate Surface" setPickable 1

set hideNewModules 0

[ load ${SCRIPTDIR}/AD.Optimized.view3.surf.am ] setLabel "AD.Optimized.view3.surf.am"

"AD.Optimized.view3.surf.am" setIconPosition 20 1450

"AD.Optimized.view3.surf.am" master connect "Generate Surface" "result" 0

"AD.Optimized.view3.surf.am" fire

"AD.Optimized.view3.surf.am" LevelOfDetail setMinMax -1 -1

"AD.Optimized.view3.surf.am" LevelOfDetail setButtons 1

"AD.Optimized.view3.surf.am" LevelOfDetail setEditButton 1

"AD.Optimized.view3.surf.am" LevelOfDetail setIncrement 1

"AD.Optimized.view3.surf.am" LevelOfDetail setValue -1

"AD.Optimized.view3.surf.am" LevelOfDetail setSubMinMax -1 -1

"AD.Optimized.view3.surf.am" fire

"AD.Optimized.view3.surf.am" setViewerMask 16383

set hideNewModules 0

%Apply Surface View module to visualize triangular surfaces, i.e., data objects of type Surface

create HxDisplaySurface "Surface View"

"Surface View" setIconPosition 829 1450

"Surface View" setVar "CustomHelp" {HxDisplaySurface}

"Surface View" data connect "AD.Optimized.view3.surf.am"

"Surface View" colormap disconnect

"Surface View" colormap setDefaultColor 1 0.1 0.1

"Surface View" colormap setDefaultAlpha 0.500000

"Surface View" colormap activateLocalRange 1

"Surface View" colormap setLocalMinMax 0.000000 1.000000

"Surface View" colormap enableAlpha 1

"Surface View" colormap enableAlphaToggle 1

"Surface View" colormap setAutoAdjustRangeMode 1

"Surface View" fire

"Surface View" drawStyle setValue 1

"Surface View" fire

"Surface View" drawStyle setSpecularLighting 1

"Surface View" drawStyle setTexture 1

"Surface View" drawStyle setAlphaMode 1

"Surface View" drawStyle setCullingMode 0

"Surface View" drawStyle setNormalBinding 0

"Surface View" drawStyle setSortingMode 1

"Surface View" drawStyle setLineWidth 0.000000

"Surface View" drawStyle setOutlineColor 0 0 0.2

"Surface View" textureWrap setIndex 0 1

"Surface View" selectionMode setIndex 0 0

"Surface View" patch setMinMax 0 1803

"Surface View" patch setButtons 1

"Surface View" patch setEditButton 1

"Surface View" patch setIncrement 1

"Surface View" patch setValue 0

"Surface View" patch setSubMinMax 0 1803

"Surface View" boundaryId setIndex 0 -1

"Surface View" materials setIndex 0 1

"Surface View" materials setIndex 1 0

"Surface View" colorMode setIndex 0 0

"Surface View" baseTrans setMinMax 0 1

"Surface View" baseTrans setButtons 0

"Surface View" baseTrans setEditButton 1

"Surface View" baseTrans setIncrement 0.1

"Surface View" baseTrans setValue 0.8

"Surface View" baseTrans setSubMinMax 0 1

"Surface View" VRMode setIndex 0 0

"Surface View" colorFieldMappingType setValue 0

"Surface View" setLighting 1

"Surface View" fire

"Surface View" hideBox 1

"Surface View" selectTriangles zab HIJMOMNAMBAJAADAAMACEADHOIPOFLHGIDJEOMFAAIIEDLPAKPFGABAAAAAAAAAAAAAAAAAAAAAAAAAAAAAAAAAAAAAAAAAAAAAAAAAAAAAAAAAAAAAAAAAAAAAAAAAAAAAAAAAAAAAAAAAAAAAAAAAAAAAAAAAAAAAAAAAAAAAAAAAAAAAAAAAAAAAAAAAAAAAAAAAAAAAAAAAAAAAAAAAAAAAAAAAAAAAAAAAAAAAAAAAAAAAAAAAAAAAAAAAAAAAAAAAAAAAAAAAAAAAAAAAAAAAAAAAAAAAAAAAAAAAAAAAAAAAAAAAAAAAAAAAAAAAAAAAAAAAAAAAAAAAAAAAAAAAAAAAAAAAAAAAAAAAAAAAAAAAAAAAAAAAAAAAAAAAAAAAAAAAAAAAAAAAAAAAAAAAAAAAAAAAAAAAAAAAAAAAAAAAAAAAAAAAAAAAAAAAAAAAAAAAAAAAAAAAAAAAAAAAAAAAAAAAAAAAAAAAAAAAAAAAAAAAAAAAAAAAAAAAAAAAAAAAAAAAAAAAAAAAAAAAAAAAAAAAAAAAAAAAAAAAAAAAAAAAAAAAAAAAAAAAAAAAAAAAAAAAAAAAAAAAAAAAAAAAAAAAAAAAAAAAAAAAAAAAAAAAAAAAAAAAAAAAAAAAAAAAAAAAAAAAAAAAAAAAAAAAAAAAAAAAAAAAAAAAAAAAAAAAAAAAAAAAAAAAAAAAAAAAAAAAAAAAAAAAAAAAAAAAAAAAAAAAAAAAAAAAAAAAAAAAAAAAAAAAAAAAAAAAAAAAAAAAAAAAAAAAAAAAAAAAAAAAAAAAAAAAAAAAAAAAAAAAAAAAAAAAAAAAAAAAAAAAAAAAAAAAAAAAAAAAAAAAAAAAAAAAAAAAAAAAAAAAAAAAAAAAAAAAAAAAAAAAAAAAAAAAAAAAAAAAAAAAAAAAAAAAAAAAAAAAAAAAAAAAAAAAAAAAAAAAAAAAAAAAAAAAAAAAAAAAAAAAAAAAAAAAAAAAAAAAAAAAAAAAAAAAAAAAAAAAAAAAAAAAAAAAAAAAAAAAAAAAAAAAAAAAAAAAAAAAAAAAAAAAAAAAAAAAAAAAAAAAAAAAAAAAAAAAAAAAAAAAAAAAAAAAAAAAAAAAAAAAAAAAAAAAAAAAAAAAAAAAAAAAAAAAAAAAAAAAAAAAAAAAAAAAAAAAAAAAAAAAAAAAAAAAAAAAAAAAAAAAAAAAAAAAAAAAAAAAAAAAAAAAAAAAAAAAAAAAAAAAAAAAAAAAAAAAAAAAAAAAAAAAAAAAAAAAAAAAAAAAAAAAAAAAAAAAAAAAAAAAAAAAAAAAAAAAAAAAAAAAAAAAAAAAAAAAAAAAAAAAAAAAAAAAAAAAAAAAAAAAAAAAAAAAAAAAAAAAAAAAAAAAAAAAAAAAAAAAAAAAAAAAAAAAAAAAAAAAAAAAAAAAAAAAAAAAAAAAAAAAAAAAAAAAAAAAAAAAAAAAAAAAAAAAAAAAAAAAAAAAAAAAAAAAAAAAAAAAAAAAAAAAAAAAAAAAAAAAAAAAAAAAAAAAAAAAAAAAAAAAAAAAAAAAAAAAAAAAAAAAAAAAAAAAAAAAAAAAAAAAAAAAAAAAAAAAAAAAAAAAAAAAAAAAAAAAAAAAAAAAAAAAAAAAAAAAAAAAAAAAAAAAAAAAAAAAAAAAAAAAAAAAAAAAAAAAAAAAAAAAAAAAAAAAAAAAAAAAAAAAAAAAAAAAAAAAAAAAAAAAAAAAAAAAAAAAAAAAAAAAAAAAAAAAAAAAAAAAAAAAAAAAAAAAAAAAAAAAAAAAAAAAAAAAAAAAAAAAAAAAAAAAAAAAAAAAAAAAAAAAAAAAAAAAAAAAAAAAAAAAAAAAAAAAAAAAAAAAAAAAAAAAAAAAAAAAAAAAAAAAAAAAAAAAAAAAAAAAAAAAAAAAAAAAAAAAAAAAAAAAAAAAAAAAAAAAAAAAAAAAAAAAAAAAAAAAAAAAAAAAAAAAAAAAAAAAAAAAAAAAAAAAAAAAAAAAAAAAAAAAAAAAAAAAAAAAAAAAAAAAAAAAAAAAAAAAAAAAAAAAAAAAAAAAAAAAAAAAAAAAAAAAAAAAAAAAAAAAAAAAAAAAAAAAAAAAAAAAAAAAAAAAAAAAAAAAAAAAAAAAAAAAAAAAAAAAAAAAAAAAAAAAAAAAAAAAAAAAAAAAAAAAAAAAAAAAAAAAAAAAAAAAAAAAAAAAAAAAAAAAAAAAAAAAAAAAAAAAAAAAAAAAAAAAAAAAAAAAAAAAAAAAAAAAAAAAAAAAAAAAAAAAAAAAAAAAAAAAAAAAAAAAAAAAAAAAAAAAAAAAAAAAAAAAAAAAAAAAAAAAAAAAAAAAAAAAAAAAAAAAAAAAAAAAAAAAAAAAAAAAAAAAAAAAAAAAAAAAAAAAAAAAAAAAAAAAAAAAAAAAAAAAAAAAAAAAAAAAAAAAAAAAAAAAAAAAAAAAAAAAAAAAAAAAAAAAAAAAAAAAAAAAAAAAAAAAAAAAAAAAAAAAAAAAAAAAAAAAAAAAAAAAAAAAAAAAAAAAAAAAAAAAAAAAAAAAAAAAAAAAAAAAAAAAAAAAAAAAAAAAAAAAAAAAAAAAAAAAAAAAAAAAAAAAAAAAAAAAAAAAAAAAAAAAAAAAAAAAAAAAAAAAAAAAAAAAAAAAAAAOMJGOEGEFABHJIPOAAAAAAAAAAAAAAAAAAAAAAAAAAAAAAAAAAAAAAAAAAAAAAAAAAAAAAAAAAAAAAAAAAAAAAAAAAAAAAAAAAAAAAAAAAAAAAAAAAAAAAAAAAAAAAAAAAAAAAAAAAAAAAAAAAAAAAAAAAAAAAAAAAAAAAAAAAAAAAAAAAAAAAAAAAAAAAAAAAAAAAIAJNNCPJKAGODCLNAFAAAAAAAAAAAAAAAAAAAAAAAAAAAAAAAAAAAAAAAAAAAAAAAAAAAAAAAAAAAAAAAAAAAAAAAAAAAAAAAAAAAAAAAAAAAAAAAAAAAAAAAAAAAAAAAAAAAAAAAAAAAAAAAAAAAAAAAAAAAAAAAAAAAAAAAAAAAAAAAAAAAAAAAAAAAAAAAAAAAAAAAAAAAAAAAAAAAAAAAAAAAAAAAAAAAAAAAAAAAAAAAAAAAAAAAAAAAAAAAAAAAAAAAAAAAAAAAAAAAAAAAAAAAAAAAAAAAAAAAAAAAAAAAAAAAAAAAAAAAAAAAAAAAAAAAAAAAAAAAAAAAAAAAAAAAAAAAAAAAAAAAAAAAAAAAAAAAAAAAAAAAAAAAAAAAAAAAAAAAAAAAAAAAAAAAAAAAAAAAAAAAAAAAAAAAAAAAAAAAAAAAAAAAAAAAAAAAAAAAAAAAAAAAAAAAAAAAAAAAAAAAAAAAAAAAAAAAAAAAAAAAAAAAAAAAAAAAAAAAAAAAAAAAAAAAAAAAAAAAAAAAAAAAAAAAAAAAAAAAAAAAAAAAAAAAAAAAAAAAAAAAAAAAAAAAAAAAAAAAAAAAAAAAAAAAAAAAAAAAAAAAAAAAAAAAAAAAAAAAAAAAAAAAAAAAAAAAAAAAAAAAAAAAAAAAAAAAAAAAAAAAAAAAAAAAAAAAAAAAAAAAAAAAAAAAAAAAAAAAAAAAAAAAAAAAAAAAAAAAAAAAAAAAAAAAAAAAAAAAAAAAAAAAAAAAAAAAAAAAAAAAAAAAAAAAAAAAAAAAAAAAAAAAAAAAAAAAAAAAAAAAAAAAAAAAAAAAAAAAAAAAAAAAAAAAAAAAAAAAAAAAAAAAAAAAAAAAAAAAAAAAAAAAAAAAAAAAAAAAAAAAAAAAAAAAAAAAAAAAAAAAAAAAAAAAAAAAAAAAAAAAAAAAAAAAAAAAAAAAAAAAAAAAAAAAAAAAAAAAAAAAAAAAAAAAAAAAAAAAAAAAAAAAAAAAAAAAAAAAAAAAAAAAAAAAAAAAAAAAAAAAAAAAAAAAAAAAAAAAAAAAAAAAAAAAAAAAAAAAAAAAAAAAAAAAAAAAAAAAAAAAAAAAAAAAAAAAAAAAAAAAAAAAAAAAAAAAAAAAAAAAAAAAAAAAAAAAAAAAAAAAAAAAAAAAAAAAAAAAAAAAAAAAAAAAAAAAAAAAAAAAAAAAAAAAAAAAAAAAAAAAAAAAAAAAAAAAAAAAAAAAAAAAAAAAAAAAAAAAAAAAAAAAAAAAAAAAAAAAAAAAAAAAAAAAAAAAAAAAAAAAAAAAAAAAAAAAAAAAAAAAAAAAAAAAAAAAAAAAAAAAAAAAAAAAAAAAAAAAAAAAAAAAAAAAAAAAAAAAAAAAAAAAAAAAAAAAAAAAAAAAAAAAAAAAAAAAAAAAAAAAAAAAAAAAAAAAAAAAAAAAAAAAAAAAAAAAAAAAAAAAAAAAAAAAAAAAAAAAAAAAAAAAAAAAAAAAAAAAAAAAAAAAAAAAAAAAAAAAAAAAAAAAAAAAAAAAAAAAAAAAAAAAAAAAAAAAAAAAAAAAAAAAAAAAAAAAAAAAAAAAAAAAAAAAAAAAAAAAAAAAAAAAAAAAAAAAAAAAAAAAAAAAAAAAAAAAAAAAAAAAAAAAAAAAAAAAAAAAAAAAAAAAAAAAAAAAAAAAAAAAAAAAAAAAAAAAAAAAAAAAAAAAAAAAAAAAAAAAAAAAAAAAAAAAAAAAAAAAAAAAAAAAAAAAAAAAAAAAAAAAAAAAAAAAAAAAAAAAAAAAAAAAAAAAAAAAAAAAAAAAAAAAAAAAAAAAAAAAAAAAAAAAAAAAAAAAAAAAAAAAAAAAAAAAAAAAAAAAAAAAAAAAAAAAAAAAAAAAAAAAAAAAAAAAAAAAAAAAAAAAAAAAAAAAAAAAAAAAAAAAAAAAAAAAAAAAAAAAAAAAAAAAAAAAAAAAAAAAAAAAAAAAAAAAAAAAAAAAAAAAAAAAAAAAAAAAAAAAAAAAAAAAAAAAAAAAAAAAAAAAAAAAAAAAAAAAAAAAAAAAAAAAAAAAAAAAAAAAAAAAAAAAAAAAAAAAAAAAAAAAAAAAAAAAAAAAAAAAAAAAAAAAAAAAAAAAAAAAAAAAAAAAAAAAAAAAAAAAAAAAAAAAAAAAAAAAAAAAAAAAAAAAAAAAAAAAAAAAAAAAAAAAAAAAAAAAAAAAAAAAAAAAAAAAAAAAAAAAAAAAAAAAAAAAAAAAAAAAAAAAAAAAAAAAAAAAAAAAAAAAAAAAAAAAAAAAAAAAAAAAAAAAAAAAAAAAAAAAAAAAAAAAAAAAAAAAAAAAAAAAAAAAAAAAAAAAAAAAAAAAAAAAAAAAAAAAAAAAAAAAAAAAAAAAAAAAAAAAAAAAAAAAAAAAAAAAAAAAAAAAAAAAAAAAAAAAAAAAAAAAAAAAAAAAAAAAAAAAAAAAAAAAAAAAAAAAAAAAAAAAAAAAAAAAAAAAAAAAAAAAAAAAAAAAAAAAAAAAAAAAAAAAAAAAAAAAAAAAAAAAAAAAAAAAAAAAAAAAAAAAAAAAAAAAAAAAAAAAAAAAAAAAAAAAAAAAAAAAAAAAAAAAAAAAAAAAAAAAAAAAAAAAAAAAAAAAAAAAAAAAAAAAAAAAAAAAAAAAAAAAAAAAAAAAAAAAAAAAAAAAAAAAAAAAAAAAAAAAAAAAAAAAAAAAAAAAAAAAAAAAAAAAAAAAAAAAAAAAAAAAAAAAAAAAAAAAAAAAAAAAAAAAAAAAAAAAAAAAAAAAAAAAAAAAAAAAAAAAAAAAAAAAAAAAAAAAAAAAAAAAAAAAAAAAAAAAAAAAAAAAAAAAAAAAAAAAAAAAAAAAAAAAAAAAAAAAAAAAAAAAAAAAAAAAAAAAAAAAAAAAAAAAAAAAAAAAAAAAAAAAAAAAAAAAAAAAAAAAAAAAAAAAAAAAAAAAAAAAAAAAAAAAAAAAAAAAAAAAAAAAAAAAAAAAAAAAAAAAAAAAAAAAAAAAAAAAAAAAAAAAAAAAAAAAAAAAAAAAAAAAAAAAAAAAAAAAAAAAAAAAAAAAAAAAAAAAAAAAAAAAAAAAAAAAAAAAAAAAAAAAAAAAAAAAAAAAAAAAAAAAAAAAAAAAAAAAAAAAAAAAAAAAAAAAAAAAAAAAAAAAAAAAAAAAAAAAAAAAAAAAAAAAAAAAAAAAAAAAAAAAAAAAAAAAAAAAAAAAAAAAAAAAAAAAAAAAAAAAAAAAAAAAAAAAAAAAAAAAAAAAAAAAAAAAAAAAAAAAAAAAAAAAAAAAAAAAAAAAAAAAAAAAAAAAAAAAAAAAAAAAAAAAAAAAAAAAAAAAAAAAAAAAAAAAAAAAAAAAAAAAAAAAAAAAAAAAAAAAAAAAAAAAAAAAAAAAAAAAAAAAAAAAAAAAAAAAAAAAAAAAAAAAAAAAAAAAAAAAAAAAAAAAAAAAAAAAAAAAAAAAAAAAAAAAAAAAAAAAAAAAAAAAAAAAAAAAAAAAAAAAAAAAAAAAAAAAAAAAAAAAAAAAAAAAAAAAAAAAAAAAAAAAAAAAAAAAAAAAAAAAAAAAAAAAAAAAAAAAAAAAAAAAAAAAAAAAAAAAAAAAAAAAAAAAAAAAAAAAAAAAAAAAAAAAAAAAAAAAAAAAAAAAAAAAAAAAAAAAAAAAAAAAAAAAAAAAAAAAAAAAAAAAAAAAAAAAAAAAAAAAAAAAAAAAAAAAAAAAAAAAAAAAAAAAAAAAAAAAAAAAAAAAAAAAAAAAAAAAAAAAAAAAAAAAAAAAAAAAAAAAAAAAAAAAAAAAAAAAAAAAAAAAAAAAAAAAAAAAAAAAAAAAAAAAAAAAAAAAAAAAAAAAAAAAAAAAAAAAAAAAAAAAAAAAAAAAAAAAAAAAAAAAAAAAAAAAAAAAAAAAAAAAAAAAAAAAAAAAAAAAAAAAAAAAAAAAAAAAAAAAAAAAAAAAAAAAAAAAAAAAAAAAAAAAAAAAAAAAAAAAAAAAAAAAAAAAAAAAAAAAAAAAAAAAAAAAAAAAAAAAAAAAAAAAAAAAAAAAAAAAAAAAAAAAAAAAAAAAAAAAAAAAAAAAAAAAAAAAAAAAAAAAAAAAAAAAAAAAAAAAAAAAAAAAAAAAAAAAAAAAAAAAAAAAAAAAAAAAAAAAAAAAAAAAAAAAAAAAAAAAAAAAAAAAAAAAAAAAAAAAAAAAAAAAAAAAAAAAAAAAAAAAAAAAAAAAAAAAAAAAAAAAAAAAAAAAAAAAAAAAAAAAAAAAAAAAAAAAAAAAAAAAAAAAAAAAAAAAAAAAAAAAAAAAAAAAAAAAAAAAAAAAAAAAAAAAAAAAAAAAAAAAAAAAAAAAAAAAAAAAAAAAAAAAAAAAAAAAAAAAAAAAAAAAAAAAAAAAAAAAAAAAAAAAAAAAAAAAAAAAAAAAAAAAAAAAAAAAAAAAAAAAAAAAAAAAAAAAAAAAAAAAAAAAAAAAAAAAAAAAAAAAAAAAAAAAAAAAAAAAAAAAAAAAAAAAAAAAAAAAAAAAAAAAAAAAAAAAAAAAAAAAAAAAAAAAAAAAAAAAAAAAAAAAAAAAAAAAAAAAAAAAAAAAAAAAAAAAAAAAAAAAAAAAAAAAAAAAAAAAAAAAAAAAAAAAAAAAAAAAAAAAAAAAAAAAAAAAAAAAAAAAAAAAAAAAAAAAAAAAAAAAAAAAAAAAAAAAAAAAAAAAAAAAAAAAAAAAAAAAAAAAAAAAAAAAAAAAAAAAAAAAAAAAAAAAAAAAAAAAAAAAAAAAAAAAAAAAAAAAAAAAAAAAAAAAAAAAAAAAAAAAAAAAAAAAAAAAAAAAAAAAAAAAAAAAAAAAAAAAAAAAAAAAAAAAAAAAAAAAAAAAAAAAAAAAAAAAAAAAAAAAAAAAAAAAAAAAAAAAAAAAAAAAAAAAAAAAAAAAAAAAAAAAAAAAAAAAAAAAAAAAAAAAAAAAAAAAAAAAAAAAAAAAAAAAAAAAAAAAAAAAAAAAAAAAAAAAAAAAAAAAAAAAAAAAAAAAAAAAAAAAAAAAAAAAAAAAAAAAAAAAAAAAAAAAAAAAAAAAAAAAAAAAAAAAAAAAAAAAAAAAAAAAAAAAAAAAAAAAAAAAAAAAAAAAAAAAAAAAAAAAAAAAAAAAAAAAAAAAAAAAAAAAAAAAAAAAAAAAAAAAAAAAAAAAAAAAAAAAAAAAAAAAAAAAAAAAAAAAAAAAAAAAAAAAAAAAAAAAAAAAAAAAAAAAAAAAAAAAAAAAAAAAAAAAAAAAAAAAAAAAAAAAAAAAAAAAAAAAAAAAAAAAAAAAAAAAAAAAAAAAAAAAAAAAAAAAAAAAAAAAAAAAAAAAAAAAAAAAAAAAAAAAAAAAAAAAAAAAAAAAAAAAAAAAAAAAAAAAAAAAAAAAAAAAAAAAAAAAAAAAAAAAAAAAAAAAAAAAAAAAAAAAAAAAAAAAAAAAAAAAAAAAAAAAAAAAAAAAAAAAAFOPLHAEAACAAAAIAAAGIFKPPFPNFIEAGCIAIAAAAAAAAAAAAAAAAAAAAAAAAAAAAAAAAAAAAAAAAAAAAAAAAAAAAAAAAAAAAAAAAAAAAAAAAAAAAAAAAAAAAAAAAAAAAAAAAAAAAAAAAAAAAAAAAAAAAAAAAAAAAAAAAAAAAAAAAAAAAAAAAAAAAAAAAAAAAAAAAAAAAAAAAAAAAAAAAAAAAAAAAAAAAAAAAAAAAAAAAAAAAAAAAAAAAAAAAAAAAAAAAAAAAAAAAAAAAAAAAAAAAAAAAAAAAAAAAAAAAAAAAAAAAAAAAAAAAAAAAAAAAAAAAAAAAAAAAAAAAAAAAAAAAAAAAAAAAAAAAAAAAAAAAAAAAAAAAAAAAAAAAAAAAAAAAAAAAAAAAAAAAAAAAAAAAAAAAAAAAAAAAAAAAAAAAAAAAAAAAAAAAAAAAAAAAAAAAAAAAAAAAAAAAAAAAAAAAAAAAAAAAAAAAAAAAAAAAAAAAAAAAAAAAAAAAAAAAAAAAAAAAAAAAAAAAAAAAAAAAAAAAAAAAAAAAAAAAAAAAAAAAAAAAAAAAAAAAAAAAAAAAAAAAAAAAAAAAAAAAAAAAAAAAAAAAAAAAAAAAAAAAAAAAAAAAAAAAAAAAAAAAAAAAAAAAAAAAAAAAAAAAAAAAAAAAAAAAAAAAAAAAAAAAAAAAAAAAAAAAAAAAAAAAAAAAAAAAAAAAAAAAAAAAAAAAAAAAAAAAAAAAAAAAAAAAAAAAAAAAAAAAAAAAAAAAAAAAAAAAAAAAAAAAAAAAAAAAAAAAAAAAAAAAAAAAAAAAAAAAAAAAAAAAAAAAAAAAAAAAAAAAAAAAAAAAAAAAAAAAAAAAAAAAAAAAAAAAAAAAAAAAAAAAAAAAAAAAAAAAAAAAAAAAAAAAAAAAAAAAAAAAAAAAAAAAAAAAAAAAAAAAAAAAAAAAAAAAAAAAAAAAAAAAAAAAAAAAAAAAAAAAAAAAAAAAAAAAAAAAAAAAAAAAAAAAAAAAAAAAAAAAAAAAAAAAAAAAAAAAAAAAAAAAAAAAAAAAAAAAAAAAAAAAAAAAAAAAAAAAAAAAAAAAAAAAAAAAAAAAAAAAAAAAAAAAAAAAAAAAAAAAAAAAAAAAAAAAAAAAAAAAAAAAAAAAAAAAAAAAAAAAAAAAAAAAAAAAAAAAAAAAAAAAAAAAAAAAAAAAAAAAAAAAAAAAAAAAAAAAAAAAAAAAAAAAAAAAAAAAAAAAAAAAAAAAAAAAAAAAAAAAAAAAAAAAAAAAAAAAAAAAAAAAAAAAAAAAAAAAAAAAAAAAAAAAAAAAAAAAAAAAAAAAAAAAAAAAAAAAAAAAAAAAAAAAAAAAAAAAAAAAAAAAAAAAAAAAAAAAAAAAAAAAAAAAAAAAAAAAAAAAAAAAAAAAAAAAAAAAAAAAAAAAAAAAAAAAAAAAAAAAAAAAAAAAAAAAAAAAAAAAAAAAAAAAAAAAAAAAAAAAAAAAAAAAAAAAAAAAAAAAAAAAAAAAAAAAAAAAAAAAAAAAAAAAAAAAAAAAAAAAAAAAAAAAAAAAAAAAAAAAAAAAAAAAAAAAAAAAAAAAAAAAAAAAAAAAAAAAAAAAAAAAAAAAAAAAAAAAAAAAAAAAAAAAAAAAAAAAAAAAAAAAAAAAAAAAAAAAAAAAAAAAAAAAAAAAAAAAAAAAAAAAAAAAAAAAAAAAAAAAAAAAAAAAAAAAAAAAAAAAAAAAAAAAAAAAAAAAAAAAAAAAAAAAAAAAAAAAAAAAAAAAAAAAAAAAAAAAAAAAAAAAAAAAAAAAAAAAAAAAAAAAAAAAAAAAAAAAAAAAAAAAAAAAAAAAAAAAAAAAAAAAAAAAAAAAAAAAAAAAAAAAAAAAAAAAAAAAAAAAAAAAAAAAAAAAAAAAAAAAAAAAAAAAAAAAAAAAAAAAAAAAAAAAAAAAAAAAAAAAAAAAAAAAAAAAAAAAAAAAAAAAAAAAAAAAAAAAAAAAAAAAAAAAAAAAAAAAAAAAAAAAAAAAAAAAAAAAAAAAAAAAAAAAAAAAAAAAAAAAAAAAAAAAAAAAAAAAAAAAAAAAAAAAAAAAAAAAAAAAAAAAAAAAAAAAAAAAAAAAAAAAAAAAAAAAAAAAAAAAAAAAAAAAAAAAAAAAAAAAAAAAAAAAAAAAAAAAAAAAAAAAAAAAAAAAAAAAAAAAAAAAAAAAAAAAAAAAAAAAAAAAAAAAAAAAAAAAAAAAAAAAAAAAAAAAAAAAAAAAAAAAAAAAAAAAAAAAAAAAAAAAAAAAAAAAAAAAAAAAAAAAAAAAAAAAAAAAAAAAAAAAAAAAAAAAAAAAAAAAAAAAAAAAAAAAAAAAAAAAAAAAAAAAAAAAAAAAAAAAAAAAAAAAAAAAAAAAAAAAAAAAAAAAAAAAAAAAAAAAAAAAAAAAAAAAAAAAAAAAAAAAAAAAAAAAAAAAAAAAAAAAAAAAAAAAAAAAAAAAAAAAAAAAAAAAAAAAAAAAAAAAAAAAAAAAAAAAAAAAAAAAAAAAAAAAAAAAAAAAAAAAAAAAAAAAAAAAAAAAAAAAAAAAAAAAAAAAAAAAAAAAAAAAAAAAAAAAAAAAAAAAAAAAAAAAAAAAAAAAAAAAAAAAAAAAAAAAAAAAAAAAAAAAAAAAAAAAAAAAAAAAAAAAAAAAAAAAAAAAAAAAAAAAAAAAAAAAAAAAAAAAAAAAAAAAAAAAAAAAAAAAAAAAAAAAAAAAAAAAAAAAAAAAAAAAAAAAAAAAAAAAAAAAAAAAAAAAAAAAAAAAAAAAAAAAAAAAAAAAAAAAAAAAAAAAAAAAAAAAAAAAAAAAAAAAAAAAAAAAAAAAAAAAAAAAAAAAAAAAAAAAAAAAAAAAAAAAAAAAAAAAAAAAAAAAAAAAAAAAAAAAAAAAAAAAAAAAAAAAAAAAAAAAAAAAAAAAAAAAAAAAAAAAAAAAAAAAAAAAAAAAAAAAAAAAAAAAAAAAAAAAAAAAAAAAAAAAAAAAAAAAAAAAAAAAAAAAAAAAAAAAAAAAAAAAAAAAAAAAAAAAAAAAAAAAAAAAAAAAAAAAAAAAAAAAAAAAAAAAAAAAAAAAAAAAAAAAAAAAAAAAAAAAAAAAAAAAAAAAAAAAAAAAAAAAAAAAAAAAAAAAAAAAAAAAAAAAAAAAAAAAAAAAAAAAAAAAAAAAAAAAAAAAAAAAAAAAAAAAAAAAAAAAAAAAAAAAAAAAAAAAAAAAAAAAAAAAAAAAAAAAAAAAAAAAAAAAAAAAAAAAAAAAAAAAAAAAAAAAAAAAAAAAAAAAAAAAAAAAAAAAAAAAAAAAAAAAAAAAAAAAAAAAAAAAAAAAAAAAAAAAAAAAAAAAAAAAAAAAAAAAAAAAAAAAAAAAAAAAAAAAAAAAAAAAAAAAAAAAAAAAAAAAAAAAAAAAAAAAAAAAAAAAAAAAAAAAAAAAAAAAAAAAAAAAAAAAAAAAAAAAAAAAAAAAAAAAAAAAAAAAAAAAAAAAAAAAAAAAAAAAAAAAAAAAAAAAAAAAAAAAAAAAAAAAAAAAAAAAAAAAAAAAAAAAAAAAAAAAAAAAAAAAAAAAAAAAAAAAAAAAAAAAAAAAAAAAAAAAAAAAAAAAAAAAAAAAAAAAAAAAAAAAAAAAAAAAAAAAAAAAAAAAAAAAAAAAAAAAAAAAAAAAAAAAAAAAAAAAAAAAAAAAAAAAAAAAAAAAAAAAAAAAAAAAAAAAAAAAAAAAAAAAAAAAAAAAAAAAAAAAAAAAAAAAAAAAAAAAAAAAAAAAAAAAIPGMABCLJNJDKK

"Surface View" fire

"Surface View" setViewerMask 16382

"Surface View" setShadowStyle 0

"Surface View" setPickable 1

set hideNewModules 0

%Apply Filter By Measure module to filter objects into a label or binary image given a target measure and a filter criterion

create measurefilter "Filter By Measure"

"Filter By Measure" setIconPosition 160 1126

"Filter By Measure" setVar "CustomHelp" {measurefilter.html}

"Filter By Measure" interpretation setValue 0

"Filter By Measure" outputLocation setIndex 0 0

"Filter By Measure" inputImage connect "AD.Optimized.view3.thresholded.am"

"Filter By Measure" inputIntensityImage connect "AD.Optimized.view3.mult.am"

labelMeasure setAttributes feret2d 0 18 36 54 72 90 108 126 144 162

labelMeasure setAttributes feret3d 31

labelMeasure setAttributes cooccurrence 0 0

labelMeasure setAttributes histogram 1 0 255 1

labelMeasure setAttributes quantile 0.1 0.1 0.1 0.1 0.1 0.1

labelMeasure setAttributes breadth3d 10

labelMeasure setAttributes neighborCount 0 5 0

"Filter By Measure" measure setState {Area3d}

"Filter By Measure" measureFilterType setIndex 0 0

"Filter By Measure" numberOfPatterns setMinMax 0 0 2147483648

"Filter By Measure" numberOfPatterns setValue 0 500

"Filter By Measure" applyTransformToResult 1

"Filter By Measure" fire

"Filter By Measure" setViewerMask 16383

"Filter By Measure" setPickable 1

set hideNewModules 0

[ load ${SCRIPTDIR}/AD.Optimized.view3.labels.am ] setLabel "AD.Optimized.view3.labels.am"

"AD.Optimized.view3.labels.am" setIconPosition 20 1486

"AD.Optimized.view3.labels.am" master connect "Filter By Measure" "ImgOut" 0

"AD.Optimized.view3.labels.am" sharedColormap connect "labels.am"

"AD.Optimized.view3.labels.am" sharedColormap setDefaultColor 0.8 0.8 0.8

"AD.Optimized.view3.labels.am" sharedColormap setDefaultAlpha 0.500000

"AD.Optimized.view3.labels.am" sharedColormap activateLocalRange 1

"AD.Optimized.view3.labels.am" sharedColormap setLocalMinMax 1.000000 8.000000

"AD.Optimized.view3.labels.am" sharedColormap enableAlpha 1

"AD.Optimized.view3.labels.am" sharedColormap enableAlphaToggle 1

"AD.Optimized.view3.labels.am" sharedColormap setAutoAdjustRangeMode 1

"AD.Optimized.view3.labels.am" fire

"AD.Optimized.view3.labels.am" primary setIndex 0 0

"AD.Optimized.view3.labels.am" fire

"AD.Optimized.view3.labels.am" setViewerMask 16383

set hideNewModules 0

%Apply Volume Rendering module to allow for real-time rendering

create HxVolumeRenderingSettings "Volume Rendering Settings 3"

"Volume Rendering Settings 3" setViewerMask 0

"Volume Rendering Settings 3" setIconPosition 651 1486

"Volume Rendering Settings 3" setVar "CustomHelp" {HxVolumeRenderingSettings}

set hideNewModules 0

"Volume Rendering Settings 3" data connect "AD.Optimized.view3.labels.am"

"Volume Rendering Settings 3" fire

"Volume Rendering Settings 3" rendering setValue 0

"Volume Rendering Settings 3" fire

"Volume Rendering Settings 3" interpolationAdvanced setValue 0

"Volume Rendering Settings 3" composition setValue 0

"Volume Rendering Settings 3" moveLowResolutionScale setMinMax 1 10

"Volume Rendering Settings 3" moveLowResolutionScale setButtons 1

"Volume Rendering Settings 3" moveLowResolutionScale setEditButton 1

"Volume Rendering Settings 3" moveLowResolutionScale setIncrement 1

"Volume Rendering Settings 3" moveLowResolutionScale setValue 3

"Volume Rendering Settings 3" moveLowResolutionScale setSubMinMax 1 10

"Volume Rendering Settings 3" samplingQuality setMinMax 0 2

"Volume Rendering Settings 3" samplingQuality setButtons 0

"Volume Rendering Settings 3" samplingQuality setEditButton 1

"Volume Rendering Settings 3" samplingQuality setIncrement 0.133333

"Volume Rendering Settings 3" samplingQuality setValue 1

"Volume Rendering Settings 3" samplingQuality setSubMinMax 0 2

"Volume Rendering Settings 3" opacityThreshold setMinMax 0 1

"Volume Rendering Settings 3" opacityThreshold setButtons 0

"Volume Rendering Settings 3" opacityThreshold setEditButton 1

"Volume Rendering Settings 3" opacityThreshold setIncrement 0.05

"Volume Rendering Settings 3" opacityThreshold setValue 0

"Volume Rendering Settings 3" opacityThreshold setSubMinMax 0 1

"Volume Rendering Settings 3" optimizations setValue 0 0

"Volume Rendering Settings 3" optimizations setToggleVisible 0 1

"Volume Rendering Settings 3" optimizations setValue 1 1

"Volume Rendering Settings 3" optimizations setToggleVisible 1 1

"Volume Rendering Settings 3" optimizations setValue 2 0

"Volume Rendering Settings 3" optimizations setToggleVisible 2 1

"Volume Rendering Settings 3" quality setValue 1

"Volume Rendering Settings 3" fire

"Volume Rendering Settings 3" artifactsReduction setValue 0 0

"Volume Rendering Settings 3" artifactsReduction setToggleVisible 0 1

"Volume Rendering Settings 3" artifactsReduction setValue 1 0

"Volume Rendering Settings 3" artifactsReduction setToggleVisible 1 1

"Volume Rendering Settings 3" lighting setState {item 0 1 item 1 1 }

"Volume Rendering Settings 3" gradient setState {item 0 1 item 2 9.99999974737875e-05 }

"Volume Rendering Settings 3" effects setValue 0 0

"Volume Rendering Settings 3" effects setToggleVisible 0 1

"Volume Rendering Settings 3" effects setValue 1 0

"Volume Rendering Settings 3" effects setToggleVisible 1 1

"Volume Rendering Settings 3" effects setValue 2 0

"Volume Rendering Settings 3" effects setToggleVisible 2 1

"Volume Rendering Settings 3" effects setValue 3 1

"Volume Rendering Settings 3" effects setToggleVisible 3 1

"Volume Rendering Settings 3" edgeEnhancement setMinMax 0 -3.40282346638529e+038 3.40282346638529e+038

"Volume Rendering Settings 3" edgeEnhancement setValue 0 9.99999974737875e-005

"Volume Rendering Settings 3" edge2D setState {item 1 0.100000001490116 item 3 0.100000001490116 item 5 1 }

"Volume Rendering Settings 3" boundaryOpacity setMinMax 0 -3.40282346638529e+038 3.40282346638529e+038

"Volume Rendering Settings 3" boundaryOpacity setValue 0 2.5

"Volume Rendering Settings 3" boundaryOpacity setMinMax 1 -3.40282346638529e+038 3.40282346638529e+038

"Volume Rendering Settings 3" boundaryOpacity setValue 1 2.5

"Volume Rendering Settings 3" material setIndex 0 4

"Volume Rendering Settings 3" lightingStyle setIndex 0 1

"Volume Rendering Settings 3" toneMapping setIndex 0 0

"Volume Rendering Settings 3" depthOfField setValue 0

"Volume Rendering Settings 3" blurFactor setMinMax 0.00999999977648258 1

"Volume Rendering Settings 3" blurFactor setButtons 0

"Volume Rendering Settings 3" blurFactor setEditButton 1

"Volume Rendering Settings 3" blurFactor setIncrement 0.066

"Volume Rendering Settings 3" blurFactor setValue 0.01

"Volume Rendering Settings 3" blurFactor setSubMinMax 0.00999999977648258 1

"Volume Rendering Settings 3" specularColor setMinMax 0 1

"Volume Rendering Settings 3" specularColor setButtons 0

"Volume Rendering Settings 3" specularColor setEditButton 1

"Volume Rendering Settings 3" specularColor setIncrement 0.1

"Volume Rendering Settings 3" specularColor setValue 0.4

"Volume Rendering Settings 3" specularColor setSubMinMax 0 1

"Volume Rendering Settings 3" shininess setMinMax 0 1

"Volume Rendering Settings 3" shininess setButtons 0

"Volume Rendering Settings 3" shininess setEditButton 1

"Volume Rendering Settings 3" shininess setIncrement 0.1

"Volume Rendering Settings 3" shininess setValue 0.4

"Volume Rendering Settings 3" shininess setSubMinMax 0 1

"Volume Rendering Settings 3" fire

"Volume Rendering Settings 3" setViewerMask 16383

"Volume Rendering Settings 3" setPickable 1

set hideNewModules 0

create HxVolumeRender2 "Volume Rendering 3"

"Volume Rendering 3" setIconPosition 651 1512

"Volume Rendering 3" setVar "CustomHelp" {HxVolumeRender2}

"Volume Rendering 3" data connect "AD.Optimized.view3.labels.am"

"Volume Rendering 3" volumeRenderingSettings connect "Volume Rendering Settings 3"

"Volume Rendering 3" fire

"Volume Rendering 3" colormap connect "labels.am"

"Volume Rendering 3" colormap setDefaultColor 1 1 1

"Volume Rendering 3" colormap setDefaultAlpha 0.500000

"Volume Rendering 3" colormap activateLocalRange 1

"Volume Rendering 3" colormap setLocalMinMax 1.000000 8.000000

"Volume Rendering 3" colormap enableAlpha 1

"Volume Rendering 3" colormap enableAlphaToggle 1

"Volume Rendering 3" colormap setAutoAdjustRangeMode 1

"Volume Rendering 3" fire

"Volume Rendering 3" colormapLookup setValue 2

"Volume Rendering 3" gamma setMinMax 0.100000001490116 8

"Volume Rendering 3" gamma setButtons 0

"Volume Rendering 3" gamma setEditButton 1

"Volume Rendering 3" gamma setIncrement 0.526667

"Volume Rendering 3" gamma setValue 3

"Volume Rendering 3" gamma setSubMinMax 0.100000001490116 8

"Volume Rendering 3" alphaScale setMinMax 0 1

"Volume Rendering 3" alphaScale setButtons 0

"Volume Rendering 3" alphaScale setEditButton 1

"Volume Rendering 3" alphaScale setIncrement 0.1

"Volume Rendering 3" alphaScale setValue 1

"Volume Rendering 3" alphaScale setSubMinMax 0 1

"Volume Rendering 3" channelSelector setState {2 }

"Volume Rendering 3" fire

"Volume Rendering 3" setViewerMask 16382

"Volume Rendering 3" setPickable 1

set hideNewModules 0

%Apply Generate Surface module to compute a triangular approximation of the interfaces between create HxGMC "Generate Surface 2"

"Generate Surface 2" setIconPosition 160 1522

"Generate Surface 2" setVar "CustomHelp" {HxGMC}

"Generate Surface 2" data connect "AD.Optimized.view3.labels.am"

"Generate Surface 2" fire

"Generate Surface 2" smoothing setIndex 0 3

"Generate Surface 2" smoothingExtent setMinMax 1 9

"Generate Surface 2" smoothingExtent setButtons 0

"Generate Surface 2" smoothingExtent setEditButton 1

"Generate Surface 2" smoothingExtent setIncrement 0.533333

"Generate Surface 2" smoothingExtent setValue 5

"Generate Surface 2" smoothingExtent setSubMinMax 1 9

"Generate Surface 2" options setState {item 0 0 item 1 0 }

"Generate Surface 2" borderOnOff setValue 1

"Generate Surface 2" algorithmMode setIndex 0 0

"Generate Surface 2" borderSettings setValue 0 1

"Generate Surface 2" borderSettings setToggleVisible 0 1

"Generate Surface 2" borderSettings setValue 1 0

"Generate Surface 2" borderSettings setToggleVisible 1 1

"Generate Surface 2" borderSettings setValue 2 0

"Generate Surface 2" borderSettings setToggleVisible 2 1

"Generate Surface 2" borderSettings setValue 3 0

"Generate Surface 2" borderSettings setToggleVisible 3 0

"Generate Surface 2" materialList setIndex 0 0

"Generate Surface 2" smoothMaterial setIndex 0 0

"Generate Surface 2" applyTransformToResult 1

"Generate Surface 2" fire

"Generate Surface 2" setViewerMask 16383

"Generate Surface 2" setPickable 1

set hideNewModules 0

[ load ${SCRIPTDIR}/AD.Optimized.view2.surf.am ] setLabel "AD.Optimized.view2.surf.am"

"AD.Optimized.view2.surf.am" setIconPosition 20 1558

"AD.Optimized.view2.surf.am" master connect "Generate Surface 2" "result" 0

"AD.Optimized.view2.surf.am" fire

"AD.Optimized.view2.surf.am" LevelOfDetail setMinMax -1 -1

"AD.Optimized.view2.surf.am" LevelOfDetail setButtons 1

"AD.Optimized.view2.surf.am" LevelOfDetail setEditButton 1

"AD.Optimized.view2.surf.am" LevelOfDetail setIncrement 1

"AD.Optimized.view2.surf.am" LevelOfDetail setValue -1

"AD.Optimized.view2.surf.am" LevelOfDetail setSubMinMax -1 -1

"AD.Optimized.view2.surf.am" fire

"AD.Optimized.view2.surf.am" setViewerMask 16383

set hideNewModules 0

%Apply Surface View module to visualize triangular surfaces, i.e., data objects of type Surface

create HxDisplaySurface "Surface View 2"

"Surface View 2" setIconPosition 807 1558

"Surface View 2" setVar "CustomHelp" {HxDisplaySurface}

"Surface View 2" data connect "AD.Optimized.view2.surf.am"

"Surface View 2" colormap disconnect

"Surface View 2" colormap setDefaultColor 0.1 1 0.90625

"Surface View 2" colormap setDefaultAlpha 0.500000

"Surface View 2" colormap activateLocalRange 1

"Surface View 2" colormap setLocalMinMax 0.000000 1.000000

"Surface View 2" colormap enableAlpha 1

"Surface View 2" colormap enableAlphaToggle 1

"Surface View 2" colormap setAutoAdjustRangeMode 1

"Surface View 2" fire

"Surface View 2" drawStyle setValue 1

"Surface View 2" fire

"Surface View 2" drawStyle setSpecularLighting 1

"Surface View 2" drawStyle setTexture 1

"Surface View 2" drawStyle setAlphaMode 1

"Surface View 2" drawStyle setCullingMode 0

"Surface View 2" drawStyle setNormalBinding 0

"Surface View 2" drawStyle setSortingMode 1

"Surface View 2" drawStyle setLineWidth 0.000000

"Surface View 2" drawStyle setOutlineColor 0 0 0.2

"Surface View 2" textureWrap setIndex 0 1

"Surface View 2" selectionMode setIndex 0 0

"Surface View 2" patch setMinMax 0 2

"Surface View 2" patch setButtons 1

"Surface View 2" patch setEditButton 1

"Surface View 2" patch setIncrement 1

"Surface View 2" patch setValue 0

"Surface View 2" patch setSubMinMax 0 2

"Surface View 2" boundaryId setIndex 0 -1

"Surface View 2" materials setIndex 0 1

"Surface View 2" materials setIndex 1 0

"Surface View 2" colorMode setIndex 0 5

"Surface View 2" baseTrans setMinMax 0 1

"Surface View 2" baseTrans setButtons 0

"Surface View 2" baseTrans setEditButton 1

"Surface View 2" baseTrans setIncrement 0.1

"Surface View 2" baseTrans setValue 0.8

"Surface View 2" baseTrans setSubMinMax 0 1

"Surface View 2" VRMode setIndex 0 0

"Surface View 2" colorFieldMappingType setValue 0

"Surface View 2" setLighting 1

"Surface View 2" fire

"Surface View 2" hideBox 1

"Surface View 2" selectTriangles zab HIJMOMMBABABAAAAAAIAJAPPKLOLAIAKAAAAAAAAAAAAAAAAAAAAAAAAAAAAAAAAAAAAAAAAAAAAAAAAAAAAAAAAAAAAAAAAAAAAAAAAAAAAAAAAAAAAAAAAAAAAAAAAAAAAAAAAAAAAAAAAAAAAAAAAAAAAAAAAAAAAAAAAAAAAAAAAAAAAAAAAAAAAAAAAAAAAAAAAAAAAAAAAAAAAAAAAAAAAAAAAAAAAAAAAAAAAAAAAAAAAAAAAAAAAAAAAAAAAAAAAAAAAAAAAAAAAAAAAAAAAAAAAAAAAAAAAAAAAAAAAAAAAAAAAAAAAAAAAAAAAAAAAAAAAAAAAAAAAAAAAAAAAAAAAAAAAAAAAAAAAAAAAAAAAAAAAAAAAAAAAAAAAAAAAAAAAAAAAAAAAAAAAAAAAAAAAAAAAAAAAAAAAAAAAAAAAAAAAAAAAAAAAAAAAAAAAAAAAAAAAAAAAAAAAAAAAAAAAAAAAAAAAAAAAAAAAAAAAAAAAAAAAAAAAAAAAAAAAAAAAAAAAAAAAAAAAAAAAAAAAAAAAAAAAAAAAAAAAAAAAAAAAAAAAAAAAAAAAAAAAAAAAAAAAAAAAAAAAAAAAAAAAAAAAAAAAAAAAAAAAAAAAAAAAAAAAAAAAAAAAAAAAAAAAAAAAAAAAAAAAAAAAAAAAAAAAAAAAAAAAAAAAAAAAAAAAAAAAAAAAAAAAAAAAAAAAAAAAAAAAAAAAAAAAAAAAAAAAAAAAAAAAAAAAAAAAAAAAAAAAAAAAAAAAAAAAAAAAAAAAAAAAAAAAAAAAAAAAAAAAAAAAAAAAAAAAAAAAAAAAAAAAAAAAAAAAAAAAAAAAAAAAAAAAAAAAAAAAAAAAAAAAAAAAAAAAAAAAAAAAAAAAAAAAAAAAAAAAAAAAAAAAAAAAAAAAAAAAAAAAAAAAAAAAAAAAAAAAAAAAAAAAAAAAAAAAAAAAAAAAAAAAAAAAAAAAAAAAAAAAAAAAAAAAAAAAAAAAAAAAAAAAAAAAAAAAAAAAAAAAAAAAAAAAAAAAAAAAAAAAAAAAAAAAAAAAAAAAAAAAAAAAAAAAAAAAAAAAAAAAAAAAAAAAAAAAAAAAAAAAAAAAAAAAAAAAAAAAAAAAAAAAAAAAAAAAAAAAAAAAAAAAAAAAAAAAAAAAAAAAAAAAAAAAAAAAAAAAAAAAAAAAAAAAAAAAAAAAAAAAAAAAAAAAAAAAAAAAAAAAAAAAAAAAAAAAAAAAAAAAAAAAAAAAAAAAAAAAAAAAAAAAAAAAAAAAAAAAAAAAAAAAAAAAAAAAAAAAAAAAAAAAAAAAAAAAAAAAAAAAAAAAAAAAAAAAAAAAAAAAAAAAAAAAAAAAAAAAAAAAAAAAAAAAAAAAAAAAAAAAAAAAAAAAAAAAAAAAAAAAAAAAAAAAAAAAAAAAAAAAAAAAAAAAAAAAAAAAAAAAAAAAAAAAAAAAAAAAAAAAAAAAAAAAAAAAAAAAAAAAAAAAAAAAAAAAAAAAAAAAAAAAAAAAAAAAAAAAAAAAAAAAAAAAAAAAAAAAAAAAAAAAAAAAAAAAAAAAAAAAAAAAAAAAAAAAAAAAAAAAAAAAAAAAAAAAAAAAAAAAAAAAAAAAAAAAAAAAAAAAAAAAAAAAAAAAAAAAAAAAAAAAAAAAAAAAAAAAAAAAAAAAAAAAAAAAAAAAAAAAAAAAAAAAAAAAAAAAAAAAAAAAAAAAAAAAAAAAAAAAAAAAAAAAAAAAAAAAAAAAAAAAAAAAAAAAAAAAAAAAAAAAAAAAAAAAAAAAAAAAAAAAAAAAAAAAAAAAAAAAAAAAAAAAAAAAAAAAAAAAAAAAAAAAAAAAAAAAAAAAAAAAAAAAAAAAAAAAAAAAAAAAAAAAAAAAAAAAAAAAAAAAAAAAAAAAAAAAAAAAAAAAAAAAAAAAAAAAAAAAAAAAAAAAAAAAAAAAAAAAAAAAAAAAAAAAAAAAAAAAAAAAAAAAAAAAAAAAAAAAAAAAAAAAAAAAAAAAAAAAAAAAAAAAAAAAAAAAAAAAAAAAAAAAAAAAAAAAAAAAAAAAAAAAAAAAAAAAAAAAAAAAAAAAAAAAAAAAAAAAAAAAAAAAAAAAAAAAAAAAAAAAAAAAAAAAAAAAAAAAAAAAAAAAAAAAAAAAAAAAAAAAAAAAAAAAAAAAAAAAAAAAAAAAAAAAAAAAAAAAAAAAAAAAAAAAAAAAAAAAAAAAAAAAAAAAAAAAAAAAAAAAAAAAAAAAAAAAAAAAAAAAAAAAAAAAAAAAAAAAAAAAAAAAAAAAAAAAAAAAAAAAAAAAAAAAAAAAAAAAAAAAAAAAAAAAAAAAAAAAAAAAAAAAAAAAAAAAAAAAAAAAAAAAAAAAAAAAAAAAAAAAAAAAAAAAAAAAAAAAAAAAAAAAAAAAAAAAAAAAAAAAAAAAAAAAAAAAAAAAAAAAAAAAAAAAAAAAAAAAAAAAAAAAAAAAAAAAAAAAAAAAAAAAAAAAAAAAAAAAAAAAAAAAAAAAAAAAAAAAAAAAAAAAAAAAAAAAAAAAAAAAAAAAAAAAAAAAAAAAAAAAAAAAAAAAAAAAAAAAAAAAAAAAAAAAAAAAAAAAAAAAAAAAAAAAAAAAAAAAAAAAAAAAAAAAAAAAAAAAAAAAAAAAAAAAAAAAAAAAAAAAAAAAAAAAAAAAAAAAAAAAAAAAAAAAAAAAAAAAAAAAAAAAAAAAAAAAAAAAAAAAAAAAAAAAAAAAAAAAAAAAAAAAAAAAAAAAAAAAAAAAAAAAAAAAAAAAAAAAAAAAAAAAAAAAAAAAAAAAAAAAAAAAAAAAAAAAAAAAAAAAAAAAAAAAAAAAAAAAAAAAAAAAAAAAAAAAAAAAAAAAAAAAAAAAAAAAAAAAAAAAAAAAAAAAAAAAAAAAAAAAAAAAAAAAAAAAAAAAAAAAAAAAAAAAAAAAAAAAAAAAAAAAAAAAAAAAAAAAAAAAAAAAAAAAAAAAAAAAAAAAAAAAAAAAAAAAAAAAAAAAAAAAAAAAAAAAAAAAAAAAAAAAAAAAAAAAAAAAAAAAAAAAAAAAAAAAAAAAAAAAAAAAAAAAAAAAAAAAAAAAAAAAAAAAAAAAAAAAAAAAAAAAAAAAAAAAAAAAAAAAAAAAAAAAAAAAAAAAAAAAAAAAAAAAAAAAAAAAAAAAAAAAAAAAAAAAAAAAAAAAAAAAAAAAAAAAAAAAAAAAAAAAAAAAAAAAAAAAAAAAAAAAAAAAAAAAAAAAAAAAAAAAAAAAAAAAAAAAAAAAAAAAAAAAAAAAAAAAAAAAAAAAAAAAAAAAAAAAAAAAAAAAAAAAAAAAAAAAAAAAAAAAAAAAAAAAAAAAAAAAAAAAAAAAAAAAAAAAAAAAAAAAAAAAAAAAAAAAAAAAAAAAAAAAAAAAAAAAAAAAAAAAAAAAAAAAAAAAAAAAAAAAAAAAAAAAAAAAAAAAAAAAAAAAAAAAAAAAAAAAAAAAAAAAAAAAAAAAAAAAAAAAAAAAAAAAAAAAAAAAAAAAAAAAAAAAAAAAAAAAAAAAAAAAAAAAAAAAAAAAAAAAAAAAAAAAAAAAAAAAAAAAAAAAAAAAAAAAAAAAAAAAAAAAAAAAAAAAAAAAAAAAAAAAAAAAAAAAAAAAAAAAAAAAAAAAAAAAAAAAAAAAAAAAAAAAAAAAAAAAAAAAAAAAAAAAAAAAAAAAAAAAAAAAAAAAAAAAAAAAAAAAAAAAAAAAAAAAAAAAAAAAAAAAAAAAAAAAAAAAAAAAAAAAAAAAAAAAAAAAAAAAAAAAAAAAAAAAAAAAAAAAAAAAAAAAAAAAAAAAAAAAAAAAAAAAAAAAAAAAAAAAAAAAAAAAAAAAAAAAAAAAAAAAAAAAAAAAAAAAAAAAAAAAAAAAAAAAAAAAAAAAAAAAAAAAAAAAAAAAAAAAAAAAAAAAAAAAAAAAAAAAAAAAAAAAAAAAAAAAAAAAAAAAAAAAAAAAAAAAAAAAAAAAAAAAAAAAAAAAAAAAAAAAAAAAAAAAAAAAAAAAAAAAAAAAAAAAAAAAAAAAAAAAAAAAAAAAAAAAAAAAAAAAAAAAAAAAAAAAAAAAAAAAAAAAAAAAAAAAAAAAAAAAAAAAAAAAAAAAAAAAAAAAAAAAAAAAAAAAAAAAAAAAAAAAAAAAAAAAAAAAAAAAAAAAAAAAAAAAAAAAAAAAAAAAAAAAAAAAAAAAAAAAAAAAAAAAAAAAAAAAAAAAAAAAAAAAAAAAAAAAAAAAAAAAAAAAAAAAAAAAAAAAAAAAAAAAAAAAAAAAAAAAAAAAAAAAAAAAAAAAAAAAAAAAAAAAAAAAAAAAAAAAAAAAAAAAAAAAAAAAAAAAAAAAAAAAAAAAAAAAAAAAAAAAAAAAAAAAAAAAAAAAAAAAAAAAAAAAAAAAAAAAAAAAAAAAAAAAAAAAAAAAAAAAAAAAAAAAAAAAAAAAAAAAAAAAAAAAAAAAAAAAAAAAAAAAAAAAAAAAAAAAAAAAAAAAAAAAAAAAAAAAAAAAAAAAAAAAAAAAAAAAAAAAAAAAAAAAAAAAAAAAAAAAAAAAAAAAAAAAAAAAAAAAAAAAAAAAAAAAAAAAAAAAAAAAAAAAAAAAAAAAAAAAAAAAAAAAAAAAAAAAAAAAAAAAAAAAAAAAAAAAAAAAAAAAAAAAAAAAAAAAAAAAAAAAAAAAAAAAAAAAAAAAAAAAAAAAAAAAAAAAAAAAAAAAAAAAAAAAAAAAAAAAAAAAAAAAAAAAAAAAAAAAAAAAAAAAAAAAAAAAAAAAAAAAAAAAAAAAAAAAAAAAAAAAAAAAAAAAAAAAAAAAAAAAAAAAAAAAAAAAAAAAAAAAAAAAAAAAAAAAAAAAAAAAAAAAAAAAAAAAAAAAAAAAAAAAAAAAAAAAAAAAAAAAAAAAAAAAAAAAAAAAAAAAAAAAAAAAAAAAAAAAAAAAAAAAAAAAAAAAAAAAAAAAAAAAAAAAAAAAAAAAAAAAAAAAAAAAAAAAAAAAAAAAAAAAAAAAAAAAAAAAAAAAAAAAAAAAAAAAAAAAAAAAAAAAAAAAAAAAAAAAAAAAAAAAAAAAAAAAAAAAAAAAAAAAAAAAAAAAAAAAAAAAAAAAAAAAAAAAAAAAAAAAAAAAAAAAAAAAAAAAAAAAAAAAAAAAAAAAAAAAAAAAAAAAAAAAAAAAAAAAAAAAAAAAAAAAAAAAAAAAAAAAAAAAAAAAAAAAAAAAAAAAAAAAAAAAAAAAAAAAAAAAAAAAAAAAAAAAAAAAAAAAAAAAAAAAAAAAAAAAAAAAAAAAAAAAAAAAAAAAAAAAAAAAAAAAAAAAAAAAAAAAAAAAAAAAAAAAAAAAAAAAAAAAAAAAAAAAAAAAAAAAAAAAAAAAAAAAAAAAAAAAAAAAAAAAAAAAAAAAAAAAAAAAAAAAAAAAAAAAAAAAAAAAAAAAAAAAAAAAAAAAAAAAAAAAAAAAAAAAAAAAAAAAAAAAAAAAAAAAAAAAAAAAAAAAAAAAAAAAAAAAAAAAAAAAAAAAAAAAAAAAAAAAAAAAAAAAAAAAAAAAAAAAAAAAAAAAAAAAAAAAAAAAAAAAAAAAAAAAAAAAAAAAAAAAAAAAAAAAAAAAAAAAAAAAAAAAAAAAAAAAAAAAAAAAAAAAAAAAAAAAAAAAAAAAAAAAAAAAAAAAAAAAAAAAAAAAAAAAAAAAAAAAAAAAAAAAAAAAAAAAAAAAAAAAAAAAAAAAAAAAAAAAAAAAAAAAAAAAAAAAAAAAAAAAAAAAAAAAAAAAAAAAAAAAAAAAAAAAAAAAAAAAAAAAAAAAAAAAAAAAAAAAAAAAAAAAAAAAAAAAAAAAAAAAAAAAAAAAAAAAAAAAAAAAAAAAAAAAAAAAAAAAAAAAAAAAAAAAAAAAAAAAAAAAAAAAAAAAAAAAAAAAAAAAAAAAAAAAAAAAAAAAAAAAAAAAAAAAAAAAAAAAAAAAAAAAAAAAAAAAAAAAAAAAAAAAAAAAAAAAAAAAAAAAAAAAAAAAAAAAAAAAAAAAAAAAAAAAAAAAAAAAAAAAAAAAAAAAAAAAAAAAAAAAAAAAAAAAAAAAAAAAAAAAAAAAAAAAAAAAAAAAAAAAAAAAAAAAAAAAAAAAAAAAAAAAAAAAAAAAAAAAAAAAAAAAAAAAAAAAAAAAAAAAAAAAAAAAAAAAAAAAAAAAAAAAAAAAAAAAAAAAAAAAAAAAAAAAAAAAAAAAAAAAAAAAAAAAAAAAAAAAAAAAAAAAAAAAAAAAAAAAAAAAAAAAAAAAAAAAAAAAAAAAAAAAAAAAAAAAAAAAAAAAAAAAAAAAAAAAAAAAAAAAAAAAAAAAAAAAAAAAAAAAAAAAAAAAAAAAAAAAAAAAAAAAAAAAAAAAAAAAAAAAAAAAAAAAAAAAAAAAAAAAAAAAAAAAAAAAAAAAAAAAAAAAAAAAAAAAAAAAAAAAAAAAAAAAAAAAAAAAAAAAAAAAAAAAAAAAAAAAAAAAAAAAAAAAAAAAAAAAAAAAAAAAAAAAAAAAAAAAAAAAAAAAAAAAAAAAAAAAAAAAAAAAAAAAAAAAAAAAAAAAAAAAAAAAAAAAAAAAAAAAAAAAAAAAAAAAAAAAAAAAAAAAAAAAAAAAAAAAAAAAAAAAAAAAAAAAAAAAAAAAAAAAAAAAAAAAAAAAAAAAAAAAAAAAAAAAAAAAAAAAAAAAAAAAAAAAAAAAAAAAAAAAAAAAAAAAAAAAAAAAAAAAAAAAAAAAAAAAAAAAAAAAAAAAAAAAAAAAAAAAAAAAAAAAAAAAAAAAAAAAAAAAAAAAAAAAAAAAAAAAAAAAAAAAAAAAAAAAAAAAAAAAAAAAAAAAAAAAAAAAAAAAAAAAAAAAAAAAAAAAAAAAAAAAAAAAAAAAAAAAAAAAAAAAAAAAAAAAAAAAAAAAAAAAAAAAAAAAAAAAAAAAAAAAAAAAAAAAAAAAAAAAAAAAAAAAAAAAAAAAAAAAAAAAAAAAAAAAAAAAAAAAAAAAAAAAAAAAAAAAAAAAAAAAAAAAAAAAAAAAAAAAAAAAAAAAAAAAAAAAAAAAAAAAAAAAAAAAAAAAAAAAAAAAAAAAAAAAAAAAAAAAAAAAAAAAAAAAAAAAAAAAAAAAAAAAAAAAAAAAAAAAAAAAAAAAAAAAAAAAAAAAAAAAAAAAAAAAAAAAAAAAAAAAAAAAAAAAAAAAAAAAAAAAAAAAAAAAAAAAAAAAAAAAAAAAAAAAAAAAAAAAAAAAAAAAAAAAAAAAAAAAAAAAAAAAAAAAAAAAAAAAAAAAAAAAAAAAAAAAAAAAAAAAAAAAAAAAAAAAAAAAAAAAAAAAAAAAAAAAAAAAAAAAAAAAAAAAAAAAAAAAAAAAAAAAAAAAAAAAAAAAAAAAAAAAAAAAAAAAAAAAAAAAAAAAAAAAAAAAAAAAAAAAAAAAAAAAAAAAAAAAAAAAAAAAAAAAAAAAAAAAAAAAAAAAAAAAAAAAAAAAAAAAAAAAAAAAAAAAAAAAAAAAAAAAAAAAAAAAAAAAAAAAAAAAAAAAAAAAAAAAAAAAAAAAAAAAAAAAAAAAAAAAAAAAAAAAAAAAAAAAAAAAAAAAAAAAAAAAAAAAAAAAAAAAAAAAAAAAAAAAAAAAAAAAAAAAAAAAAAAAAAAAAAAAAAAAAAAAAAAAAAAAAAAAAAAAAAAAAAAAAAAAAAAAAAAAAAAAAAAAAAAAAAAAAAAAAAAAAAAAAAAAAAAAAAAAAAAAAAAAAAAAAAAAAAAAAAAAAAAAAAAAAAAAAAAAAAAAAAAAAAAAAAAAAAAAAAAAAAAAAAAAAAAAAAAAAAAAAAAAAAAAAAAAAAAAAAAAAAAAAAAAAAAAAAAAAAAAAAAAAAAAAAAAAAAAAAAAAAAAAAAAAAAAAAAAAAAAAAAAAAAAAAAAAAAAAAAAAAAAAAAAAAAAAAAAAAAAAAAAAAAAAAAAAAAAAAAAAAAAAAAAAAAAAAAAAAAAAAAAAAAAAAAAAAAAAAAAAAAAAAAAAAAAAAAAAAAAAAAAAAAAAAAAAAAAAAAAAAAAAAAAAAAAAAAAAAAAAAAAAAAAAAAAAAAAAAAAAAAAAAAAAAAAAAAAAAAAAAAAAAAAAAAAAAAAAAAAAAAAAAAAAAAAAAAAAAAAAAAAAAAAAAAAAAAAAAAAAAAAAAAAAAAAAAAAAAAAAAAAAAAAAAAAAAAAAAAAAAAAAAAAAAAAAAAAAAAAAAAAAAAAAAAAAAAAAAAAAAAAAAAAAAAAAAAAAAAAAAAAAAAAAAAAAAAAAAAAAAAAAAAAIANLIDADBKAAAABIAEEAGOPGMPOMBDLMABAAAAAAAAAAAAAAAAAAAAAAAAAAAAAAAAAAAAAAAAAAAAAAAAAAAAAAAAAAAAAAAAAAAAAAAAAAAAAAAAAAAAAAAAAAAAAAAAAAAAAAAAAAAAAAAAAAAAAAAAAAAAAAAAAAAAAAAAAAAAAAAAAAAAAAAAAAAAAAAAAAAAAAAAAAAAAAAAAAAAAAAAAAAAAAAAAAAAAAAAAAAAAAAAAAAAAAAAAAAAAAAAAAAAAAAAAAAAAAAAAAAAAAAAAAAAAAAAAAAAAAAAAAAAAAAAAAAAAAAAAAAAAAAAAAAAAAAAAAAAAAAAAAAAAAAAAAAAAAAAAAAAAAAAAAAAAAAAAAAAAAAAAAAAAAAAAAAAAAAAAAAAAAAAAAAAAAAAAAAAAAAAAAAAAAAAAAAAAAAAAAAAAAAAAAAAAAAAAAAAAAAAAAAAAAAAAAAAAAAAAAAAAAAAAAAAAAAAAAAAAAAAAAAAAAAAAAAAAAAAAAAAAAAAAAAAAAAAAAAAAAAAAAAAAAAAAAAAAAAAAAAAAAAAAAAAAAAAAAAAAAAAAAAAAAAAAAAAAAAAAAAAAAAAAAAAAAAAAAAAAAAAAAAAAAAAAAAAAAAAAAAAAAAAAAAAAAAAAAAAAAAAAAAAAAAAAAAAAAAAAAAAAAAAAAAAAAAAAAAAAAAAAAAAAAAAAAAAAAAAAAAAAAAAAAAAAAAAAAAAAAAAAAAAAAAAAAAAAAAAAAAAAAAAAAAAAAAAAAAAAAAAAAAAAAAAAAAAAAAAAAAAAAAAAAAAAAAAAAAAAAAAAAAAAAAAAAAAAAAAAAAAAAAAAAAAAAAAAAAAAAAAAAAAAAAAAAAAAAAAAAAAAAAAAAAAAAAAAAAAAAAAAAAAAAAAAAAAAAAAAAAAAAAAAAAAAAAAAAAAAAAAAAAAAAAAAAAAAAAAAAAAAAAAAAAAAAAAAAAAAAAAAAAAAAAAAAAAAAAAAAAAAAAAAAAAAAAAAAAAAAAAAAAAAAAAAAAAAAAAAAAAAAAAAAAAAAAAAAAAAAAAAAAAAAAAAAAAAAAAAAAAAAAAAAAAAAAAAAAAAAAAAAAAAAAAAAAAAAAAAAAAAAAAAAAAAAAAAAAAAAAAAAAAAAAAAAAAAAAAAAAAAAAAAAAAAAAAAAAAAAAAAAAAAAAAAAAAAAAAAAAAAAAAAAAAAAAAAAAAAAAAAAAAAAAAAAAAAAAAAAAAAAAAAAAAAAAAAAAAAAAAAAAAAAAAAAAAAAAAAAAAAAAAAAAAAAAAAAAAAAAAAAAAAAAAAAAAAAAAAAAAAAAAAAAAAAAAAAAAAAAAAAAAAAAAAAAAAAAAAAAAAAAAAAAAAAAAAAAAAAAAAAAAAAAAAAAAAAAAAAAAAAAAAAAAAAAAAAAAAAAAAAAAAAAAAAAAAAAAAAAAAAAAAAAAAAAAAAAAAAAAAAAAAAAAAAAAAAAAAAAAAAAAAAAAAAAAAAAAAAAAAAAAAAAAAAAAAAAAAAAAAAAAAAAAAAAAAAAAAAAAAAAAAAAAAAAAAAAAAAAAAAAAAAAAAAAAAAAAAAAAAAAAAAAAAAAAAAAAAAAAAAAAAAAAAAAAAAAAAAAAAAAAAAAAAAAAAAAAAAAAAAAAAAAAAAAAAAAAAAAAAAAAAAAAAAAAAAAAAAAAAAAAAAAAAAAAAAAAAAAAAAAAAAAAAAAAAAAAAAAAAAAAAAAAAAAAAAAAAAAAAAAAAAAAAAAAAAAAAAAAAAAAAAAAAAAAAAAAAAAAAAAAAAAAAAAAAAAAPAEJLHADMFAGNAEA

"Surface View 2" fire

"Surface View 2" setViewerMask 16383

"Surface View 2" setShadowStyle 0

"Surface View 2" setPickable 1

set hideNewModules 0

%Apply Filter By Measure module to filter objects into a label or binary image given a target measure and a filter criterion

create measurefilter "Filter By Measure 2"

"Filter By Measure 2" setIconPosition 160 226

"Filter By Measure 2" setVar "CustomHelp" {measurefilter.html}

"Filter By Measure 2" interpretation setValue 0

"Filter By Measure 2" outputLocation setIndex 0 0

"Filter By Measure 2" inputImage connect "AD.Optimized.view.thresholded"

"Filter By Measure 2" inputIntensityImage connect "AD.Optimized.view.am"

labelMeasure setAttributes feret2d 0 18 36 54 72 90 108 126 144 162

labelMeasure setAttributes feret3d 31

labelMeasure setAttributes cooccurrence 0 0

labelMeasure setAttributes histogram 1 0 255 1

labelMeasure setAttributes quantile 0.1 0.1 0.1 0.1 0.1 0.1

labelMeasure setAttributes breadth3d 10

labelMeasure setAttributes neighborCount 0 5 0

"Filter By Measure 2" measure setState {Area3d}

"Filter By Measure 2" measureFilterType setIndex 0 0

"Filter By Measure 2" numberOfPatterns setMinMax 0 0 2147483648

"Filter By Measure 2" numberOfPatterns setValue 0 1

"Filter By Measure 2" applyTransformToResult 1

"Filter By Measure 2" fire

"Filter By Measure 2" setViewerMask 16383

"Filter By Measure 2" setPickable 1

set hideNewModules 0

[ load ${SCRIPTDIR}/AD.Optimized.view.labels.am ] setLabel "AD.Optimized.view.labels.am"

"AD.Optimized.view.labels.am" setIconPosition 20 1594

"AD.Optimized.view.labels.am" master connect "Filter By Measure 2" "ImgOut" 0

"AD.Optimized.view.labels.am" sharedColormap connect "labels.am"

"AD.Optimized.view.labels.am" sharedColormap setDefaultColor 0.8 0.8 0.8

"AD.Optimized.view.labels.am" sharedColormap setDefaultAlpha 0.500000

"AD.Optimized.view.labels.am" sharedColormap activateLocalRange 1

"AD.Optimized.view.labels.am" sharedColormap setLocalMinMax 1.000000 8.000000

"AD.Optimized.view.labels.am" sharedColormap enableAlpha 1

"AD.Optimized.view.labels.am" sharedColormap enableAlphaToggle 1

"AD.Optimized.view.labels.am" sharedColormap setAutoAdjustRangeMode 1

"AD.Optimized.view.labels.am" fire

"AD.Optimized.view.labels.am" primary setIndex 0 0

"AD.Optimized.view.labels.am" fire

"AD.Optimized.view.labels.am" setViewerMask 16383

set hideNewModules 0

%Apply Volume Rendering module to allow for real-time rendering

create HxVolumeRenderingSettings "Volume Rendering Settings 4"

"Volume Rendering Settings 4" setViewerMask 0

"Volume Rendering Settings 4" setIconPosition 649 1594

"Volume Rendering Settings 4" setVar "CustomHelp" {HxVolumeRenderingSettings}

set hideNewModules 0

"Volume Rendering Settings 4" data connect "AD.Optimized.view.labels.am"

"Volume Rendering Settings 4" fire

"Volume Rendering Settings 4" rendering setValue 0

"Volume Rendering Settings 4" fire

"Volume Rendering Settings 4" interpolationAdvanced setValue 0

"Volume Rendering Settings 4" composition setValue 0

"Volume Rendering Settings 4" moveLowResolutionScale setMinMax 1 10

"Volume Rendering Settings 4" moveLowResolutionScale setButtons 1

"Volume Rendering Settings 4" moveLowResolutionScale setEditButton 1

"Volume Rendering Settings 4" moveLowResolutionScale setIncrement 1

"Volume Rendering Settings 4" moveLowResolutionScale setValue 3

"Volume Rendering Settings 4" moveLowResolutionScale setSubMinMax 1 10

"Volume Rendering Settings 4" samplingQuality setMinMax 0 2

"Volume Rendering Settings 4" samplingQuality setButtons 0

"Volume Rendering Settings 4" samplingQuality setEditButton 1

"Volume Rendering Settings 4" samplingQuality setIncrement 0.133333

"Volume Rendering Settings 4" samplingQuality setValue 1

"Volume Rendering Settings 4" samplingQuality setSubMinMax 0 2

"Volume Rendering Settings 4" opacityThreshold setMinMax 0 1

"Volume Rendering Settings 4" opacityThreshold setButtons 0

"Volume Rendering Settings 4" opacityThreshold setEditButton 1

"Volume Rendering Settings 4" opacityThreshold setIncrement 0.05

"Volume Rendering Settings 4" opacityThreshold setValue 0

"Volume Rendering Settings 4" opacityThreshold setSubMinMax 0 1

"Volume Rendering Settings 4" optimizations setValue 0 0

"Volume Rendering Settings 4" optimizations setToggleVisible 0 1

"Volume Rendering Settings 4" optimizations setValue 1 1

"Volume Rendering Settings 4" optimizations setToggleVisible 1 1

"Volume Rendering Settings 4" optimizations setValue 2 0

"Volume Rendering Settings 4" optimizations setToggleVisible 2 1

"Volume Rendering Settings 4" quality setValue 1

"Volume Rendering Settings 4" fire

"Volume Rendering Settings 4" artifactsReduction setValue 0 0

"Volume Rendering Settings 4" artifactsReduction setToggleVisible 0 1

"Volume Rendering Settings 4" artifactsReduction setValue 1 0

"Volume Rendering Settings 4" artifactsReduction setToggleVisible 1 1

"Volume Rendering Settings 4" lighting setState {item 0 1 item 1 1 }

"Volume Rendering Settings 4" gradient setState {item 0 1 item 2 9.99999974737875e-05 }

"Volume Rendering Settings 4" effects setValue 0 0

"Volume Rendering Settings 4" effects setToggleVisible 0 1

"Volume Rendering Settings 4" effects setValue 1 0

"Volume Rendering Settings 4" effects setToggleVisible 1 1

"Volume Rendering Settings 4" effects setValue 2 0

"Volume Rendering Settings 4" effects setToggleVisible 2 1

"Volume Rendering Settings 4" effects setValue 3 1

"Volume Rendering Settings 4" effects setToggleVisible 3 1

"Volume Rendering Settings 4" edgeEnhancement setMinMax 0 -3.40282346638529e+038 3.40282346638529e+038

"Volume Rendering Settings 4" edgeEnhancement setValue 0 9.99999974737875e-005

"Volume Rendering Settings 4" edge2D setState {item 1 0.100000001490116 item 3 0.100000001490116 item 5 1 }

"Volume Rendering Settings 4" boundaryOpacity setMinMax 0 -3.40282346638529e+038 3.40282346638529e+038

"Volume Rendering Settings 4" boundaryOpacity setValue 0 2.5

"Volume Rendering Settings 4" boundaryOpacity setMinMax 1 -3.40282346638529e+038 3.40282346638529e+038

"Volume Rendering Settings 4" boundaryOpacity setValue 1 2.5

"Volume Rendering Settings 4" material setIndex 0 4

"Volume Rendering Settings 4" lightingStyle setIndex 0 1

"Volume Rendering Settings 4" toneMapping setIndex 0 0

"Volume Rendering Settings 4" depthOfField setValue 0

"Volume Rendering Settings 4" blurFactor setMinMax 0.00999999977648258 1

"Volume Rendering Settings 4" blurFactor setButtons 0

"Volume Rendering Settings 4" blurFactor setEditButton 1

"Volume Rendering Settings 4" blurFactor setIncrement 0.066

"Volume Rendering Settings 4" blurFactor setValue 0.01

"Volume Rendering Settings 4" blurFactor setSubMinMax 0.00999999977648258 1

"Volume Rendering Settings 4" specularColor setMinMax 0 1

"Volume Rendering Settings 4" specularColor setButtons 0

"Volume Rendering Settings 4" specularColor setEditButton 1

"Volume Rendering Settings 4" specularColor setIncrement 0.1

"Volume Rendering Settings 4" specularColor setValue 0.4

"Volume Rendering Settings 4" specularColor setSubMinMax 0 1

"Volume Rendering Settings 4" shininess setMinMax 0 1

"Volume Rendering Settings 4" shininess setButtons 0

"Volume Rendering Settings 4" shininess setEditButton 1

"Volume Rendering Settings 4" shininess setIncrement 0.1

"Volume Rendering Settings 4" shininess setValue 0.4

"Volume Rendering Settings 4" shininess setSubMinMax 0 1

"Volume Rendering Settings 4" fire

"Volume Rendering Settings 4" setViewerMask 16383

"Volume Rendering Settings 4" setPickable 1

set hideNewModules 0

create HxVolumeRender2 "Volume Rendering 4"

"Volume Rendering 4" setIconPosition 649 1620

"Volume Rendering 4" setVar "CustomHelp" {HxVolumeRender2}

"Volume Rendering 4" data connect "AD.Optimized.view.labels.am"

"Volume Rendering 4" volumeRenderingSettings connect "Volume Rendering Settings 4"

"Volume Rendering 4" fire

"Volume Rendering 4" colormap connect "labels.am"

"Volume Rendering 4" colormap setDefaultColor 1 1 1

"Volume Rendering 4" colormap setDefaultAlpha 0.500000

"Volume Rendering 4" colormap activateLocalRange 1

"Volume Rendering 4" colormap setLocalMinMax 1.000000 8.000000

"Volume Rendering 4" colormap enableAlpha 1

"Volume Rendering 4" colormap enableAlphaToggle 1

"Volume Rendering 4" colormap setAutoAdjustRangeMode 1

"Volume Rendering 4" fire

"Volume Rendering 4" colormapLookup setValue 2

"Volume Rendering 4" gamma setMinMax 0.100000001490116 8

"Volume Rendering 4" gamma setButtons 0

"Volume Rendering 4" gamma setEditButton 1

"Volume Rendering 4" gamma setIncrement 0.526667

"Volume Rendering 4" gamma setValue 3

"Volume Rendering 4" gamma setSubMinMax 0.100000001490116 8

"Volume Rendering 4" alphaScale setMinMax 0 1

"Volume Rendering 4" alphaScale setButtons 0

"Volume Rendering 4" alphaScale setEditButton 1

"Volume Rendering 4" alphaScale setIncrement 0.1

"Volume Rendering 4" alphaScale setValue 1

"Volume Rendering 4" alphaScale setSubMinMax 0 1

"Volume Rendering 4" channelSelector setState {2 }

"Volume Rendering 4" fire

"Volume Rendering 4" setViewerMask 16382

"Volume Rendering 4" setPickable 1

set hideNewModules 0

%Apply Generate Surface module to compute a triangular approximation of the interfaces between different regions or materials in a Label Field with either uniform or stacked coordinates

create HxGMC "Generate Surface 3"

"Generate Surface 3" setIconPosition 160 1630

"Generate Surface 3" setVar "CustomHelp" {HxGMC}

"Generate Surface 3" data connect "AD.Optimized.view.labels.am"

"Generate Surface 3" fire

"Generate Surface 3" smoothing setIndex 0 3

"Generate Surface 3" smoothingExtent setMinMax 1 9

"Generate Surface 3" smoothingExtent setButtons 0

"Generate Surface 3" smoothingExtent setEditButton 1

"Generate Surface 3" smoothingExtent setIncrement 0.533333

"Generate Surface 3" smoothingExtent setValue 3

"Generate Surface 3" smoothingExtent setSubMinMax 1 9

"Generate Surface 3" options setState {item 0 0 item 1 0 }

"Generate Surface 3" borderOnOff setValue 1

"Generate Surface 3" algorithmMode setIndex 0 0

"Generate Surface 3" borderSettings setValue 0 1

"Generate Surface 3" borderSettings setToggleVisible 0 1

"Generate Surface 3" borderSettings setValue 1 0

"Generate Surface 3" borderSettings setToggleVisible 1 1

"Generate Surface 3" borderSettings setValue 2 0

"Generate Surface 3" borderSettings setToggleVisible 2 1

"Generate Surface 3" borderSettings setValue 3 0

"Generate Surface 3" borderSettings setToggleVisible 3 0

"Generate Surface 3" materialList setIndex 0 0

"Generate Surface 3" smoothMaterial setIndex 0 0

"Generate Surface 3" applyTransformToResult 1

"Generate Surface 3" fire

"Generate Surface 3" setViewerMask 16383

"Generate Surface 3" setPickable 1

set hideNewModules 0

[ load ${SCRIPTDIR}/AD.Optimized.view.surf.am ] setLabel "AD.Optimized.view.surf.am"

"AD.Optimized.view.surf.am" setIconPosition 20 1666

"AD.Optimized.view.surf.am" master connect "Generate Surface 3" "result" 0

"AD.Optimized.view.surf.am" fire

"AD.Optimized.view.surf.am" LevelOfDetail setMinMax -1 -1

"AD.Optimized.view.surf.am" LevelOfDetail setButtons 1

"AD.Optimized.view.surf.am" LevelOfDetail setEditButton 1

"AD.Optimized.view.surf.am" LevelOfDetail setIncrement 1

"AD.Optimized.view.surf.am" LevelOfDetail setValue -1

"AD.Optimized.view.surf.am" LevelOfDetail setSubMinMax -1 -1

"AD.Optimized.view.surf.am" fire

"AD.Optimized.view.surf.am" setViewerMask 16383

set hideNewModules 0

%Apply Surface View module to visualize triangular surfaces, i.e., data objects of type Surface

create HxDisplaySurface "Surface View 3"

"Surface View 3" setIconPosition 807 1666

"Surface View 3" setVar "CustomHelp" {HxDisplaySurface}

"Surface View 3" data connect "AD.Optimized.view.surf.am"

"Surface View 3" colormap disconnect

"Surface View 3" colormap setDefaultColor 1 0.1 0.1

"Surface View 3" colormap setDefaultAlpha 0.500000

"Surface View 3" colormap activateLocalRange 1

"Surface View 3" colormap setLocalMinMax 0.000000 1.000000

"Surface View 3" colormap enableAlpha 1

"Surface View 3" colormap enableAlphaToggle 1

"Surface View 3" colormap setAutoAdjustRangeMode 1

"Surface View 3" fire

"Surface View 3" drawStyle setValue 1

"Surface View 3" fire

"Surface View 3" drawStyle setSpecularLighting 1

"Surface View 3" drawStyle setTexture 1

"Surface View 3" drawStyle setAlphaMode 1

"Surface View 3" drawStyle setCullingMode 0

"Surface View 3" drawStyle setNormalBinding 0

"Surface View 3" drawStyle setSortingMode 1

"Surface View 3" drawStyle setLineWidth 0.000000

"Surface View 3" drawStyle setOutlineColor 0 0 0.2

"Surface View 3" textureWrap setIndex 0 1

"Surface View 3" selectionMode setIndex 0 0

"Surface View 3" patch setMinMax 0 2

"Surface View 3" patch setButtons 1

"Surface View 3" patch setEditButton 1

"Surface View 3" patch setIncrement 1

"Surface View 3" patch setValue 0

"Surface View 3" patch setSubMinMax 0 2

"Surface View 3" boundaryId setIndex 0 -1

"Surface View 3" materials setIndex 0 1

"Surface View 3" materials setIndex 1 0

"Surface View 3" colorMode setIndex 0 5

"Surface View 3" baseTrans setMinMax 0 1

"Surface View 3" baseTrans setButtons 0

"Surface View 3" baseTrans setEditButton 1

"Surface View 3" baseTrans setIncrement 0.1

"Surface View 3" baseTrans setValue 0.8

"Surface View 3" baseTrans setSubMinMax 0 1

"Surface View 3" VRMode setIndex 0 0

"Surface View 3" colorFieldMappingType setValue 0

"Surface View 3" setLighting 1

"Surface View 3" fire

"Surface View 3" hideBox 1

"Surface View 3" selectTriangles zab HIJMONMBABABAAAAAIACCAJPPFHPJFAOOIACNAACAAAAAAAAAAAAAAAAAAAAAAAAAAAAAAAAAAAAAAAAAAAAAAAAAAAAAAAAAAAAAAAAAAAAAAAAAAAAAAAAAAAAAAAAAAAAAAAAAAAAAAAAAAAAAAAAAAAAAAAAAAAAAAAAAAAAAAAAAAAAAAAAAAAAAAAAAAAAAAAAAAAAAAAAAAAAAAAAAAAAAAAAAAAAAAAAAAAAAAAAAAAAAAAAAAAAAAAAAAAAAAAAAAAAAAAAAAAAAAAAAAAAAAAAAAAAAAAAAAAAAAAAAAAAAAAAAAAAAAAAAAAAAAAAAAAAAAAAAAAAAAAAAAAAAAAAAAAAAAAAAAAAAAAAAAAAAAAAAAAAAAAAAAAAAAAAAAAAAAAAAAAAAAAAAAAAAAAAAAAAAAAAAAAAAAAAAAAAAAAAAAAAAAAAAAAAAAAAAAAAAAAAAAAAAAAAAAAAAAAAAAAAAAAAAAAAAAAAAAAAAAAAAAAAAAAAAAAAAAAAAAAAAAAAAAAAAAAAAAAAAAAAAAAAAAAAAAAAAAAAAAAAAAAAAAAAAAAAAAAAAAAAAAAAAAAAAAAAAAAAAAAAAAAAAAAAAAAAAAAAAAAAAAAAAAAAAAAAAAAAAAAAAAAAAAAAAAAAAAAAAAAAAAAAAAAAAAAAAAAAAAAAAAAAAAAAAAAAAAAAAAAAAAAAAAAAAAAAAAAAAAAAAAAAAAAAAAAAAAAAAAAAAAAAAAAAAAAAAAAAAAAAAAAAAAAAAAAAAAAAAAAAAAAAAAAAAAAAAAAAAAAAAAAAAAAAAAAAAAAAAAAAAAAAAAAAAAAAAAAAAAAAAAAAAAAAAAAAAAAAAAAAAAAAAAAAAAAAAAAAAAAAAAAAAAAAAAAAAAAAAAAAAAAAAAAAAAAAAAAAAAAAAAAAAAAAAAAAAAAAAAAAAAAAAAAAAAAAAAAAAAAAAAAAAAAAAAAAAAAAAAAAAAAAAAAAAAAAAAAAAAAAAAAAAAAAAAAAAAAAAAAAAAAAAAAAAAAAAAAAAAAAAAAAAAAAAAAAAAAAAAAAAAAAAAAAAAAAAAAAAAAAAAAAAAAAAAAAAAAAAAAAAAAAAAAAAAAAAAAAAAAAAAAAAAAAAAAAAAAAAAAAAAAAAAAAAAAAAAAAAAAAAAAAAAAAAAAAAAAAAAAAAAAAAAAAAAAAAAAAAAAAAAAAAAAAAAAAAAAAAAAAAAAAAAAAAAAAAAAAAAAAAAAAAAAAAAAAAAAAAAAAAAAAAAAAAAAAAAAAAAAAAAAAAAAAAAAAAAAAAAAAAAAAAAAAAAAAAAAAAAAAAAAAAAAAAAAAAAAAAAAAAAAAAAAAAAAAAAAAAAAAAAAAAAAAAAAAAAAAAAAAAAAAAAAAAAAAAAAAAAAAAAAAAAAAAAAAAAAAAAAAAAAAAAAAAAAAAAAAAAAAAAAAAAAAAAAAAAAAAAAAAAAAAAAAAAAAAAAAAAAAAAAAAAAAAAAAAAAAAAAAAAAAAAAAAAAAAAAAAAAAAAAAAAAAAAAAAAAAAAAAAAAAAAAAAAAAAAAAAAAAAAAAAAAAAAAAAAAAAAAAAAAAAAAAAAAAAAAAAAAAAAAAAAAAAAAAAAAAAAAAAAAAAAAAAAAAAAAAAAAAAAAAAAAAAAAAAAAAAAAAAAAAAAAAAAAAAAAAAAAAAAAAAAAAAAAAAAAAAAAAAAAAAAAAAAAAAAAAAAAAAAAAAAAAAAAAAAAAAAAAAAAAAAAAAAAAAAAAAAAAAAAAAAAAAAAAAAAAAAAAAAAAAAAAAAAAAAAAAAAAAAAAAAAAAAAAAAAAAAAAAAAAAAAAAAAAAAAAAAAAAAAAAAAAAAAAAAAAAAAAAAAAAAAAAAAAAAAAAAAAAAAAAAAAAAAAAAAAAAAAAAAAAAAAAAAAAAAAAAAAAAAAAAAAAAAAAAAAAAAAAAAAAAAAAAAAAAAAAAAAAAAAAAAAAAAAAAAAAAAAAAAAAAAAAAAAAAAAAAAAAAAAAAAAAAAAAAAAAAAAAAAAAAAAAAAAAAAAAAAAAAAAAAAAAAAAAAAAAAAAAAAAAAAAAAAAAAAAAAAAAAAAAAAAAAAAAAAAAAAAAAAAAAAAAAAAAAAAAAAAAAAAAAAAAAAAAAAAAAAAAAAAAAAAAAAAAAAAAAAAAAAAAAAAAAAAAAAAAAAAAAAAAAAAAAAAAAAAAAAAAAAAAAAAAAAAAAAAAAAAAAAAAAAAAAAAAAAAAAAAAAAAAAAAAAAAAAAAAAAAAAAAAAAAAAAAAAAAAAAAAAAAAAAAAAAAAAAAAAAAAAAAAAAAAAAAAAAAAAAAAAAAAAAAAAAAAAAAAAAAAAAAAAAAAAAAAAAAAAAAAAAAAAAAAAAAAAAAAAAAAAAAAAAAAAAAAAAAAAAAAAAAAAAAAAAAAAAAAAAAAAAAAAAAAAAAAAAAAAAAAAAAAAAAAAAAAAAAAAAAAAAAAAAAAAAAAAAAAAAAAAAAAAAAAAAAAAAAAAAAAAAAAAAAAAAAAAAAAAAAAAAAAAAAAAAAAAAAAAAAAAAAAAAAAAAAAAAAAAAAAAAAAAAAAAAAAAAAAAAAAAAAAAAAAAAAAAAAAAAAAAAAAAAAAAAAAAAAAAAAAAAAAAAAAAAAAAAAAAAAAAAAAAAAAAAAAAAAAAAAAAAAAAAAAAAAAAAAAAAAAAAAAAAAAAAAAAAAAAAAAAAAAAAAAAAAAAAAAAAAAAAAAAAAAAAAAAAAAAAAAAAAAAAAAAAAAAAAAAAAAAAAAAAAAAAAAAAAAAAAAAAAAAAAAAAAAAAAAAAAAAAAAAAAAAAAAAAAAAAAAAAAAAAAAAAAAAAAAAAAAAAAAAAAAAAAAAAAAAAAAAAAAAAAAAAAAAAAAAAAAAAAAAAAAAAAAAAAAAAAAAAAAAAAAAAAAAAAAAAAAAAAAAAAAAAAAAAAAAAAAAAAAAAAAAAAAAAAAAAAAAAAAAAAAAAAAAAAAAAAAAAAAAAAAAAAAAAAAAAAAAAAAAAAAAAAAAAAAAAAAAAAAAAAAAAAAAAAAAAAAAAAAAAAAAAAAAAAAAAAAAAAAAAAAAAAAAAAAAAAAAAAAAAAAAAAAAAAAAAAAAAAAAAAAAAAAAAAAAAAAAAAAAAAAAAAAAAAAAAAAAAAAAAAAAAAAAAAAAAAAAAAAAAAAAAAAAAAAAAAAAAAAAAAAAAAAAAAAAAAAAAAAAAAAAAAAAAAAAAAAAAAAAAAAAAAAAAAAAAAAAAAAAAAAAAAAAAAAAAAAAAAAAAAAAAAAAAAAAAAAAAAAAAAAAAAAAAAAAAAAAAAAAAAAAAAAAAAAAAAAAAAAAAAAAAAAAAAAAAAAAAAAAAAAAAAAAAAAAAAAAAAAAAAAAAAAAAAAAAAAAAAAAAAAAAAAAAAAAAAAAAAAAAAAAAAAAAAAAAAAAAAAAAAAAAAAAAAAAAAAAAAAAAAAAAAAAAAAAAAAAAAAAAAAAAAAAAAAAAAAAAAAAAAAAAAAAAAAAAAAAAAAAAAAAAAAAAAAAAAAAAAAAAAAAAAAAAAAAAAAAAAAAAAAAAAAAAAAAAAAAAAAAAAAAAAAAAAAAAAAAAAAAAAAAAAAAAAAAAAAAAAAAAAAAAAAAAAAAAAAAAAAAAAAAAAAAAAAAAAAAAAAAAAAAAAAAAAAAAAAAAAAAAAAAAAAAAAAAAAAAAAAAAAAAAAAAAAAAAAAAAAAAAAAAAAAAAAAAAAAAAAAAAAAAAAAAAAAAAAAAAAAAAAAAAAAAAAAAAAAAAAAAAAAAAAAAAAAAAAAAAAAAAAAAAAAAAAAAAAAAAAAAAAAAAAAAAAAAAAAAAAAAAAAAAAAAAAAAAAAAAAAAAAAAAAAAAAAAAAAAAAAAAAAAAAAAAAAAAAAAAAAAAAAAAAAAAAAAAAAAAAAAAAAAAAAAAAAAAAAAAAAAAAAAAAAAAAAAAAAAAAAAAAAAAAAAAAAAAAAAAAAAAAAAAAAAAAAAAAAAAAAAAAAAAAAAAAAAAAAAAAAAAAAAAAAAAAAAAAAAAAAAAAAAAAAAAAAAAAAAAAAAAAAAAAAAAAAAAAAAAAAAAAAAAAAAAAAAAAAAAAAAAAAAAAAAAAAAAAAAAAAAAAAAAAAAAAAAAAAAAAAAAAAAAAAAAAAAAAAAAAAAAAAAAAAAAAAAAAAAAAAAAAAAAAAAAAAAAAAAAAAAAAAAAAAAAAAAAAAAAAAAAAAAAAAAAAAAAAAAAAAAAAAAAAAAAAAAAAAAAAAAAAAAAAAAAAAAAAAAAAAAAAAAAAAAAAAAAAAAAAAAAAAAAAAAAAAAAAAAAAAAAAAAAAAAAAAAAAAAAAAAAAAAAAAAAAAAAAAAAAAAAAAAAAAAAAAAAAAAAAAAAAAAAAAAAAAAAAAAAAAAAAAAAAAAAAAAAAAAAAAAAAAAAAAAAAAAAAAAAAAAAAAAAAAAAAAAAAAAAAAAAAAAAAAAAAAAAAAAAAAAAAAAAAAAAAAAAAAAAAAAAAAAAAAAAAAAAAAAAAAAAAAAAAAAAAAAAAAAAAAAAAAAAAAAAAAAAAAAAAAAAAAAAAAAAAAAAAAAAAAAAAAAAAAAAAAAAAAAAAAAAAAAAAAAAAAAAAAAAAAAAAAAAAAAAAAAAAAAAAAAAAAAAAAAAAAAAAAAAAAAAAAAAAAAAAAAAAAAAAAAAAAAAAAAAAAAAAAAAAAAAAAAAAAAAAAAAAAAAAAAAAAAAAAAAAAAAAAAAAAAAAAAAAAAAAAAAAAAAAAAAAAAAAAAAAAAAAAAAAAAAAAAAAAAAAAAAAAAAAAAAAAAAAAAAAAAAAAAAAAAAAAAAAAAAAAAAAAAAAAAAAAAAAAAAAAAAAAAAAAAAAAAAAAAAAAAAAAAAAAAAAAAAAAAAAAAAAAAAAAAAAAAAAAAAAAAAAAAAAAAAAAAAAAAAAAAAAAAAAAAAAAAAAAAAAAAAAAAAAAAAAAAAAAAAAAAAAAAAAAAAAAAAAAAAAAAAAAAAAAAAAAAAAAAAAAAAAAAAAAAAAAAAAAAAAAAAAAAAAAAAAAAAAAAAAAAAAAAAAAAAAAAAAAAAAAAAAAAAAAAAAAAAAAAAAAAAAAAAAAAAAAAAAAAAAAAAAAAAAAAAAAAAAAAAAAAAAAAAAAAAAAAAAAAAAAAAAAAAAAAAAAAAAAAAAAAAAAAAAAAAAAAAAAAAAAAAAAAAAAAAAAAAAAAAAAAAAAAAAAAAAAAAAAAAAAAAAAAAAAAAAAAAAAAAAAAAAAAAAAAAAAAAAAAAAAAAAAAAAAAAAAAAAAAAAAAAAAAAAAAAAAAAAAAAAAAAAAAAAAAAAAAAAAAAAAAAAAAAAAAAAAAAAAAAAAAAAAAAAAAAAAAAAAAAAAAAAAAAAAAAAAAAAAAAAAAAAAAAAAAAAAAAAAAAAAAAAAAAAAAAAAAAAAAAAAAAAAAAAAAAAAAAAAAAAAAAAAAAAAAAAAAAAAAAAAAAAAAAAAAAAAAAAAAAAAAAAAAAAAAAAAAAAAAAAAAAAAAAAAAAAAAAAAAAAAAAAAAAAAAAAAAAAAAAAAAAAAAAAAAAAAAAAAAAAAAAAAAAAAAAAAAAAAAAAAAAAAAAAAAAAAAAAAAAAAAAAAAAAAAAAAAAAAAAAAAAAAAAAAAAAAAAAAAAAAAAAAAAAAAAAAAAAAAAAAAAAAAAAAAAAAAAAAAAAAAAAAAAAAAAAAAAAAAAAAAAAAAAAAAAAAAAAAAAAAAAAAAAAAAAAAAAAAAAAAAAAAAAAAAAAAAAAAAAAAAAAAAAAAAAAAAAAAAAAAAAAAAAAAAAAAAAAAAAAAAAAAAAAAAAAAAAAAAAAAAAAAAAAAAAAAAAAAAAAAAAAAAAAAAAAAAAAAAAAAAAAAAAAAAAAAAAAAAAAAAAAAAAAAAAAAAAAAAAAAAAAAAAAAAAAAAAAAAAAAAAAAAAAAAAAAAAAAAAAAAAAAAAAAAAAAAAAAAAAAAAAAAAAAAAAAAAAAAAAAAAAAAAAAAAAAAAAAAAAAAAAAAAAAAAAAAAAAAAAAAAAAAAAAAAAAAAAAAAAAAAAAAAAAAAAAAAAAAAAAAAAAAAAAAAAAAAAAAAAAAAAAAAAAAAAAAAAAAAAAAAAAAAAAAAAAAAAAAAAAAAAAAAAAAAAAAAAAAAAAAAAAAAAAAAAAAAAAAAAAAAAAAAAAAAAAAAAAAAAAAAAAAAAAAAAAAAAAAAAAAAAAAAAAAAAAAAAAAAAAAAAAAAAAAAAAAAAAAAAAAAAAAAAAAAAAAAAAAAAAAAAAAAAAAAAAAAAAAAAAAAAAAAAAAAAAAAAAAAAAAAAAAAAAAAAAAAAAAAAAAAAAAAAAAAAAAAAAAAAAAAAAAAAAAAAAAAAAAAAAAAAAAAAAAAAAAAAAAAAAAAAAAAAAAAAAAAAAAAAAAAAAAAAAAAAAAAAAAAAAAAAAAAAAAAAAAAAAAAAAAAAAAAAAAAAAAAAAAAAAAAAAAAAAAAAAAAAAAAAAAAAAAAAAAAAAAAAAAAAAAAAAAAAAAAAAAAAAAAAAAAAAAAAAAAAAAAAAAAAAAAAAAAAAAAAAAAAAAAAAAAAAAAAAAAAAAAAAAAAAAAAAAAAAAAAAAAAAAAAAAAAAAAAAAAAAAAAAAAAAAAAAAAAAAAAAAAAAAAAAAAAAAAAAAAAAAAAAAAAAAAAAAAAAAAAAAAAAAAAAAAAAAAAAAAAAAAAAAAAAAAAAAAAAAAAAAAAAAAAAAAAAAAAAAAAAAAAAAAAAAAAAAAAAAAAAAAAAAAAAAAAAAAAAAAAAAAAAAAAAAAAAAAAAAAAAAAAAAAAAAAAAAAAAAAAAAAAAAAAAAAAAAAAAAAAAAAAAAAAAAAAAAAAAAAAAAAAAAAAAAAAAAAAAAAAAAAAAAAAAAAAAAAAAAAAAAAAAAAAAAAAAAAAAAAAAAAAAAAAAAAAAAAAAAAAAAAAAAAAAAAAAAAAAAAAAAAAAAAAAAAAAAAAAAAAAAAAAAAAAAAAAAAAAAAAAAAAAAAAAAAAAAAAAAAAAAAAAAAAAAAAAAAAAAAAAAAAAAAAAAAAAAAAAAAAAAAAAAAAAAAAAAAAAAAAAAAAAAAAAAAAAAAAAAAAAAAAAAAAAAAAAAAAAAAAAAAAAAAAAAAAAAAAAAAAAAAAAAAAAAAAAAAAAAAAAAAAAAAAAAAAAAAAAAAAAAAAAAAAAAAAAAAAAAAAAAAAAAAAAAAAAAAAAAAAAAAAAAAAAAAAAAAAAAAAAAAAAAAAAAAAAAAAAAAAAAAAAAAAAAAAAAAAAAAAAAAAAAAAAAAAAAAAAAAAAAAAAAAAAAAAAAAAAAAAAAAAAAAAAAAAAAAAAAAAAAAAAAAAAAAAAAAAAAAAAAAAAAAAAAAAAAAAAAAAAAAAAAAAAAAAAAAAAAAAAAAAAAAAAAAAAAAAAAAAAAAAAAAAAAAAAAAAAAAAAAAAAAAAAAAAAAAAAAAAAAAAAAAAAAAAAAAAAAAAAAAAAAAAAAAAAAAAAAAAAAAAAAAAAAAAAAAAAAAAAAAAAAAAAAAAAAAAAAAAAAAAAAAAAAAAAAAAAAAAAAAAAAAAAAAAAAAAAAAAAAAAAAAAAAAAAAAAAAAAAAAAAAAAAAAAAAAAAAAAAAAAAAAAAAAAAAAAAAAAAAAAAAAAAAAAAAAAAAAAAAAAAAAAAAAAAAAAAAAAAAAAAAAAAAAAAAAAAAAAAAAAAAAAAAAAAAAAAAAAAAAAAAAAAAAAAAAAAAAAAAAAAAAAAAAAAAAAAAAAAAAAAAAAAAAAAAAAAAAAAAAAAAAAAAAAAAAAAAAAAAAAAAAAAAAAAAAAAAAAAAAAAAAAAAAAAAAAAAAAAAAAAAAAAAAAAAAAAAAAAAAAAAAAAAAAAAAAAAAAAAAAAAAAAAAAAAAAAAAAAAAAAAAAAAAAAAAAAAAAAAAAAAAAAAAAAAAAAAAAAAAAAAAAAAAAAAAAAAAAAAAAAAAAAAAAAAAAAAAAAAAAAAAAAAAAAAAAAAAAAAAAAAAAAAAAAAAAAAAAAAAAAAAAAAAAAAAAAAAAAAAAAAAAAAAAAAAAAAAAAAAAAAAAAAAAAAAAAAAAAAAAAAAAAAAAAAAAAAAAAAHMEJGOJDPODKDK

"Surface View 3" fire

"Surface View 3" setViewerMask 16382

"Surface View 3" setShadowStyle 0

"Surface View 3" setPickable 1

set hideNewModules 0

%Apply ROI Box module to select region of interest (ROI)

create HxSelectRoi "ROI Box"

"ROI Box" setIconPosition 890 324

"ROI Box" setVar "CustomHelp" {HxSelectRoi}

"ROI Box" data connect "AD.Optimized.view.inverted"

"ROI Box" fire

"ROI Box" minimum setMinMax 0 1355.19995117188 2517.89990234375

"ROI Box" minimum setValue 0 1355.19995117188

"ROI Box" minimum setMinMax 1 2190.64990234375 2931.60009765625

"ROI Box" minimum setValue 1 2190.64990234375

"ROI Box" minimum setMinMax 2 0 199

"ROI Box" minimum setValue 2 100

"ROI Box" maximum setMinMax 0 1355.19995117188 2517.89990234375

"ROI Box" maximum setValue 0 2517.89990234375

"ROI Box" maximum setMinMax 1 2190.64990234375 2931.60009765625

"ROI Box" maximum setValue 1 2931.60009765625

"ROI Box" maximum setMinMax 2 0 199

"ROI Box" maximum setValue 2 120

"ROI Box" options setValue 0 1

"ROI Box" options setToggleVisible 0 1

"ROI Box" fire

"ROI Box" setViewerMask 16383

"ROI Box" setPickable 1

set hideNewModules 0

%Apply Volume Rendering module to allow for real-time rendering

create HxVolumeRenderingSettings "Volume Rendering Settings 5"

"Volume Rendering Settings 5" setViewerMask 0

"Volume Rendering Settings 5" setIconPosition 651 262

"Volume Rendering Settings 5" setVar "CustomHelp" {HxVolumeRenderingSettings}

set hideNewModules 0

"Volume Rendering Settings 5" data connect "AD.Optimized.view.inverted"

"Volume Rendering Settings 5" ROI connect "ROI Box"

"Volume Rendering Settings 5" fire

"Volume Rendering Settings 5" rendering setValue 0

"Volume Rendering Settings 5" fire

"Volume Rendering Settings 5" interpolationAdvanced setValue 1

"Volume Rendering Settings 5" composition setValue 0

"Volume Rendering Settings 5" moveLowResolutionScale setMinMax 1 10

"Volume Rendering Settings 5" moveLowResolutionScale setButtons 1

"Volume Rendering Settings 5" moveLowResolutionScale setEditButton 1

"Volume Rendering Settings 5" moveLowResolutionScale setIncrement 1

"Volume Rendering Settings 5" moveLowResolutionScale setValue 3

"Volume Rendering Settings 5" moveLowResolutionScale setSubMinMax 1 10

"Volume Rendering Settings 5" samplingQuality setMinMax 0 2

"Volume Rendering Settings 5" samplingQuality setButtons 0

"Volume Rendering Settings 5" samplingQuality setEditButton 1

"Volume Rendering Settings 5" samplingQuality setIncrement 0.133333

"Volume Rendering Settings 5" samplingQuality setValue 1

"Volume Rendering Settings 5" samplingQuality setSubMinMax 0 2

"Volume Rendering Settings 5" opacityThreshold setMinMax 0 1

"Volume Rendering Settings 5" opacityThreshold setButtons 0

"Volume Rendering Settings 5" opacityThreshold setEditButton 1

"Volume Rendering Settings 5" opacityThreshold setIncrement 0.05

"Volume Rendering Settings 5" opacityThreshold setValue 0

"Volume Rendering Settings 5" opacityThreshold setSubMinMax 0 1

"Volume Rendering Settings 5" optimizations setValue 0 0

"Volume Rendering Settings 5" optimizations setToggleVisible 0 1

"Volume Rendering Settings 5" optimizations setValue 1 1

"Volume Rendering Settings 5" optimizations setToggleVisible 1 1

"Volume Rendering Settings 5" optimizations setValue 2 0

"Volume Rendering Settings 5" optimizations setToggleVisible 2 1

"Volume Rendering Settings 5" quality setValue 1

"Volume Rendering Settings 5" fire

"Volume Rendering Settings 5" artifactsReduction setValue 0 0

"Volume Rendering Settings 5" artifactsReduction setToggleVisible 0 1

"Volume Rendering Settings 5" artifactsReduction setValue 1 1

"Volume Rendering Settings 5" artifactsReduction setToggleVisible 1 1

"Volume Rendering Settings 5" lighting setState {item 0 2 item 1 0 }

"Volume Rendering Settings 5" gradient setState {item 0 1 item 2 9.99999974737875e-05 }

"Volume Rendering Settings 5" effects setValue 0 1

"Volume Rendering Settings 5" effects setToggleVisible 0 1

"Volume Rendering Settings 5" effects setValue 1 0

"Volume Rendering Settings 5" effects setToggleVisible 1 1

"Volume Rendering Settings 5" effects setValue 2 0

"Volume Rendering Settings 5" effects setToggleVisible 2 1

"Volume Rendering Settings 5" effects setValue 3 0

"Volume Rendering Settings 5" effects setToggleVisible 3 1

"Volume Rendering Settings 5" edgeEnhancement setMinMax 0 -3.40282346638529e+038 3.40282346638529e+038

"Volume Rendering Settings 5" edgeEnhancement setValue 0 9.99999974737875e-005

"Volume Rendering Settings 5" edge2D setState {item 1 0.100000001490116 item 3 0.100000001490116 item 5 1 }

"Volume Rendering Settings 5" boundaryOpacity setMinMax 0 -3.40282346638529e+038 3.40282346638529e+038

"Volume Rendering Settings 5" boundaryOpacity setValue 0 2.5

"Volume Rendering Settings 5" boundaryOpacity setMinMax 1 -3.40282346638529e+038 3.40282346638529e+038

"Volume Rendering Settings 5" boundaryOpacity setValue 1 2.5

"Volume Rendering Settings 5" material setIndex 0 4

"Volume Rendering Settings 5" lightingStyle setIndex 0 1

"Volume Rendering Settings 5" toneMapping setIndex 0 0

"Volume Rendering Settings 5" depthOfField setValue 0

"Volume Rendering Settings 5" blurFactor setMinMax 0.00999999977648258 1

"Volume Rendering Settings 5" blurFactor setButtons 0

"Volume Rendering Settings 5" blurFactor setEditButton 1

"Volume Rendering Settings 5" blurFactor setIncrement 0.066

"Volume Rendering Settings 5" blurFactor setValue 0.01

"Volume Rendering Settings 5" blurFactor setSubMinMax 0.00999999977648258 1

"Volume Rendering Settings 5" specularColor setMinMax 0 1

"Volume Rendering Settings 5" specularColor setButtons 0

"Volume Rendering Settings 5" specularColor setEditButton 1

"Volume Rendering Settings 5" specularColor setIncrement 0.1

"Volume Rendering Settings 5" specularColor setValue 0.4

"Volume Rendering Settings 5" specularColor setSubMinMax 0 1

"Volume Rendering Settings 5" shininess setMinMax 0 1

"Volume Rendering Settings 5" shininess setButtons 0

"Volume Rendering Settings 5" shininess setEditButton 1

"Volume Rendering Settings 5" shininess setIncrement 0.1

"Volume Rendering Settings 5" shininess setValue 0.4

"Volume Rendering Settings 5" shininess setSubMinMax 0 1

"Volume Rendering Settings 5" fire

"Volume Rendering Settings 5" setViewerMask 16383

"Volume Rendering Settings 5" setPickable 1

set hideNewModules 0

create HxVolumeRender2 "Volume Rendering 5"

"Volume Rendering 5" setIconPosition 651 288

"Volume Rendering 5" setVar "CustomHelp" {HxVolumeRender2}

"Volume Rendering 5" data connect "AD.Optimized.view.inverted"

"Volume Rendering 5" volumeRenderingSettings connect "Volume Rendering Settings 5"

"Volume Rendering 5" fire

"Volume Rendering 5" colormap connect "volrenGreen.col"

"Volume Rendering 5" colormap setDefaultColor 1 1 1

"Volume Rendering 5" colormap setDefaultAlpha 0.500000

"Volume Rendering 5" colormap activateLocalRange 1

"Volume Rendering 5" colormap setLocalMinMax 115.000000 140.000000

"Volume Rendering 5" colormap enableAlpha 1

"Volume Rendering 5" colormap enableAlphaToggle 1

"Volume Rendering 5" colormap setAutoAdjustRangeMode 1

"Volume Rendering 5" fire

"Volume Rendering 5" colormapLookup setValue 2

"Volume Rendering 5" gamma setMinMax 0.100000001490116 8

"Volume Rendering 5" gamma setButtons 0

"Volume Rendering 5" gamma setEditButton 1

"Volume Rendering 5" gamma setIncrement 0.526667

"Volume Rendering 5" gamma setValue 3

"Volume Rendering 5" gamma setSubMinMax 0.100000001490116 8

"Volume Rendering 5" alphaScale setMinMax 0 1

"Volume Rendering 5" alphaScale setButtons 0

"Volume Rendering 5" alphaScale setEditButton 1

"Volume Rendering 5" alphaScale setIncrement 0.1

"Volume Rendering 5" alphaScale setValue 1

"Volume Rendering 5" alphaScale setSubMinMax 0 1

"Volume Rendering 5" channelSelector setState {2 }

"Volume Rendering 5" fire

"Volume Rendering 5" setViewerMask 16383

"Volume Rendering 5" setPickable 1

set hideNewModules 0

viewer 0 setCameraOrientation 0.380538 -0.91588 0.127886 3.35109

viewer 0 setCameraPosition 2090.26 2738.61 -5.10982

viewer 0 setCameraFocalDistance 117.701

viewer 0 setCameraNearDistance 4.06963

viewer 0 setCameraFarDistance 269.507

viewer 0 setCameraType perspective

viewer 0 setCameraHeightAngle 44.9023

viewer 0 setAutoRedraw 1

viewer 0 redraw

correlationPanel closeTabs

correlationPanel setTabName 0 "Correlation 1"

correlationPanel 0 setLogScalingOnAxis 0 0

correlationPanel 0 setLogScalingOnAxis 1 0

histogramPanel closeTabs

histogramPanel setTabName 0 "Histogram 1"

histogramPanel 0 setLogScalingOnAxis 0 0

histogramPanel 0 setLogScalingOnAxis 1 1

AD.Optimized.view3.Label-Analysis.am table hit

AD.Optimized.view3.Label-Analysis.am fire

AD.Optimized.view3.Analysis-Filter.am table hit

AD.Optimized.view3.Analysis-Filter.am fire

theObjectPool setSelectionOrder AD.Optimized.view.am
